# Zeolite-promoted platinum catalyst for efficient reduction of nitrogen oxides with hydrogen

**DOI:** 10.1038/s41467-024-52382-7

**Published:** 2024-09-12

**Authors:** Shaohua Xie, Liping Liu, Yuejin Li, Kailong Ye, Daekun Kim, Xing Zhang, Hongliang Xin, Lu Ma, Steven N. Ehrlich, Fudong Liu

**Affiliations:** 1 Department of Chemical and Environmental Engineering, Bourns College of Engineering, Center for Environmental Research and Technology (CE-CERT), Materials Science and Engineering (MSE) Program, University of California, Riverside, CA USA; 2 Department of Civil, Environmental, and Construction Engineering, Catalysis Cluster for Renewable Energy and Chemical Transformations (REACT), NanoScience Technology Center (NSTC), University of Central Florida, Orlando, FL USA; 3 Department of Chemical Engineering, Virginia Polytechnic Institute and State University, Blacksburg, VA USA; 4 BASF Environmental Catalyst and Metal Solutions, Iselin, NJ USA; 5 National Synchrotron Light Source II (NSLS-II), Brookhaven National Laboratory, Upton, New York, NY USA

**Keywords:** Pollution remediation, Atmospheric chemistry, Heterogeneous catalysis

## Abstract

Internal combustion engine fueled by carbon-free hydrogen (H_2_-ICE) offers a promising alternative for sustainable transportation. Herein, we report a facile and universal strategy through the physical mixing of Pt catalyst with zeolites to significantly improve the catalytic performance in the selective catalytic reduction of nitrogen oxides (NO_*x*_) with H_2_ (H_2_-SCR), a process aiming at NO_*x*_ removal from H_2_-ICE. Via the physical mixing of Pt/TiO_2_ with Y zeolite (Pt/TiO_2_ + Y), a remarkable enhancement of NO_*x*_ reduction activity and N_2_ selectivity was simultaneously achieved. The incorporation of Y zeolite effectively captured the in-situ generated water, fostering a water-rich environment surrounding the Pt active sites. This environment weakened the NO adsorption while concurrently promoting the H_2_ activation, leading to the strikingly elevated H_2_-SCR activity and N_2_ selectivity on Pt/TiO_2_ + Y catalyst. This study provides a unique, easy and sustainable physical mixing approach to achieve proficient heterogeneous catalysis for environmental applications.

## Abstract (web summary)

The integration of Pt-oxide catalyst systems with zeolites demonstrates considerable promise for reducing nitrogen oxide emissions at low temperatures from fossil-fuel or hydrogen-powered internal combustion engines.

## Introduction

The transportation sector has a considerable impact on global climate change^1^, being responsible for nearly 24% of the world’s CO_2_ emissions stemming from fossil fuel combustion^2^. Consequently, it is imperative to prioritize substantial CO_2_ reduction within this sector. While electric powertrains powered by renewable energy hold promise, their environmental cost and limited energy capacity for heavy-duty vehicles pose significant challenges for widespread application^3^. There is another viable avenue lies in the adoption of internal combustion engines (ICE) operating on carbon-free hydrogen (H_2_), which presents a promising alternative for sustainable transportation^3,4^. During the H_2_ combustion process, nitrogen oxides (NO_*x*_) are the primary environmental pollutants^5,6^. Selective catalytic reduction (SCR) of NO_*x*_ is one of the most efficient and widely used technologies for NO_*x*_ abatement in excess oxygen^6,7^. For H_2_-ICE applications, H_2_ extracted from the fuel tank can serve directly as a reducing agent for the SCR of NO_*x*_ (H_2_-SCR)^8^. This approach may offer significant economic and environmental benefits. However, to make this technique viable, the key issue to be solved is the development of robust H_2_-SCR catalyst systems, which can demonstrate excellent low-temperature NO_*x*_ reduction activity and N_2_ selectivity simultaneously.

Supported platinum (Pt) and palladium (Pd) catalysts have been extensively investigated for H_2_-SCR reaction^6,9,10^. Notably, Pt catalysts have shown great promise with their superior low-temperature (<150 ^o^C) activity comparing to Pd catalysts^11,12^, although there is urgent need for significant improvement in N_2_ selectivity^13^. H_2_ activation was considered as one of the most critical factors on Pt catalysts that could profoundly influence the H_2_-SCR performance^14^. Improving H_2_ activation and sustaining abundant *H species on Pt catalysts could positively promote the NO dissociation^15–17^, which has been reported as the rate-determining step for the H_2_-SCR reaction^18,19^. Additionally, this enhancement could also facilitate the formation of NH_*x*_ species, which, in some cases, have been found beneficial for the H_2_-SCR reaction^14,20–23^. Currently, substantial efforts have been dedicated towards increasing the presence of metallic Pt species^24,25^, as it plays a crucial role in H_2_ activation. Studies have reported that specific additives could substantially enhance the NO_*x*_ reduction activity by reducing the Pt valence. For instance, the addition of Mo and Na to Pt/SiO_2_^26^ and the introduction of Ti species into Pt/MCM-41^13^ resulted in the lowered Pt valence state, leading to widened temperature window for NO_*x*_ conversion. Additionally, the acidity or basicity of supports also strongly influenced the dispersion and chemical state of Pt^27,28^, with acidic supports being beneficial for the formation of metallic Pt species therefore promoting the H_2_-SCR performance^27,29^. Such strategies involving the chemical modification of Pt catalysts to form more metallic Pt species were mainly intent to enhance the H_2_ activation. However, it was observed that the presence of metallic Pt species usually favored the NO adsorption over H_2_ adsorption, inevitably resulting in a reduced *H coverage during H_2_-SCR reaction^30^. Moreover, these chemical modification strategies were found to be effective only for specific Pt catalyst systems, and in most cases the enhancement was only restricted to NO_*x*_ reduction activity but not to N_2_ selectivity. Therefore, there is urgent need to design a simple, effective and universal strategy to boost the H_2_ activation while reducing the NO adsorption on Pt active sites, thus improving the low-temperature activity and N_2_ selectivity in the H_2_-SCR reaction on Pt-based catalysts accordingly.

Different from the sophisticated chemical modification strategies as previously reported, in this work, we successfully developed a simple, sustainable physical mixing strategy of oxide-supported Pt catalysts (e.g., Pt/TiO_2_, Pt/Al_2_O_3_, or Pt/SiO_2_) with various zeolites (e.g., H-Y, H-ZSM-5, H-chabazite (CHA), H-ferrierite (FER), or H-Beta) to significantly promote the H_2_-SCR reaction. Using this facile approach that is easy to scale up in industry, a universal increase in both the H_2_-SCR activity and N_2_ selectivity was achieved. Focusing on a typical physically mixed catalyst system involving the extensively studied Pt/TiO_2_^31,32^ and commercial H-Y zeolite (i.e., Pt/TiO_2_ + Y), in-depth mechanistic studies were performed through the combined experimental and theoretical approaches. It was clearly revealed that the introduction of Y zeolite facilitated the formation of water-enriched micro-environment on Pt/TiO_2_, which played a crucial role in mitigating the over-strong adsorption of NO while promoting the H_2_ activation on Pt sites. As a result, the disassociation of NO, a crucial step in the H_2_-SCR reaction, was substantially promoted, leading to the drastic enhancement in the catalytic performance.

## Results

### Physical mixing of Pt catalysts and zeolites to promote the H_2_-SCR reaction

**Fig. 1:**
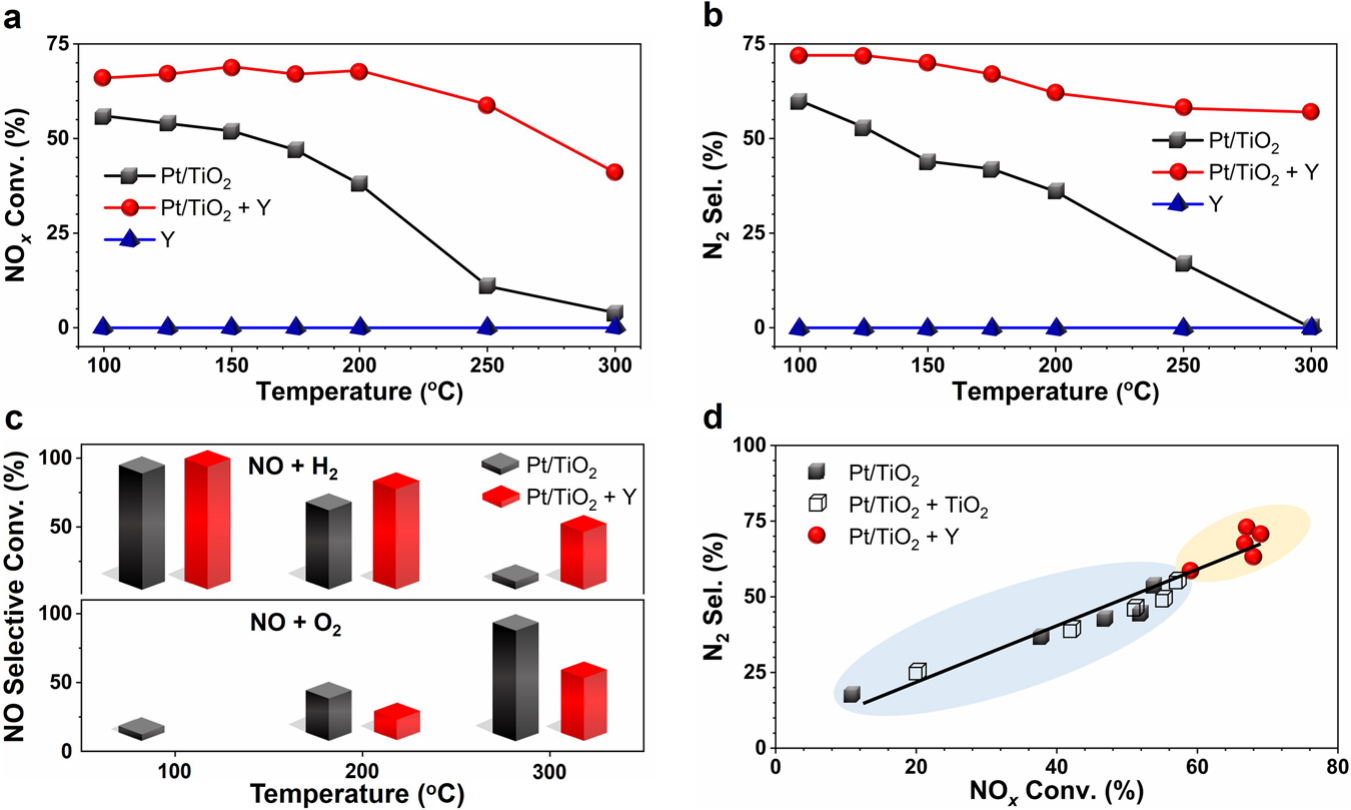
Effect of physical mixing Pt/TiO_2_ with Y zeolite on the H_2_-SCR performance. **a** NO_*x*_ conversion, and (**b**) N_2_ selectivity in H_2_-SCR reaction; **c** NO selective conversion (*i.e*., NO reacting with H_2_ or O_2_) in H_2_-SCR reaction over Pt/TiO_2_ and Pt/TiO_2_ + Y catalysts (see Methods section for detailed calculation); **d** Correlation between N_2_ selectivity and NO_*x*_ conversion in H_2_-SCR reaction over Pt/TiO_2_, Pt/TiO_2_ + TiO_2_, and Pt/TiO_2_ + Y catalysts. Reaction conditions: 26 mg of Pt/TiO_2_ catalyst, or a physical mixture containing 26 mg of Pt/TiO_2_ and 26 mg of Y or TiO_2_; steady-state testing; 500 ppm NO, 1% H_2_, 10% O_2_, 5% CO_2_, and 5% H_2_O; weight hourly space velocity (WHSV) = 461,540 mL·g_Pt/TiO2_^–1^·h^–1^.

The Pt/TiO_2_ catalyst was prepared via a conventional incipient wetness impregnation (IWI) method using colloidal Pt precursor and a commercial TiO_2_ support. In the H_2_-SCR reaction under typical given condition, the Pt/TiO_2_ catalyst showed NO_*x*_ conversion above 11% (Fig. 1a) and N_2_ selectivity above 17% (Fig. 1b) below 250 °C. When physically mixing the Pt/TiO_2_ catalyst with an inactive commercial H-Y zeolite (SiO_2_/Al_2_O_3_ molar ratio = 30) (Fig. 1a, b), within the investigated temperature range, the Pt/TiO_2_ + Y catalyst system showed substantially improved catalytic performance, with NO_*x*_ conversion above 59% and N_2_ selectivity above 58% below 250 °C. In addition, this Pt/TiO_2_ + Y catalyst showed much higher reaction rates and N₂ selectivity at 100 and 200 °C compared to most reported Pt and Pd catalysts (Supplementary Table 1). Such a broad operation temperature window (100–250 °C) and excellent catalytic performance from Pt/TiO_2_ + Y system are highly desirable for the practical H_2_-SCR application^33^. In the presence of both H_2_ and O_2_, NO can either be reduced by H_2_ to form N_2_/N_2_O or be oxidized by O_2_ to form NO_2_ (Supplementary Fig. 1). Therefore, it is reasonable that the NO_*x*_ conversion and N_2_ selectivity could hardly achieve 100% under the high space velocity H_2_-SCR testing conditions with H_2_O and CO_2_ (500 ppm NO, 1% H_2_, 10% O_2_, 5% CO_2_, and 5% H_2_O; WHSV = 461,540 mL·g_Pt/TiO2_^–1^·h^–1^). Comparing to Pt/TiO_2_, the Pt/TiO_2_ + Y system consistently showed higher selectivity towards NO reduction and lower selectivity towards NO oxidation during the H_2_-SCR reaction (Fig. 1c), particularly at high temperatures. The results clearly demonstrated that the presence of Y significantly promoted the NO reduction by H_2_ on Pt/TiO_2_ + Y system.

To verify if there was synergy effect and how it worked between Pt/TiO_2_ and Y components, we investigated the different physical mixing methods (Supplementary Fig. 2a) and see how the H_2_-SCR performance was impacted. It was demonstrated that, in clear contrast to the similar catalytic performance (i.e., low NO_*x*_ conversion and low N_2_ selectivity) obtained on Pt/TiO_2_ + Y-front and Pt/TiO_2_ + Y-rear systems, much more excellent H_2_-SCR performance was achieved on the Pt/TiO_2_ + Y system, where Pt/TiO_2_ and Y powders were thoroughly physically mixed with appropriate contact (Supplementary Fig. 2b, c). To achieve even closer contact between Pt/TiO_2_ and Y, we further physically mixed the Pt/TiO_2_ and Y powders in the presence of water, referred as (Pt/TiO_2_ + Y)_H_2_O, and loaded Pt onto a pre-prepared 50% TiO_2_/Y support (denoted as Pt/TiO_2_/Y). It was observed that the (Pt/TiO_2_ + Y)_H_2_O catalyst showed slightly higher activity (Supplementary Fig. 3), and the Pt/TiO_2_/Y catalyst exhibited lower activity compared to the Pt/TiO_2_ + Y catalyst. However, both catalysts demonstrated lower N_2_ selectivity than Pt/TiO_2_ + Y catalyst. These results evidently suggest the critical role of establishing an appropriate contact between Pt/TiO_2_ and Y zeolite in enhancing the overall H_2_-SCR performance. As shown in Supplementary Fig. 4, the optimal content of Y in the Pt/TiO_2_ + Y mixture system was determined as 50 wt%, and this formulation was simply denoted as Pt/TiO_2_ + Y thereafter. To better understand this system, we also physically mixed the Pt/TiO_2_ catalyst with additional TiO_2_, and the Pt/Y catalyst (prepared by IWI method) with additional TiO_2_ or Y, and used them as reference catalysts. As presented in Supplementary Fig. 5, the physical mixing of Pt/TiO_2_ and TiO_2_ showed no obvious impact on the NO_*x*_ conversion and N_2_ selectivity. However, the physical mixing of Pt/Y with TiO_2_ or Y resulted in considerable enhancement of the H_2_-SCR performance. It was worth noting that the Pt/TiO_2_ + Y formulation outperformed all other catalysts in terms of H_2_-SCR activity and showed reasonable N_2_ selectivity. To gain a deeper insight into the Y promotion effect, the relationship between N_2_ selectivity and NO_*x*_ conversion in the H_2_-SCR reaction on selected catalysts was established, as depicted in Fig. 1d. Interestingly, the N_2_ selectivity versus NO_*x*_ conversion on all catalysts adhered to the same linear relationship, suggesting that the addition of Y or TiO_2_ did not alter the overall H_2_-SCR reaction mechanism on Pt/TiO_2_ catalyst (yet the Y addition might have changed the N_2_ formation pathway leading to lower N_2_O production, which can be verified by the subsequent experimental results and theoretical calculations).

In addition to Y, the use of other types of zeolites for physical mixing with Pt/TiO_2_ has also been explored in the H_2_-SCR reaction (Supplementary Fig. 6). Remarkably, the incorporation of different zeolites such as ZSM-5, CHA, FER, and Beta also yielded substantial benefit, significantly enhancing the H_2_-SCR performance. Considering both the H_2_-SCR activity and N_2_ selectivity in the investigated temperature range, it is evident that Y stands out as the optimal zeolite for promoting the Pt/TiO_2_ catalyst. To simulate the status of catalysts for H_2_-ICE exhaust purification after prolonged operation, hydrothermal aging on Pt/TiO_2_ and Pt/TiO_2_ + Y catalysts was conducted at 650 °C for 50 h under 10% H_2_O and 10% O_2_. As shown in Supplementary Fig. 7, not only before but also after the hydrothermal aging, the inclusion of Y in Pt/TiO_2_ + Y system consistently exhibited remarkable enhancement on the H_2_-SCR performance, with notably higher NO_*x*_ conversion and N_2_ selectivity achieved than those by the zeolite-free Pt/TiO_2_ catalyst. To further verify the universality of this physical mixing strategy, the H_2_-SCR testing on the hydrothermally aged Pt/Al_2_O_3_ and Pt/SiO_2_ catalysts with and without Y addition was also performed, and the results are shown in Supplementary Fig. 8. Evidently, the aged Pt/Al_2_O_3_ + Y and Pt/SiO_2_ + Y systems demonstrated significantly enhanced activity and N_2_ selectivity across the entire spectrum of reaction temperatures when contrasted with their Y-absent counterparts. It is clear that physically mixing the conventional Pt/oxide catalysts with zeolites represents a simple yet universally effective strategy for boosting the H_2_-SCR performance, particularly tailored for the efficient NO_*x*_ removal from vehicle exhaust at low temperatures.

### Structural characterization of Pt/TiO_2_ before and after physical mixing with Y zeolite

It might be expected that the physical mixing with Y could have modified the physicochemical properties of Pt/TiO_2_ leading to the distinguishable catalytic performance. We excluded this hypothesis by systematically characterizing the Pt/TiO_2_ and Pt/TiO_2_ + Y catalysts using multiple techniques. X-ray diffraction (XRD) (Supplementary Fig. 9) and N_2_ adsorption-desorption experiments (Supplementary Fig. 10 and Supplementary Table 2) revealed that the physical mixing showed negligible impact on the crystal structure and textual properties including surface area and porosity of both Pt/TiO_2_ and Y. It was observed that the Pt/TiO_2_ + Y system exhibited a similar TiO_2_ grain size (20.6 nm) to that of Pt/TiO_2_ (20.0 nm), and its surface area (349 m^2^/g) and total pore volume (0.318 cm^3^/g) were approximately the mathematical average of the values for Pt/TiO_2_ (81 m^2^/g, 0.178 cm^3^/g) and Y (709 m^2^/g, 0.513 cm^3^/g), respectively. Additionally, the Pt/TiO_2_ + Y system demonstrated structural stability, with no apparent changes in crystal structure or textural properties after reaction at 300 °C under testing conditions with H_2_O. In addition to the presence of micropores with the average diameter of 0.6 nm, Y zeolite also displayed significant mesopore defects with the average diameter of 3.8 nm that were probably formed during the dealumination process for Y zeolite production. These defects could potentially offer a substantial number of special Brønsted acidic sites (i.e., hydroxyls associated to extra-framework Al enriched on the inner pore surface), which might play a crucial role in facilitating the adsorption of H_2_O molecules to occupy the mesopore structures^34^. The change of the H_2_O adsorption behavior induced by Y zeolite might have altered the H_2_-SCR reaction pathway on Pt/TiO_2_, which will be thoroughly discussed in later sections.

**Fig. 2:**
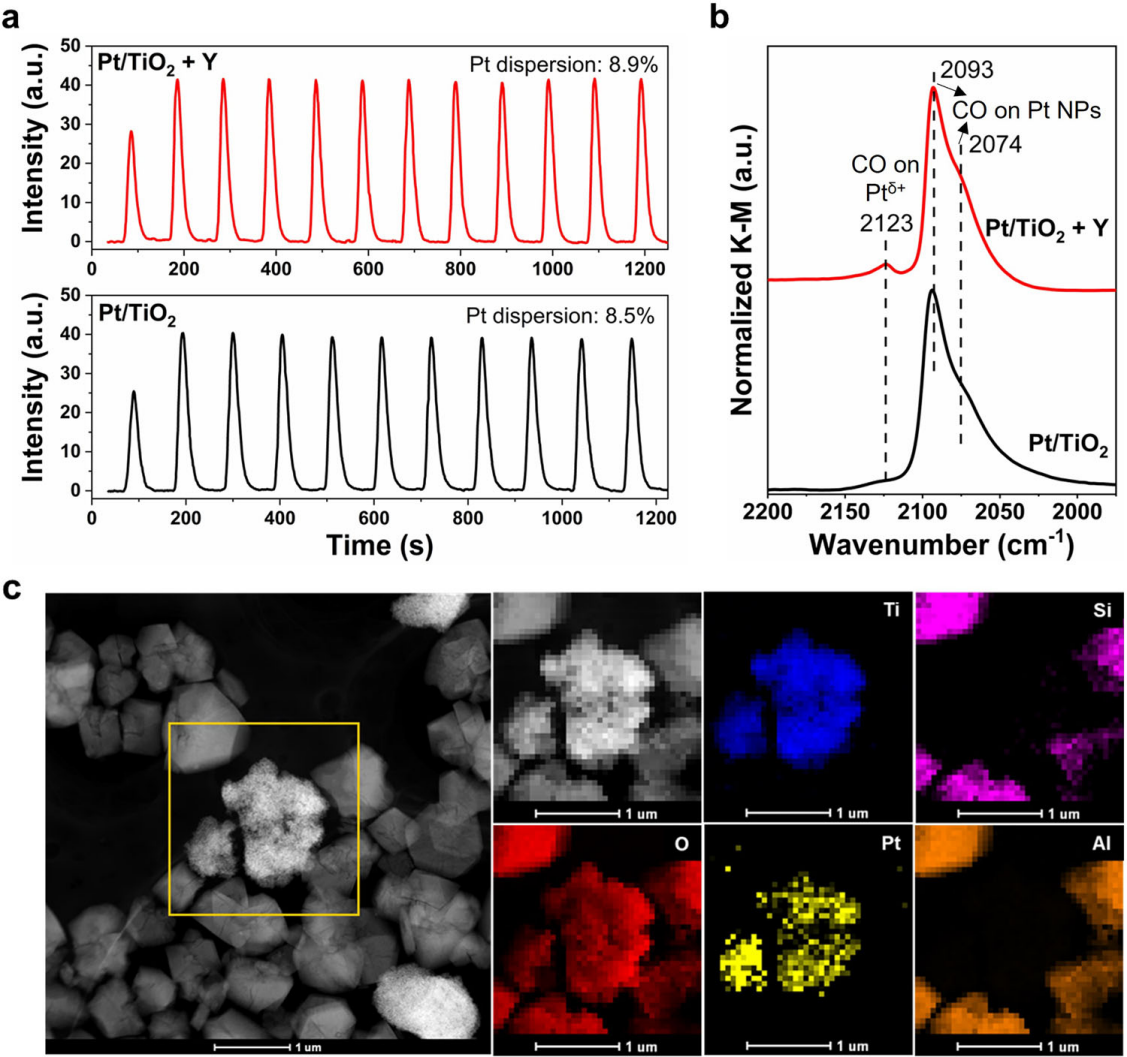
Structural characterization of Pt/TiO_2_ + Y. **a** CO pulse titration results (with the Pt metal dispersion data inserted); **b** in situ DRIFTS of CO adsorption at 25 °C on Pt/TiO_2_ and Pt/TiO_2_ + Y catalysts; **c** EDS mapping images for Pt/TiO_2_ + Y system.

As expected, the Pt particles within both Pt/TiO_2_ and Pt/TiO_2_ + Y catalysts showed very similar average sizes (6.0 nm vs. 6.2 nm), Pt dispersions (8.9% vs. 8.5%), CO adsorption features on Pt particle, and Pt-Pt coordination numbers (11.4 vs. 10.7), as evidenced by the characterization results of transmission electron microscopy (TEM), CO pulse titration, in situ diffuse reflectance infrared Fourier transform spectroscopy (DRIFTS) of CO adsorption, and X-ray absorption spectroscopy (XAS) (Fig. 2a, b, Supplementary Figs. 11, 12 and 13, Supplementary Table 3). Furthermore, the linear combination fitting results of X-ray absorption near-edge structure (XANES) for Pt L_3_-edge demonstrated that the averaged oxidation states of Pt were 0.21 and 0.48 in Pt/TiO_2_ before and after the Y addition. These values closely resembled the metallic Pt, a finding further supported by the X-ray photoelectron spectroscopy (XPS) analysis of Pt 4*d* (Supplementary Fig. 14, Supplementary Table 4). These results clearly demonstrated that the physical mixing with Y zeolite did not change the structure of Pt/TiO_2_, and this conclusion was further supported by the observation of almost identical H_2_ temperature-programed reduction (H_2_-TPR) profiles on Pt/TiO_2_ and Pt/TiO_2_ + Y (Supplementary Fig. 15). Additionally, the energy dispersive spectroscopy (EDS) mapping results of Pt/TiO_2_ + Y revealed that the Pt/TiO_2_ components were surrounded by Y zeolite particles, without obvious direct interaction between Pt species and Y, before and after H_2_-SCR reaction (Fig. 2c, Supplementary Fig. 16). Therefore, different from the chemical modifications as reported previously^24–29^, the substantial enhancement in H_2_-SCR performance on Pt/TiO_2_ by physically mixing with Y was unequivocally attributable to the factors other than the active site modification.

### Understanding on the Y promotion effect in Pt/TiO_2_ + Y system

To determine the role of each reactant and the promotion effect of Y, the reaction orders of NO, H_2_ and O_2_ were measured for the H_2_-SCR reaction. It was found that the reaction orders of NO on both Pt/TiO_2_ and Pt/TiO_2_ + Y were dependent on the NO partial pressure (Supplementary Fig. 17a). On Pt/TiO_2_, at NO partial pressure below 25.3 Pa, the NO reaction order was determined as 0.95, while this value decreased to 0.63 at NO partial pressure above 25.3 Pa. Meanwhile, the reaction orders of H_2_ and O_2_ on Pt/TiO_2_ were determined as 0.48 and –0.13 (Supplementary Fig. 17b, c), respectively. After physical mixing with Y, there was no evident change in the O_2_ reaction order on Pt/TiO_2_ + Y (only from –0.13 to –0.08). However, a notable increase in the NO reaction order (from 0.95 to 1.09 at lower NO partial pressure, and from 0.63 to 0.80 at higher NO partial pressure) and an obvious decrease in the H_2_ reaction order (from 0.48 to 0.32) were observed on Pt/TiO_2_ + Y. These results suggest that the introduction of Y probably decreased the NO adsorption, and at the same time promoted the H_2_ activation because of the potential increase in H_2_ coverage on catalyst surface. The enhanced H_2_ activation was further supported by the experimental findings presented in Supplementary Figs. 17d and 18. The Pt/TiO₂ + Y system demonstrated superior H₂ oxidation activity compared to the Pt/TiO₂ reference, with the H₂ oxidation activity promoted further as the Y content in the Pt/TiO₂ + Y system increased (Supplementary Fig. 18). This additional increase in H₂ activation could improve the low-temperature activity and reduce the high-temperature activity, as observed on Pt/TiO₂ + Y-67% compared to that on Pt/TiO₂ + Y-50% (Supplementary Fig. 4)^14,25^. Under our testing conditions (500 ppm NO, 1% H_2_, and 10% O_2_), the H_2_-SCR reaction rates can be expressed as: *r*_(Pt/TiO2)_ = k_1_⋅[NO]^0.63^⋅[H_2_]^0.48^⋅[O_2_]^−0.13^ and *r*_(Pt/TiO2 +Y)_ = k_2_⋅[NO]^0.80^⋅[H_2_]^0.32^⋅[O_2_]^−0.08^, where k_1_ and k_2_ are constants. Notably, the NO and H₂ reaction orders on both Pt/TiO₂ (0.63 and 0.48, respectively) and Pt/TiO₂ + Y (0.80 and 0.32, respectively) are lower than 1. This suggests that the H₂-SCR reaction on both catalysts involved adsorbed NO and dissociated H* species, following the Langmuir-Hinshelwood (L-H) mechanism. Without changing the L-H mechanism, the enhanced H_2_ activation could contribute to the improved H_2_-SCR activity of Pt/TiO₂ + Y.

The effect of the possibly present NO_2_ or NH_3_ in the reaction atmosphere on H_2_-SCR activity was also studied. In separate NO oxidation testing, the Pt/TiO_2_ + Y system displayed noticeably lower NO oxidation activity comparing to Pt/TiO_2_ (Supplementary Fig. 19a), and the presence of NO_2_ in the H_2_-SCR reaction atmosphere drastically decreased the low-temperature NO_*x*_ conversion on both Pt/TiO_2_ and Pt/TiO_2_ + Y (Supplementary Fig. 19b). Therefore, the presence of any NO_2_, generated during H_2_-SCR, was not responsible for the enhanced H_2_-SCR activity on Pt/TiO_2_ + Y. In addition, the potential promotional effect of NH_3_ (possibly formed in situ through the reduction of NO_*x*_ by H_2_) on H_2_-SCR activity was ruled out on both Pt/TiO_2_ and Pt/TiO_2_ + Y. This was evident from the decrease in the H_2_-SCR activity observed upon the introduction of NH_3_ into the reaction stream at different temperatures (Supplementary Fig. 20).

**Fig. 3:**
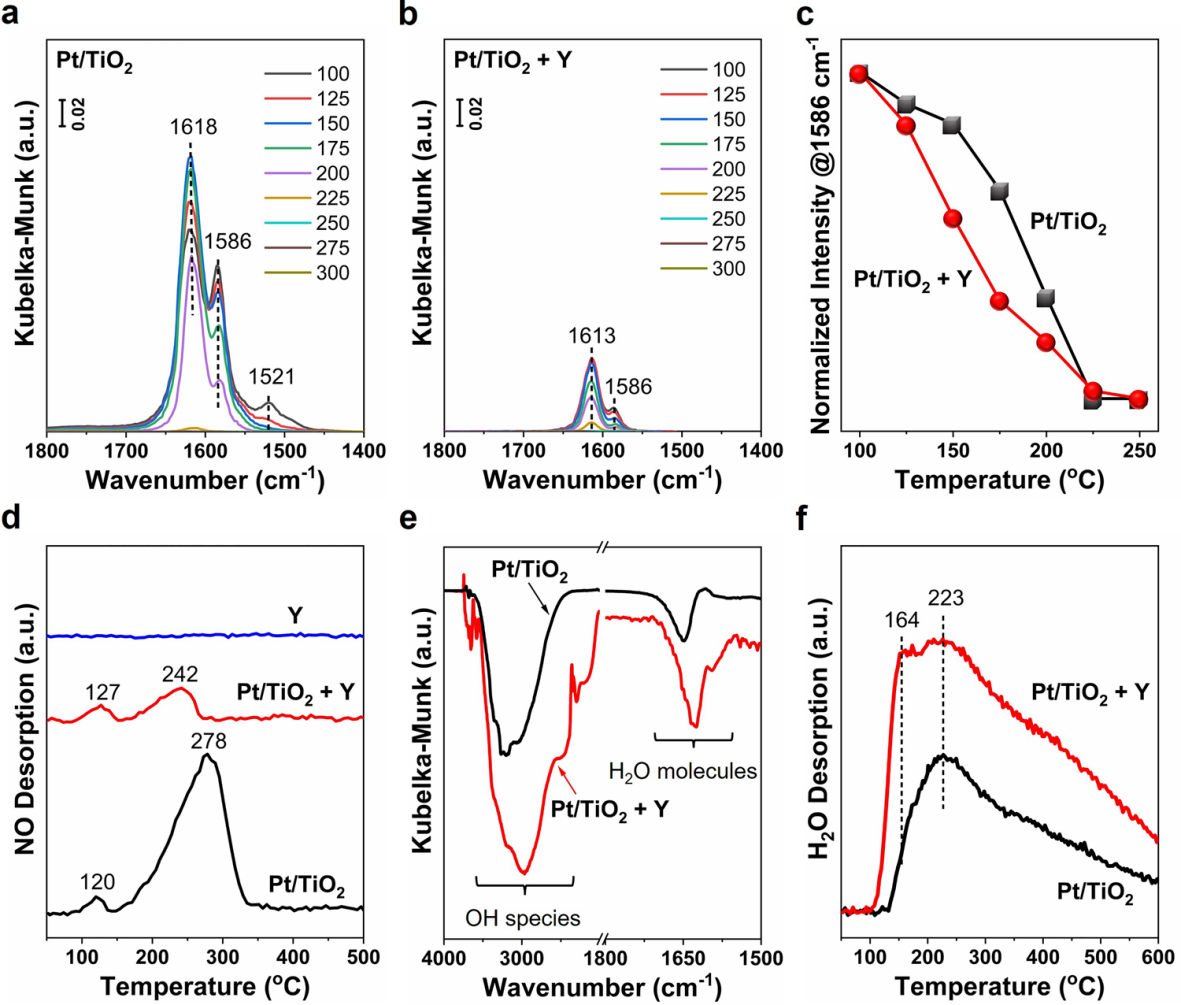
Effects of Y addition on NO and H_2_O adsorption properties. In situ DRIFTS of NO desorption on (**a**) Pt/TiO_2_ and (**b**) Pt/TiO_2_ + Y catalysts, and (**c**) normalized peak intensity (at 1586 cm^–1^) for NO adsorption on Pt/TiO_2_ and Pt/TiO_2_ + Y catalysts at different temperatures; (**d**) NO-TPD profiles, (**e**) in situ DRIFTS of H_2_O adsorption at 120 ^o^C, and (**f**) H_2_O-TPD profiles on Pt/TiO_2_ and Pt/TiO_2_ + Y catalysts.

To understand the impact of Y addition on NO adsorption behavior, the in situ DRIFTS of NO desorption at different temperatures and NO-temperature programmed desorption (NO-TPD) were conducted on Pt/TiO_2_ and Pt/TiO_2_ + Y catalysts. As shown in Fig. 3a, the NO adsorption on Pt/TiO_2_ at 100 °C showed three distinctive bands, corresponding to bridging nitrates (1618 cm^−1^), bidentate nitrates (1586 cm^−1^), and monodentate nitrates (1521 cm^−1^)^35,36^. In clear contrast, the NO adsorption on Pt/TiO_2_ + Y exhibited a significantly lower intensity, with the disappearance of monodentate nitrates (Fig. 3b). As the temperature elevated, the nitrate species on both Pt/TiO_2_ and Pt/TiO_2_ + Y catalysts decreased in intensity, following the sequence of monodentate nitrates > bidentate nitrates > bridging nitrates (Fig. 3a and b). An initial upswing in the bridging nitrates on Pt/TiO_2_ was noted, attributed to the intrinsic transformation within the different types of nitrate species^37^. To assess the NO adsorption affinity, the normalized intensities of bidentate nitrates were presented at different temperatures (Fig. 3c). A much more rapid nitrate desorption from Pt/TiO_2_ + Y was observed comparing to that from Pt/TiO_2_. Such results unequivocally demonstrated the substantial inhibitory effect of Y zeolite on the NO adsorption onto Pt/TiO_2_, concurrently fostering the desorption of NO from Pt/TiO_2_ + Y. These findings were further supported by the NO-TPD results (Fig. 3d), revealing that the Pt/TiO_2_ + Y system indeed exhibited notably reduced NO desorption intensity and lowered desorption temperature (242 ^o^C) comparing to Pt/TiO_2_ (278 ^o^C).

Considering that H_2_O is the primary product in the H_2_-SCR reaction, the impact of Y addition on H_2_O adsorption property was also investigated. The in situ DRIFTS of H_2_O adsorption on both Pt/TiO_2_ and Pt/TiO_2_ + Y at 120 ^o^C clearly showed distinct peaks at *ca*. 1630 cm^−1^, indicative of adsorbed H_2_O molecules^38^. Additionally, broad peaks at *ca.* 3200 cm^−1^ were observed, corresponding to the hydroxyl species derived from adsorbed H_2_O with bending feature^38–40^. Comparing to the case on Pt/TiO_2_, H_2_O adsorption on Pt/TiO_2_ + Y displayed more prominent peaks (Fig. 3e), suggesting the enhanced H_2_O adsorption due to the presence of Y. This enhancement was also confirmed by the H_2_O-TPD results (Fig. 3f), where more pronounced H_2_O desorption peaks were observed on Pt/TiO_2_ + Y. Other than the H_2_O desorption peak observed at 223 ^o^C on both catalysts, an additional desorption peak at 164 ^o^C was detected only on Pt/TiO_2_ + Y. This low-temperature peak could be attributed to the physically adsorbed H_2_O on the Y zeolite.

**Fig. 4:**
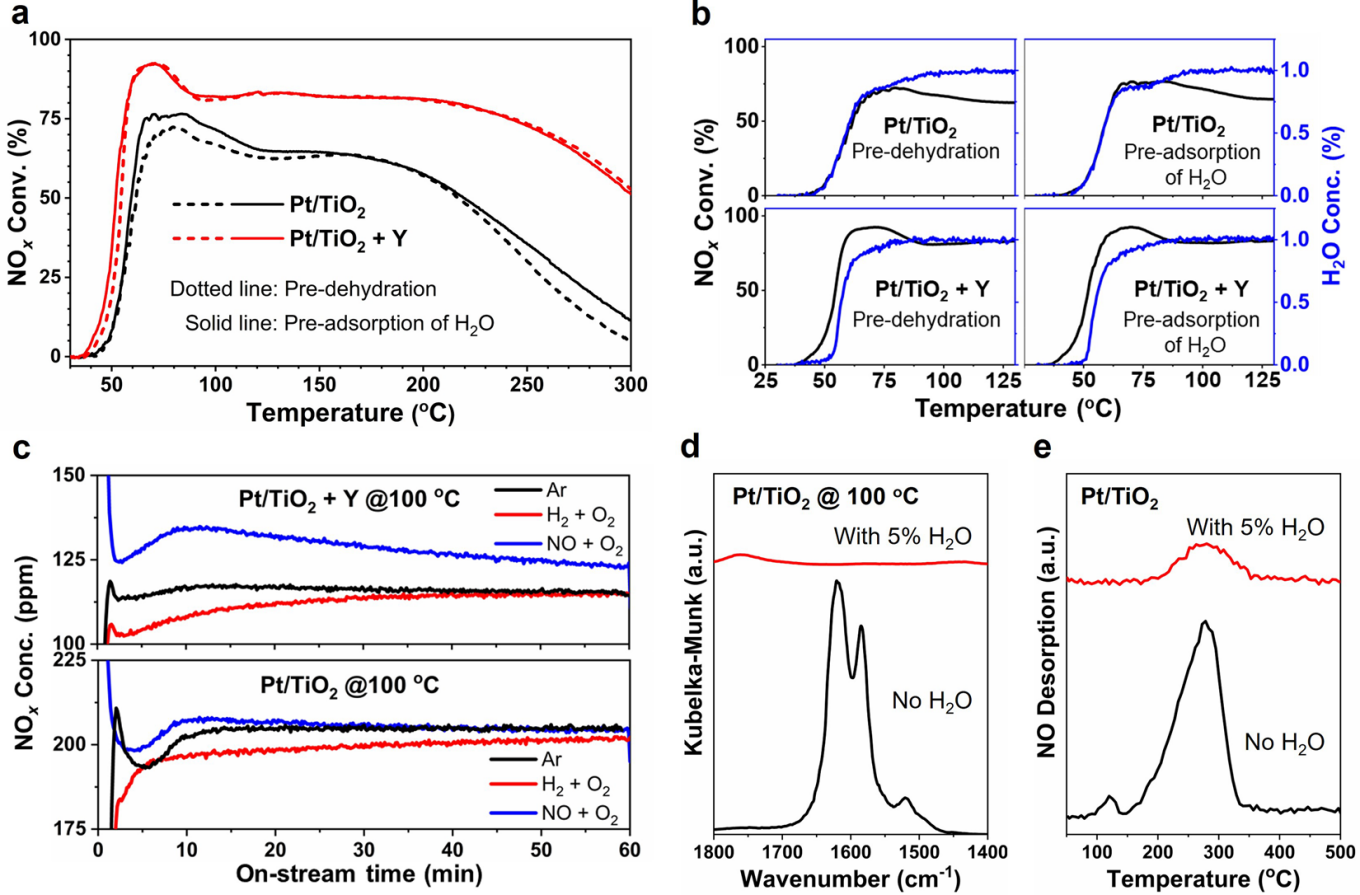
Effects of H_2_O on the H_2_-SCR performance and NO adsorption property. **a** NO_*x*_ conversion and (**b**) gas phase H_2_O formation during the H_2_-SCR reaction over Pt/TiO_2_ and Pt/TiO_2_ + Y catalysts with pre-dehydration at 300 ^o^C or pre-adsorption of H_2_O at 30 ^o^C. Reaction conditions: 26 mg of Pt/TiO_2_ catalyst, or a mixture containing 26 mg of Pt/TiO_2_ and 26 mg of Y; transient-state light-off testing; 500 ppm NO, 1% H_2_, and 10% O_2_; WHSV = 461,540 mL·g_Pt/TiO2_^–1^·h^–1^. **c** Time-resolved NO_*x*_ concentration after switching from different flows (Ar; or 1% H_2_ + 10% O_2_; or 500 ppm NO + 10% O_2_) to H_2_-SCR flow (500 ppm NO + 1% H_2_ + 10% O_2_) on Pt/TiO_2_ and Pt/TiO_2_ + Y catalysts at 100 ^o^C. Testing conditions: 10 mg of Pt/TiO_2_, or a mixture containing 10 mg of Pt/TiO_2_ and 10 mg of Y; WHSV = 1,200,000 mL·g_Pt/TiO2_^–1^·h^–1^. **d** In situ DRIFTS of NO adsorption at 100 ^o^C, and (**e**) NO-TPD profiles on Pt/TiO_2_ catalyst under the NO adsorption conditions with and without 5% H_2_O.

To reveal the effect of H_2_O adsorption on the H_2_-SCR performance, the catalysts were either pre-dehydrated or pre-adsorbed with H_2_O prior to the H_2_-SCR testing. Under the testing condition without H_2_O, Pt/TiO_2_ + Y always outperformed Pt/TiO_2_ in terms of NO_*x*_ conversion and N_2_ selectivity (Fig. 4a, Supplementary Fig. 21a). Comparing to the situation with pre-dehydration, interestingly, the pre-adsorption of H_2_O on both catalysts improved their H_2_-SCR performance. This improvement was particularly significant regarding the NO_*x*_ conversion on Pt/TiO_2_ catalyst, which exhibited relatively weaker H_2_O adsorption capacity as confirmed earlier. The gas phase H_2_O formation was monitored during the H_2_-SCR reaction (Supplementary Fig. 21b). As expected, much faster increase in H_2_O concentration was observed over both catalysts subjected to the pre-adsorption of H_2_O comparing to those subjected to the pre-dehydration. It was noticeable that, as shown in Fig. 4b, a discernible correlation emerged between the elevation in NO_*x*_ conversion and the concurrent rise in gas phase H_2_O concentration over Pt/TiO_2_ catalyst. However, over Pt/TiO_2_ + Y system, the rise in gas phase H_2_O concentration exhibited a delay compared to the progression of NO_*x*_ conversion, suggesting the capture of in situ formed H_2_O due to the presence of Y. To further confirm the promotion effect of in situ generated H_2_O and to verify the effect of NO adsorption on the H_2_-SCR activity, transient H_2_-SCR testing was conducted at 100 ^o^C (Fig. 4c). Using the NO_*x*_ concentrations when switching from Ar flow to H_2_-SCR flow as baselines, significant decrease in NO_*x*_ concentrations was observed when switching from H_2_ + O_2_ flow to H_2_-SCR flow, while obvious increase in NO_*x*_ concentrations was observed when switching from NO + O_2_ flow to H_2_-SCR flow, on both catalysts. Clearly, initiating a pre-flow of H_2_ + O_2_ yielded benefit on improving the H_2_-SCR activity, while pre-flowing NO + O_2_ inhibited the H_2_-SCR reaction to a certain extent. Such inhibition caused by the NO + O_2_ flow could be due to the extensive coverage of Pt sites by NO, impeding the activation of H_2_^30^. At 100 ^o^C, the complete oxidation of H_2_ to H_2_O could be achieved on both catalysts (Supplementary Fig. 18). Consequently, the benefit of pre-flowing H_2_ + O_2_ should be originated from the adsorption of in situ formed H_2_O. The presence of H_2_O could strongly inhibit the NO adsorption, as confirmed by the in situ DRIFTS (Fig. 4d) and NO-TPD (Fig. 4e) analyses conducted on Pt/TiO_2_ catalyst. The physical mixing of Pt/TiO_2_ with Y could further promote the adsorption of in situ generated H_2_O (Fig. 3e, f), creating a H_2_O-rich environment around the Pt sites and facilitating the formation of a H_2_O-covered Pt surface. This surface could reduce the NO coverage on Pt sites, thereby improving H₂ activation and H₂-SCR performance (Fig. 4a). However, introducing 5% external H_2_O into the reaction flow could significantly inhibit the diffusion of NO and H_2_ (NO/H_2_/H_2_O molar ratio = 1/20/100) to the catalyst surface. Despite this inhibition resulting in the decreased activity for both catalysts, the Pt/TiO₂ + Y catalyst still exhibited significantly higher activity compared to the Pt/TiO₂ catalyst (Fig. 1a).

To elucidate the intrinsic promotion effect of Y addition to Pt/TiO_2_ on the H_2_-SCR performance, systematic density functional theory (DFT) calculations were performed. The Pt (111) surface was selected to represent the Pt active site in Pt/TiO_2_ catalyst due to its high thermodynamic stability (Supplementary Fig. 22). As previously confirmed, the Y zeolite in Pt/TiO_2_ + Y system possessed high ability to capture the in situ generated H_2_O, creating the H_2_O-rich environment around Pt sites. In light of this, a stable H_2_O/Pt (111) interface was constructed (Supplementary Fig. 23), featuring a hydrogen bonding network with half of H_2_O molecules dissociated on Pt with 2/3 monolayer (ML) coverage^41,42^. Such configuration was denoted as the H_2_O/Pt (111) surface to represent the Pt active site in Pt/TiO_2_ + Y system.

**Fig. 5:**
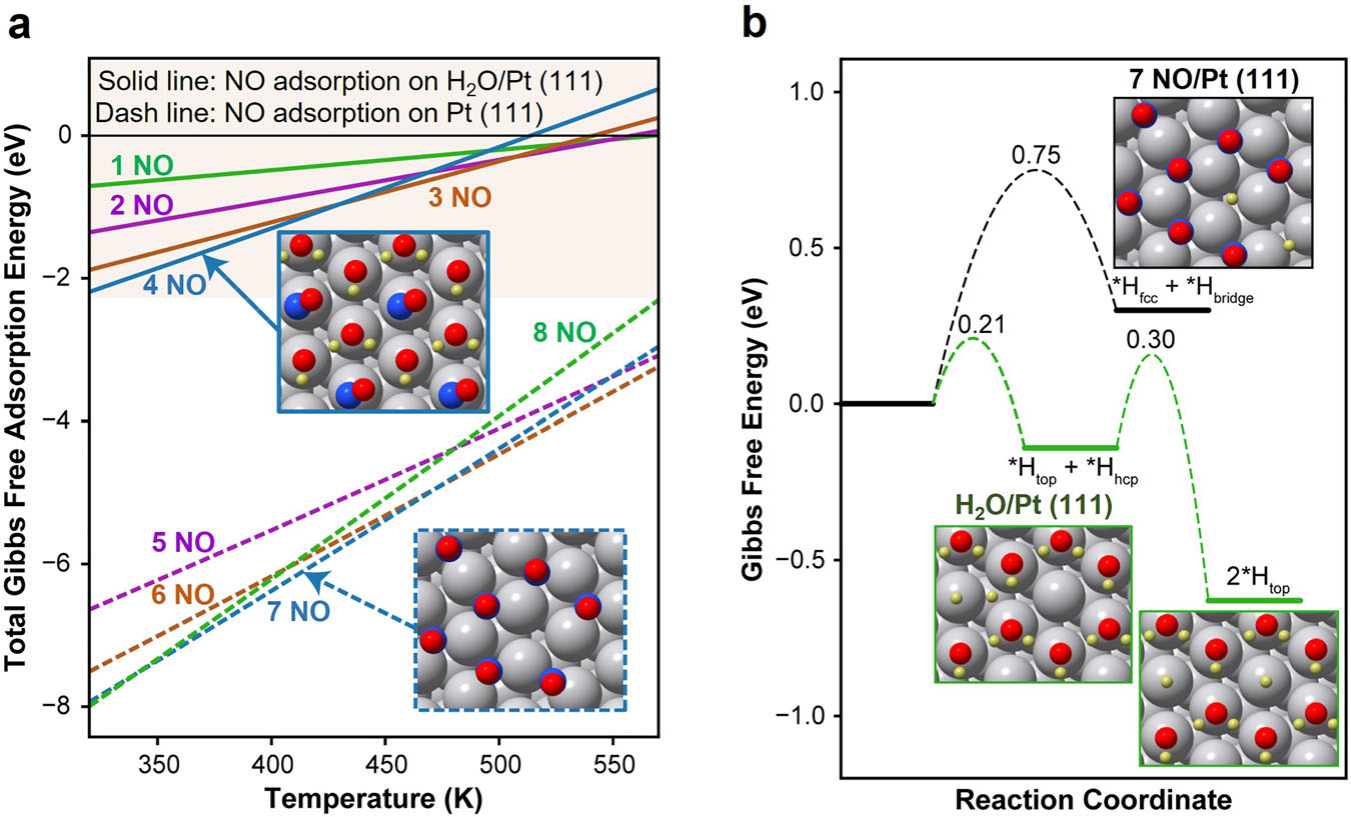
NO adsorption and H_2_ activation on Pt (111) and H_2_O/Pt (111) surfaces. **a** Total Gibbs free adsorption energy of NO molecules on Pt (111) and H_2_O/Pt (111) surfaces. The reference state is the gas phase NO at 50 Pa. **b** Gibbs free energy diagram of H_2_ activation on the 7 NO/Pt (111) surface and the H_2_O/Pt (111) surface. The reference state is the gas phase H_2_ at 1 atm. Color code: Pt (silver), O (red), N (blue), and H (yellow).

As shown in Supplementary Fig. 24, the Pt (111) surface was found more favorable for the NO adsorption with much higher free adsorption energy (–1.77 eV) comparing to that for H_2_ adsorption (–0.87 eV) at the low coverage limit. Consequently, the optimal NO coverage on Pt (111) was firstly investigated by calculating the total Gibbs free adsorption energies, which was determined as 7/12 ML at relative low temperatures (T = 320-470 K and *P*_NO_ = 50 Pa) and in line with previous study^30^. With the highest total Gibbs free adsorption energy, the 7 NO/Pt (111) structure emerged as the most stable configuration (Fig. 5a, Supplementary Fig. 25), which was adopted as the starting point for studying the H_2_ activation and reaction mechanism on Pt/TiO_2_. On the stable H_2_O/Pt (111) surface, the presence of a repulsive hydrogen bonding network led to a significant decline in the averaged free NO adsorption energies. Consequently, a notably reduced NO coverage (1/3 ML) was observed on the H_2_O/Pt (111) surface (Fig. 5a, Supplementary Fig. 26), in comparison to the NO overage (7/12 ML) on the Pt (111) surface. Considering that the weakly-bonded *NO (T = 373 K, average *G*_ads_ = ~0.5 eV) was highly active and unstable, the H_2_O/Pt (111) surface without *NO was used as the starting point for studying the H_2_ activation and reaction mechanism on Pt/TiO_2_ + Y. Accordingly, the H_2_ adsorption and activation energies on 7 NO/Pt (111) and H_2_O/Pt (111) surfaces were calculated, and the results are shown in Fig. 5b. Comparing to the endergonic process of H_2_ adsorption (Δ*G* = 0.30 eV) and high H_2_ activation barrier (*G*_a_ = 0.75 eV) observed on 7 NO/Pt (111) surface, an exergonic process of H_2_ adsorption (Δ*G* = –0.63 eV) and much lower H_2_ activation barrier (*G*_a_ = 0.30 eV) was found on H_2_O/Pt (111) surface. Evidently, the H_2_O/Pt (111) surface benefited the H_2_ adsorption and activation, well aligned with the experimental results showing that Pt/TiO_2_ + Y system exhibited superior H_2_ activation ability comparing to Pt/TiO_2_ (Supplementary Fig. 18).

**Fig. 6:**
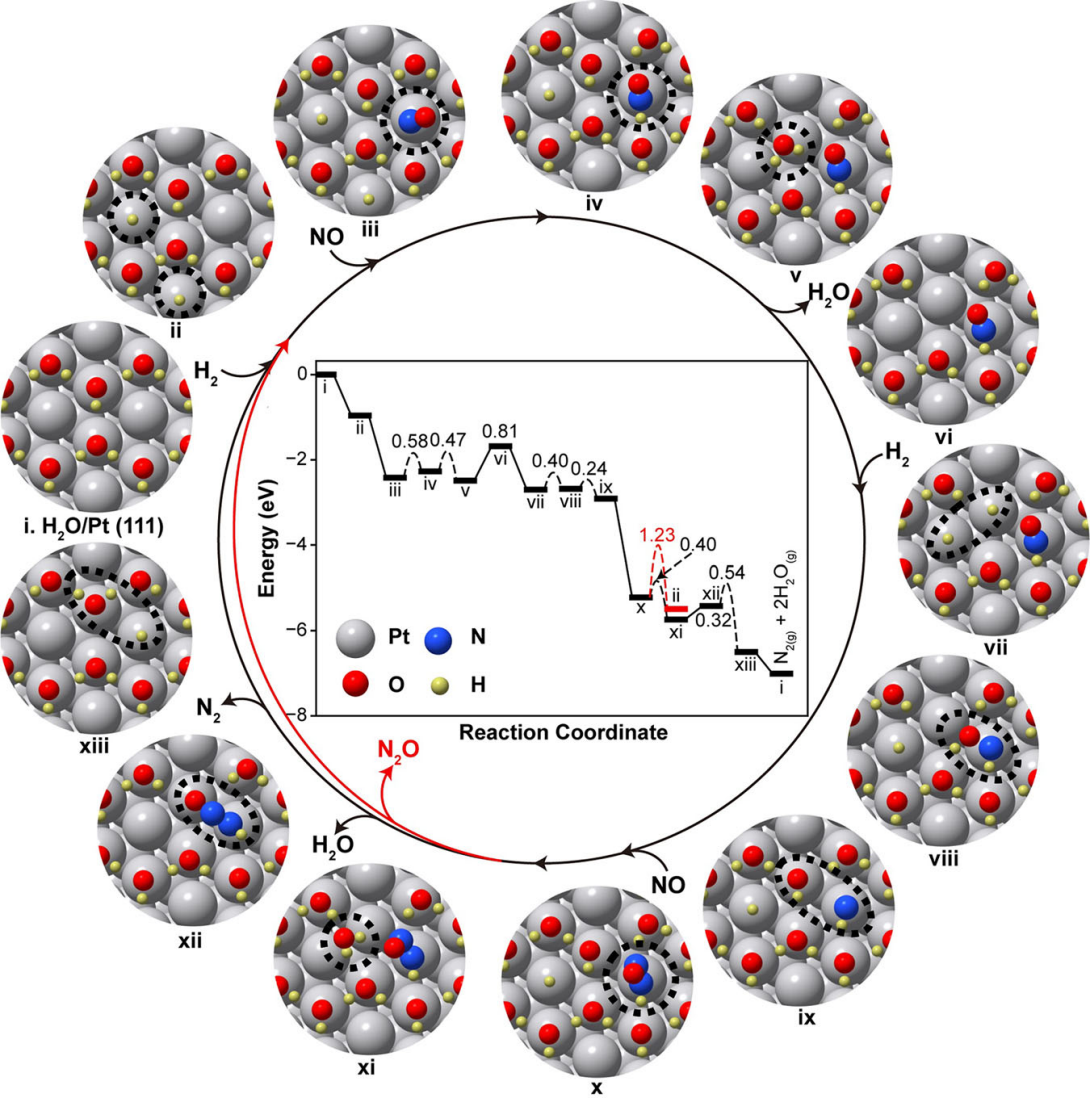
Potential energy diagrams and configurations for the H_2_-SCR cycle on the Pt/TiO_2_ + Y catalyst. The reaction was proposed to proceed on the H_2_O/Pt (111) surface representing the structure of Pt/TiO_2_ + Y catalyst under reaction conditions. The reaction energies and activation energies are indicated in eV in the diagram. Color code: Pt (silver), O (red), N (blue), and H (yellow). Corresponding energies are given in Supplementary Table 6.

The theoretical calculations of H_2_-SCR reaction mechanism on Pt (111) and H_2_O/Pt (111) surfaces were conducted to further elucidate the promotion effect of Y zeolite in Pt/TiO_2_ + Y system. On Pt (111) surface, as shown in Supplementary Fig. 27 and Supplementary Table 5, the dissociation of *HNOH species into *NH and *OH was found to be the rate-determining step (RDS) for NO reduction with an activation energy (*E*_a_) of 1.20 eV. Due to the high NO coverage, the inhibited H_2_ activation further hindered the selective reduction of N-containing species to N_2_, resulting in the high N_2_O formation and low N_2_ selectivity. In clear contrast, on H_2_O/Pt (111) surface (Fig. 6, Supplementary Table 6), the *HNOH species could be readily dissociated into *NH and *OH with a lower activation energy of 0.24 eV (image viii to ix). Once the *NH species was formed, the gas phase NO could facilely couple with it to generate *HNNO, involving a substantial exothermicity of 2.31 eV (image ix to x). Interestingly, rather than releasing N_2_O (with *E*_a_ = 1.23 eV from image x to ii), the *HNNO species remained until an OH vacancy was facilely created in the hydrogen bonding network following the H_2_O formation (*E*_a_ = 0.40 eV from image x to xi) and desorption (Δ*E* = 0.32 eV from image xi to xii). Subsequently, the *HNNO species was activated and dissociated, selectively producing N_2_ with a barrier of 0.54 eV (image xii to xiii). Therefore, the RDS for NO reduction on H_2_O/Pt (111) surface included both the creation of OH vacancy in the hydrogen bonding network and N_2_ formation, with an overall activation energy of 0.86 eV (from image xi to xiii). Such activation energy for the RDS of H_2_-SCR reaction on H_2_O/Pt (111) surface was much lower than that on Pt (111) surface (1.20 eV). These simulation results well explained the significant promotion effect of Y zeolite in Pt/TiO_2_ + Y system for H_2_-SCR in terms of both enhanced NO removal efficiency and elevated N_2_ selectivity.

## Discussion

A facile, universal and sustainable strategy of physically mixing Pt/oxide catalysts with zeolites has been successfully developed to improve the H_2_-SCR performance of Pt-based catalysts for low-temperature NO_*x*_ removal. The Pt/TiO_2_ + Y system exhibited superior H_2_-SCR performance consistently in terms of NO_*x*_ conversion and N_2_ selectivity, both before and after hydrothermal aging, as well as across various testing conditions. This catalyst system shows immense potential in H_2_-SCR applications, particularly for H_2_-ICE emission control. It was discovered that the incorporation of Y zeolite effectively promoted H_2_O adsorption and the formation of H_2_O-rich environment surrounding Pt active sites in Pt/TiO_2_ + Y system. This consequently led to the reduction in excessive NO coverage and the improvement in H_2_ activation, yielding substantial advantages for boosting both H_2_-SCR efficiency and N_2_ selectivity. In contrast to modifying the active sites through chemical methods, this study underscores the crucial importance of fine tuning the surrounding environment of active sites through an easy, sustainable physical mixing approach to achieve proficient heterogeneous catalysis.

## Methods

### Catalyst preparation

The Pt/oxide catalysts used in this study, including Pt/TiO_2_, Pt/Al_2_O_3_, and Pt/SiO_2_, were prepared using incipient wetness impregnation (IWI) method. A solution of colloidal Pt (2-6 nm) with 1 wt% Pt was added dropwise onto commercial anatase TiO_2_ (surface area = 90 m^2^/g), γ-Al_2_O_3_ (surface area = 150 m^2^/g), or SiO_2_ (surface area = 180 m^2^/g) under stirring, followed by drying at 120 °C for 1 h. After calcination in air at 550 °C for 2 h with the temperature ramp of 5 ^o^C/min, the catalysts were obtained and denoted as Pt/TiO_2_, Pt/Al_2_O_3_, and Pt/SiO_2_, respectively. As a reference, Pt/Y catalyst was also prepared by the same method using H-Y zeolite (SiO_2_/Al_2_O_3_ molar ratio = 30) as support.

For physical mixing with Pt/oxide catalysts, commercial zeolites including H-Y (SiO_2_/Al_2_O_3_ molar ratio = 30), H-ZSM-5 (SiO_2_/Al_2_O_3_ molar ratio = 30), H-chabazite (CHA, SiO_2_/Al_2_O_3_ molar ratio = 29), H-ferrierite (FER, SiO_2_/Al_2_O_3_ molar ratio = 30), and H-Beta (SiO_2_/Al_2_O_3_ molar ratio = 25) were used. TiO_2_ or H-Y was also used to dilute Pt/TiO_2_ or Pt/Y, respectively, for comparison. The content of additional zeolite/oxide was typically controlled at 50 wt% in the physically mixed samples, except for the Pt/TiO_2_ + Y system with different Y contents of 33, 50, and 67 wt%. These mixed samples were denoted as Pt/oxide + zeolite or oxide. To simulate the catalyst throughout its operational lifespan in heavy-duty vehicles powered by diesel or hydrogen fuel, accelerated aging treatment under hydrothermal conditions of 550–650 °C for 50–100 h should be conducted. In this study, an aging treatment under 10% H_2_O and 10% O_2_ at 650 °C for 50 h was performed, and the resulting catalysts were labeled with “-Aged”.

### Catalyst characterizations

X-ray diffraction (XRD) measurement was performed on a PANalytical Empyrean diffractometer using a Cu Kα radiation source (λ = 0.15406 nm). The measurement covered the 5^o^ to 80^o^ range with a scan mode of 6 ^o^/min and a scan step of 0.067^o^.

N_2_ physisorption was used to determine the surface area, pore volume, and pore size distribution, which was performed on a Quantachrome Autosorb-iQ instrument at liquid nitrogen temperature (77 K). Prior to measurement, all samples were degassed at 300 °C for 2 h under vacuum. The N_2_ adsorption-desorption isotherm was measured with 40 adsorption and 40 desorption points for Y and Pt/TiO_2_ + Y samples, and with 20 adsorption and 20 desorption points for Pt/TiO_2_ using the pressure intervals of 0 < P/P_0_ < 1. The surface area was calculated using the Brunauer–Emmett–Teller (BET) method based on the adsorption points in the relative pressure range between 0.05 and 0.3. The Horvath-Kawazoe (HK) method and non-local density functional theory (DFT) method were used to determine the pore volume and pore size distribution.

Transmission electron microscopy (TEM) and energy dispersive X-ray spectroscopy (EDS) mapping images were collected on a field emission FEI Tecnai F-30 with HAADF/ADF/BF STEM and EDS detectors operated at 200 kV.

The CO chemisorption measurement was performed on a Quantachrome Autosorb-iQ instrument. Before each measurement, the sample was first exposed to flowing He from room temperature to 150 °C at the ramp rate of 5 °C/min, and then held at 150 °C for 10 min. Next, the system was purged with 10% H_2_/Ar, and the temperature was ramped to 400 °C at the rate of 5 °C/min and kept for 30 min. It is important to note that a certain degree of Pt sintering might occur during this reduction treatment, potentially resulting in a lower-estimated Pt dispersion value. The system was then switched back to He, while maintaining the temperature at 400 °C for 30 min. The final step involved cooling the system down to 35 °C in He at the rate of 20 °C/min, holding at 35 °C for 30 min, and injecting multiple CO pulses (5% CO/He) using thermal conductivity detector (TCD) to monitor the gas phase CO.

The X-ray absorption near-edge structure (XANES) and extended X-ray absorption fine structure (EXAFS) of Pt L_3_-edge were measured at room temperature in fluorescent mode at beamline 7-BM QAS of the National Synchrotron Light Source II (NSLS-II), Brookhaven National Laboratory. Pt foil was measured during data collection for energy calibration and drift correction of the monochromator. Data analysis was conducted using Athena and Artemis from the Demeter software package. The processed EXAFS, χ(*k*), was weighted by *k*^2^ to amplify the high-*k* oscillations. For Fourier-transformed (FT) spectra, the *k* range between 3.0 and 12.0 Å was used, and the curve fitting was performed using the Artemis software.

X-ray photoelectron spectroscopy (XPS) was measured on a Thermo Scientific ESCALAB 250Xi photoelectron spectrometer using Al K-α (hν = 1486.68 eV) as the X-ray source in ultrahigh vacuum condition (10^−7 ^Pa). The binding energy (BE) of Pt 4*d* spectra was corrected using the C 1*s* signal at 284.6 eV as reference.

H_2_ temperature-programmed reduction (H_2_-TPR) was performed on the Quantachrome Autosorb-iQ instrument. Prior to testing, the samples were pretreated in a flow of 5% O_2_/He at 300 °C for 1 h. After cooling down to 40 ^o^C, a flow of 10% H_2_/Ar was used, and the temperature was raised linearly from 40 to 700 ^o^C at the ramp rate of 10 ^o^C/min. The H_2_ consumption was monitored on-line using TCD.

In situ DRIFTS experiments were performed on a Nicolet iS50 FTIR spectrometer equipped with a liquid nitrogen-cooled mercury-cadmium-telluride (MCT) detector and an in situ IR cell with ZnSe windows (DiffusIR, PIKE Technologies). Prior to measurements, all samples were pretreated in Ar flow at 300 ^o^C for 1 h. The background spectra at different temperatures (e.g., 25, 100, 125, 150, 175, 200, 225, 250, 275, and 300 ^o^C) were collected in Ar flow using 100 scans with a resolution of 4 cm^−1^. For in situ DRIFTS of CO adsorption at 25 ^o^C, 1% CO/Ar was introduced into the IR cell and kept for 30 min. Then, the samples were purged by Ar for 30 min to remove the weakly adsorbed CO, followed by spectra collection. For in situ DRIFTS of NO adsorption/desorption, the feed stream of 1000 ppm NO, 10% O_2_, and 5% H_2_O (when used) in Ar was introduced into the cell with a flow rate of 50 mL/min and kept for 60 min to achieve the saturated NO adsorption at 100 ^o^C. The NO flow was then discontinued while Ar (50 mL/min) was kept flowing for 30 min to remove the gaseous and weakly adsorbed NO. Afterwards, the desorption experiments were carried out in Ar flow with the temperature elevated from 100 to 300 °C with an interval of 25 ^o^C, and the spectra were collected under steady state accordingly. For in situ DRIFTS of H_2_O adsorption, a feed stream of 5% H_2_O in Ar was introduced into the cell at a flow rate of 50 mL/min and kept for 60 min, achieving saturated H_2_O adsorption at 120 ^o^C. Then, the sample was purged with Ar for 30 min at 120 ^o^C to remove the weakly adsorbed H_2_O, and a background spectrum was collected. The sample was finally treated in Ar flow at 500 °C for 2 h and cooled down to 120 ^o^C, followed by the spectrum collection.

NO temperature-programmed desorption (NO-TPD) and H_2_O temperature-programmed desorption (H_2_O-TPD) were conducted on a continuous flow fixed-bed system. A quartz tubular microreactor with an internal diameter of 4.0 mm was used, and a Hidden Analytical mass spectrometer (MS) was employed as detector. Typically, a feed stream of 1000 ppm NO, 10% O_2_, and 5% H_2_O (when used) in Ar was introduced into the reactor at a flow rate of 40 mL/min and kept for 60 min, achieving saturated NO adsorption at 50 ^o^C. Afterwards, the sample was purged with Ar (40 mL/min) for 120 min at 50 ^o^C to remove the weakly adsorbed molecules. The temperature was then elevated linearly from 50 to 600 ^o^C at a ramp rate of 10 ^o^C/min. For H_2_O-TPD, a feed stream of 5% H_2_O in Ar was introduced into the reactor at a flow rate of 40 mL/min and kept for 60 min, achieving saturated H_2_O adsorption at 50 ^o^C. The sample was then purged with Ar (40 mL/min) for 120 min at 50 ^o^C to remove the weakly adsorbed H_2_O. Subsequently, the temperature was elevated linearly from 50 to 600 ^o^C at a ramp rate of 10 ^o^C/min. The NO or H_2_O desorption was monitored on-line using *m*/*z* of 30 or 18, respectively.

### Catalytic performance evaluation

The catalytic activity evaluation for the H_2_-SCR of NO_*x*_ over all catalysts was conducted using a continuous flow fixed-bed quartz tubular microreactor with an internal diameter of 4.0 mm. In each test, the catalyst or physical mixture containing 26 mg of Pt/oxide catalyst (40–60 mesh) was diluted with 0.25 g of inert SiC (40-60 mesh) to minimize the effect of hot spots. The reaction atmosphere comprised of 500 ppm NO, 1% H_2_, 10% O_2_, 5% CO_2_ (when used) and 5% H_2_O (when used), using Ar as balance. The total flow rate was controlled at 200 mL/min, resulting in a weight hourly space velocity (WHSV) of 461,540 mL·g_Pt/oxide_^–1^·h^–1^. During the steady-state testing, the catalyst was held at each temperature for a duration of 30 min. Reactants and products were analyzed online by a MultiGas 2030 CEM-Cert FTIR spectrometer. The reactant conversion was defined as (c_inlet_ – c_outlet_)/c_inlet_ × 100%, where c_inlet_ and c_outlet_ were the inlet and outlet NO_*x*_ concentration in the feed stream, respectively. The N_2_ selectivity was defined as ([NO]_inlet_ + [NO_2_]_inlet_ – [NO]_outlet_ – [NO_2_]_outlet_ – 2 × [N_2_O]_outlet_)/([NO]_inlet_ + [NO_2_]_inlet_ – [NO]_outlet_ – [NO_2_]_outlet_) × 100%. Under the H_2_-SCR testing conditions with 1% H_2_ and 10% O_2_, NO could be either selectively reduced by H_2_ to form N_2_/N_2_O or oxidized by O_2_ to form NO_2_. The NO selective conversion attributed to the NO reduction by H_2_ (NO + H_2_) under the H_2_-SCR condition was defined as ([NO]_inlet_ + [NO_2_]_inlet_ – [NO]_outlet_ – [NO_2_]_outlet_)/([NO]_inlet_ – [NO]_outlet_) × 100%, and the NO conversion attributed to the NO oxidation by O_2_ (NO + O_2_) under the H_2_-SCR condition was defined as ([NO_2_]_outlet_ – [NO_2_]_inlet_)/([NO]_inlet_ – [NO]_outlet_) × 100%. To avoid the significant heat or mass transfer limitation, the kinetics study was performed at 100 ^o^C under the WHSV of 2,400,000 mL·g_Pt/TiO2_^–1^·h^–1^ to determine the NO, H_2_, and O_2_ reaction orders on Pt/TiO_2_ and Pt/TiO_2_ + Y catalysts. The catalytic performance evaluations for separate NO oxidation, H_2_-SCR in the presence of NO_2_, H_2_-SCR in the presence of NH_3_, separate H_2_ oxidation, as well as the H_2_-SCR reaction on the catalysts with pre-dehydration and pre-adsorption of H_2_O, were also conducted. The detailed information can be found in Supplementary Text 1.

### DFT calculations

Periodic non-spin-polarized DFT calculations were performed using the Vienna Ab-initio Simulation Package (VASP) and the Perdew-Burke-Ernzerhof functional within generalized gradient approximation (GGA). The valence electrons were described by projector augmented wave pseudopotentials with an energy cutoff of 400 eV for all the calculations. The Methfessel-Paxton smearing scheme was used with a width of 0.15 eV and the precision was set to “accurate”. The convergence criteria for energies and forces in structure optimizations were set as 10^−5 ^eV and 0.02 eV Å^−1^, respectively. The van der Waals (vdW) interactions were included via using Grimme’s DFT-D3 method. The Brillouin zone for periodic slab calculations was sampled on Γ-centered Monkhorst-Pack type 2 × 3 × 1 k-point grid. Transition states of surface reactions were searched by the nudged elastic band (NEB) together with the dimer method. Further vibrational analysis was adopted to confirm the transition states. Only one imaginary frequency mode along the reaction trajectory represented the true saddle point.

The reaction energy (Δ*E*) of each elementary step was computed by the difference between the DFT energy of the final state (*E*_FS_) and that of the corresponding initial state (*E*_IS_), with Δ*E* = *E*_FS_ – *E*_IS_. Similarly, the activation energy was calculated using the equation, *E*_a_ = *E*_TS_ – *E*_IS_, where *E*_TS_ was the DFT energy of corresponding transition state (TS). H binding energy (*E*_*b*_(H)) was computed by the equation, *E*_*b*_(H) = *E*_H/support_ – *E*_support_ – 0.5*E*_H2_, where *E*_H/support_, *E*_support_ and *E*_H2_ were the DFT energies of support with the *H adsorbate, the support, and gas phase H_2_, respectively. Gibbs free energy of each species was calculated by

1 $$G=E+{E}_{{{{\rm{ZPE}}}}}+{C}_{{{{\rm{p}}}}}T-{TS}$$

in which *G* was the Gibbs free energy, and *E*, *E*_ZPE_, *C*_p_ and *S* were the DFT energy, zero point energy, heat capacity and entropy of each gas-phase species or surface intermediates, respectively. The *E*_ZPE_, *C*_p_, and *S* were calculated within the harmonic approximation. The Atomic Simulation Environment (ASE) package was employed to calculate the Gibbs free energy of gas and adsorbed species at certain temperatures and pressures.

The Gibbs free formation energies of adsorbates on corresponding surface were calculated via the following equation:

2 $${G}_{{{{\rm{f}}}}}({{{{\rm{N}}}}}_{x}{{{{\rm{O}}}}}_{y}{{{{\rm{H}}}}}_{z}/{{{\rm{surface}}}})=\, G({{{{\rm{N}}}}}_{x}{{{{\rm{O}}}}}_{y}{{{{\rm{H}}}}}_{z}/{{{\rm{surface}}}})-G({{{\rm{surface}}}})-{xG}({{{\rm{NO}}}})\\ -(y-x)\times G({{{{\rm{H}}}}}_{2}{{{\rm{O}}}})-(z/2-y+x)\times G({{{{\rm{H}}}}}_{2})$$

in which *G*(N_*x*_O_*y*_H_*z*_/surface), *G*(surface), *G*(NO), *G*(H_2_O), and *G*(H_2_) were the Gibbs free energies of the surface with adsorbates, the clean surface, and gas phase NO, H_2_O, and H_2_ under relevant temperatures and pressures, respectively. The partial pressures of gas phase NO, H_2_, and H_2_O were set as 50, 1000, and 5000 Pa, which were within the range of experimental operation conditions.

### Reporting summary

Further information on research design is available in the Nature Portfolio Reporting Summary linked to this article.

## Supplementary information

Supplementary Information

Peer Review File

Reporting Summary

## Source data

Source Data

## Data Availability

Source data are provided with this paper.
