# Supplementary material for: Zeolite-promoted platinum catalyst for efficient reduction of nitrogen oxides with hydrogen
Source: Nat Commun. 2024 Sep 12;15:7988. doi: 10.1038/s41467-024-52382-7 (PMC11405393; doi:10.1038/s41467-024-52382-7)
Supplement: Supplementary file 1 — Supplementary Information [file 41467_2024_52382_MOESM1_ESM.docx]

**Supplementary Information**

**Zeolite-Promoted Platinum Catalyst for Efficient Reduction of Nitrogen Oxides with Hydrogen**

Shaohua Xie^1,2,†^, Liping Liu^3,†^, Yuejin Li^4^, Kailong Ye^1,2^, Daekun Kim^2^, Xing Zhang^2^, Hongliang Xin^3,*^, Lu Ma^5^, Steven N. Ehrlich^5^, Fudong Liu^1,2,*^

^1^ Department of Chemical and Environmental Engineering, Bourns College of Engineering, Center for Environmental Research and Technology (CE-CERT), Materials Science and Engineering (MSE) Program, University of California, Riverside, California 92521, United States

^2^ Department of Civil, Environmental, and Construction Engineering, Catalysis Cluster for Renewable Energy and Chemical Transformations (REACT), NanoScience Technology Center (NSTC), University of Central Florida, Orlando, Florida 32816, United States

^3^ Department of Chemical Engineering, Virginia Polytechnic Institute and State University, Blacksburg, Virginia 24061, United States

^4^ BASF Environmental Catalyst and Metal Solutions, Iselin, New Jersey 08830, United States

^5^ National Synchrotron Light Source II (NSLS-II), Brookhaven National Laboratory, Upton, New York 11973, United States

^†^ These authors contributed equally.

* Corresponding authors. Emails: [hxin@vt.edu](mailto:hxin@vt.edu) (H. X.); [fudong.liu@ucr.edu](mailto:fudong.liu@ucr.edu), [lfd1982@gmail.com](mailto:lfd1982@gmail.com) (F. L.)

**Supplementary Text 1** **|** **Catalytic performance evaluations for separate NO oxidation, H_2_-SCR in the presence of NO_2_, H_2_-SCR in the presence of NH_3_, separate H_2_ oxidation, and H_2_-SCR reaction on catalysts with pre-dehydration and pre-adsorption of H_2_O.**

**Separate NO oxidation**: The catalytic activity evaluation for separate NO oxidation was conducted using a continuous flow fixed-bed quartz tubular microreactor with an internal diameter of 4.0 mm. In each test, 30 mg of Pt/TiO_2_ or Y, or a mixture containing 30 mg of Pt/TiO_2_ and 30 mg of Y (40-60 mesh) was diluted with 0.25 g of inert SiC (40-60 mesh) to minimize the effect of hot spots. The reaction atmosphere comprised of 500 ppm NO, and 10% O_2_, using Ar as balance. The total ﬂow rate was controlled at 200 mL/min, resulting in a weight hourly space velocity (WHSV) of 400,000 mL·g_Pt/TiO2_^–1^·h^–1^. During the steady-state testing, the reaction was held at each temperature for a duration of 30 min. Reactants and products were analyzed online by a MultiGas 2030 CEM-Cert FTIR spectrometer. The reactant conversion was deﬁned as ([NO]_inlet_ – [NO]_outlet_)/[NO]_inlet_ × 100%, where [NO]_inlet_ and [NO]_outlet_ were the inlet and outlet NO concentrations in the reaction stream, respectively.

**H_2_-SCR in the presence of NO_2_**: Using the same evaluation system as for separate NO oxidation, H_2_-SCR performance under the testing conditions with the presence of NO_2_ in the reaction flow was determined. In each test, 26 mg of Pt/TiO_2_ or Y, or a mixture containing 26 mg of Pt/TiO_2_ and 26 mg of Y (40-60 mesh) was diluted with 0.25 g of inert SiC (40-60 mesh) to minimize the effect of hot spots. The reaction atmosphere comprised of 250 ppm NO, 250 ppm NO_2_, 1% H_2_, 10% O_2_, 5% CO_2_ and 5% H_2_O, using Ar as balance. The total ﬂow rate was controlled at 200 mL/min, resulting in a WHSV of 461,540 mL·g_Pt/TiO2_^–1^·h^–1^. During the steady-state testing, the reaction was held at each temperature for a duration of 30 min. Reactants and products were analyzed online by a MultiGas 2030 CEM-Cert FTIR spectrometer. The reactant conversion was deﬁned as (*c*_inlet_ – *c*_outlet_)/*c*_inlet_ × 100%, where *c*_inlet_ and *c*_outlet_ were the inlet and outlet NO*_x_* concentration in the reaction stream, respectively.

**H_2_-SCR in the presence of NH_3_**: The impact of NH_3_ on the H_2_-SCR activity was assessed through transient testing at 100 °C and subsequently at 200 °C using the same system as mentioned above. In the experiment, 10 mg of Pt/TiO_2_ or a mixture containing 10 mg of Pt/TiO_2_ and 10 mg of Y (40-60 mesh) was utilized after dilution with 0.25 g of inert SiC (40-60 mesh). The reaction atmosphere consisted of 500 ppm NO, 1% H_2_, 500 ppm NH_3_ (if used), 10% O_2_, and 5% H_2_O, using Ar as balance. The total ﬂow rate was controlled at 200 mL/min, resulting in a WHSV of 1,200,000 mL·g_Pt/TiO2_^–1^·h^–1^. In the H_2_-SCR flow, the catalyst was initially heated to 100 °C and kept at this temperature for 120 min. Subsequently, 500 ppm NH_3_ was introduced into the flow and maintained for 60 min before being discontinued. After 120 min, the catalyst was further heated to 200 °C, and the aforementioned procedures conducted at 100 °C were repeated. The concentrations of NO, NO_2_, and NH_3_ were analyzed online by a MultiGas 2030 CEM-Cert FTIR spectrometer.

**Separate H_2_ oxidation**: The catalytic activity evaluation for separate H_2_ oxidation was performed in a continuous flow fixed-bed quartz tube microreactor. To minimize the effect of hot spots, 30 mg of Pt/TiO_2_, or a mixture containing 30 mg of Pt/TiO_2_ and 30 mg of Y (40-60 mesh) was diluted with 0.25 g of inert SiC (40-60 mesh). The reaction atmosphere consisted of 1 % H_2_ and 10 % O_2_, using Ar as balance. The total ﬂow rate was controlled at 200 mL/min, resulting in a WHSV of 400,000 mL·g_Pt/TiO2_^–1^·h^–1^. Reactants and products were analyzed online using a mass spectrometer (Hiden Analytical, HPR20 R&D). The *m*/*z* ratios used for H_2_, O_2_, and H_2_O detection were 2, 32, and 18, respectively. The H_2_ conversion was calculated as ([H_2_]_inlet_ – [H_2_]_outlet_)/[H_2_]_inlet_ × 100%, where [H_2_]_inlet_ and [H_2_]_outlet_ were the inlet and outlet H_2_ concentrations in the reaction stream, respectively.

**H_2_-SCR reaction on catalysts with pre-dehydration and pre-adsorption of H_2_O**: The catalytic activity evaluation for the H_2_-SCR reaction over Pt/TiO_2_ and Pt/TiO_2_ + Y catalysts was conducted using the same system as for separate NO oxidation testing and H_2_-SCR testing in the presence of NO_2_ or NH_3_, as mentioned above. In each test, 26 mg of Pt/TiO_2_, or a mixture containing 26 mg of Pt/TiO_2_ and 26 mg of Y (40-60 mesh) was diluted with 0.25 g of inert SiC (40-60 mesh) to minimize the effect of hot spots. The reaction atmosphere comprised of 500 ppm NO, 1% H_2_, and 10% O_2_, using Ar as balance. The total ﬂow rate was controlled at 200 mL/min, resulting in a weight hourly space velocity (WHSV) of 461,540 mL·g_Pt/TiO2_^–1^·h^–1^. During the transient-state light-off testing, the catalyst was heated linearly from 30 to 300 ^o^C at a ramp rate of 2 ^o^C/min. For the testing on catalyst after pre-dehydration, the catalyst was linearly heated from room temperature to 300 °C at a rate of 10 °C/min in an Ar flow (200 mL/min) and held at 300 °C for 60 min. Subsequently, as the catalyst naturally cooled down to 30 °C in the Ar flow, the transient-state light-off testing was performed. As for the testing on catalyst after pre-adsorption of H_2_O, following the dehydration treatment at 300 ^o^C, the catalyst was exposed to a flow containing 5% H_2_O in Ar at 30 ^o^C for 60 min to achieve saturated H_2_O adsorption. Afterwards, the H_2_O flow was disconnected, and the catalyst was purged with Ar for 60 min before the transient-state light-off testing. Reactants and products were analyzed online by a MultiGas 2030 CEM-Cert FTIR spectrometer. The reactant conversion was deﬁned as (*c*_inlet_ - *c*_outlet_)/*c*_inlet_ × 100%, where *c*_inlet_ and *c*_outlet_ were the inlet and outlet NO*_x_* concentration in the reaction stream, respectively. The N_2_ selectivity was defined as ([NO]_inlet_ + [NO_2_]_inlet_ – [NO]_outlet_ – [NO_2_]_outlet_ – 2 × [N_2_O]_outlet_)/([NO]_inlet_ + [NO_2_]_inlet_ – [NO]_outlet_ – [NO_2_]_outlet_) × 100%.

**Supplementary Table 1 |** Comparison of the H_2_-SCR activity on Pt and Pd based catalysts reported in literature and Pt/TiO_2_ +Y catalyst in this work.

| Sample  (Composition) | Reaction Conditions | T50 (^o^C)^a^ | T90 (^o^C)^a^ | H_2_-SCR at 100 ^o^C | | H_2_-SCR at 200 ^o^C | | Ref. |
| --- | --- | --- | --- | --- | --- | --- | --- | --- |
|  |  |  |  | Reaction Rate  (mmol/(g_metal_ s)) | N_2_ selectivity (%) | Reaction Rate  (mmol/(g_metal_ s)) | N_2_ selectivity (%) |  |
| Pt/TiO_2_ +Y  (1 wt.%Pt/TiO_2_ + Y-50 wt.%) | 0.05% NO, 1% H_2_, 10% O_2_, 5% CO_2_, and 5% H_2_O; WHSV = 461,540 mL·g_Pt/TiO2_^–1^·h^–1^ | <100 | − | 0.19 | 72 | 0.20 | 62 | This work |
| Pt/Al-M-10i  (0.92 wt.%Pt/Al-MCM-41) | 0.1% NO, 0.5% H_2_, 6.7% O_2_; WHSV = 120,000 mL·g^–1^·h^–1^ | 90 | − | 0.095 | 46 | 0.086 | 93 | ^1^ |
| 0.5%Pt/HY-R  (0.5 wt.%Pt/HY-R) | 0.1% NO, 0.5% H_2_, 10% O_2_; WHSV = 60,000 mL·g^–1^·h^–1^ | 80 | − | 0.12 | 72 | 0.016 | − | ^2^ |
| Pt/ZSM-5-850C  (1.5 wt.%Pt/ZSM-5) | 0.05% NO, 2% H_2_, 5% O_2_, and 5% H_2_O; WHSV = 120,000 mL·g^–1^·h^–1^ | <75 | 150 | 0.048 | 81 | 0.042 | 76 | ^3^ |
| 0.1%Pt/Ce_0.5_Zr_0.5_O_2-δ_  (0.1 wt.%Pt/Ce_0.5_Zr_0.5_O_2-δ_) | 0.015% NO, 0.8% H_2_, 2.5% O_2_, 10% CO_2_, and 15% H_2_O; WHSV = 40,000 mL·g^–1^·h^–1^ | 165 | 220 | − | − | 0.031 | 80 | ^4^ |
| Pt0.5W2  (0.5 wt.%Pt-2 wt.%WO_3_/TiO_2_) | 0.25% NO, 1% H_2_, 5% O_2_; WHSV = 60,000 mL·g^–1^·h^–1^ | <100 | − | 0.041 | − | 0.19 | − | ^5^ |
| 1%Pd/20%TiO_2_/HY  (1 wt.%Pd/20 wt.%TiO_2_/HY) | 0.1% NO, 0.5% H_2_, 10% O_2_, and 10% H_2_O; WHSV = 200,000 mL·g^–1^·h^–1^ | 187 | − | 0.011 | − | 0.18 | 83 | ^6^ |
| Pd/ZrO_2_-CeO_2_  (0.5 wt.%Pd/40ZrO_2_-CeO_2_) | 0.1% NO, 0.4% H_2_, 6% O_2_; WHSV = 30,000 mL·g^–1^·h^–1^ | 114 | − | 0.027 | 83 | 0.066 | 92 | ^7^ |
| Pd/TiO_2_(PR)  (0.5 wt.%Pd/TiO_2_) | 0.2% NO, 0.8% H_2_, 5% O_2_; WHSV = 60,000 mL·g^–1^·h^–1^ | <100 | − | 0.18 | 35 | 0.23 | 32 | ^8^ |
| 1Pd-5Ni/TiO_2_  (1 wt.%Pd-5 wt.%NiO/TiO_2_) | 0.02% NO, 0.2% H_2_, 1.5% O_2_; WHSV = 60,000 mL·g^–1^·h^–1^ | 120 | 175 | 0.005 | 69 | 0.015 | 85 | ^9^ |
| Pd-SiO_2_/TiO_2_  (1 wt.%Pd-8 wt.%SiO_2_/TiO_2_) | 0.05% NO, 1% H_2_, 10% O_2_, 5% CO_2_, and 5% H_2_O; WHSV = 461,540 mL·g^–1^·h^–1^ | 140 | − | 0.020 | 54 | 0.24 | 69 | ^10^ |

^a^ T50 and T90 are the reaction temperatures corresponding to 50% and 90% of NO*_x_* conversion.

**Supplementary Table 2 |** TiO_2_ grain size, BET surface area, pore volume, and average pore diameter for Pt/TiO_2_, Pt/TiO_2_-*p*, Pt/TiO_2_ + Y, (Pt/TiO_2_ + Y)-*p*, and Y samples.

| **Sample** | **TiO_2_ grain size^a^ (nm)** | **BET surface area^b^ (m^2^/g)** | **Pore volume (cm^3^/g)** | | **Average micropore/ mesopore diameter (nm)** |
| --- | --- | --- | --- | --- | --- |
|  |  |  | **Micropore^c^** | **Total^d^** |  |
| Pt/TiO_2_ | 20.0 | 81 | 0.032 | 0.178 | -/7.1^d^ |
| Pt/TiO_2_-*p*^e^ | 19.9 | 74 | 0.026 | 0.233 | -/6.8^d^ |
| Pt/TiO_2_ + Y | 20.6 | 349 | 0.164 | 0.318 | 0.6^c^/3.8^d^ |
| (Pt/TiO_2_ + Y)-*p*^e^ | 20.3 | 325 | 0.150 | 0.350 | 0.6^c^/3.8^d^ |
| Y | - | 709 | 0.329 | 0.513 | 0.6^c^/3.8^d^ |

^a^ Determined according to the Scherrer equation using the full width at half maximum (FWHM) of the (101) peak of TiO_2_.

^b^ Calculated using the Brunauer-Emmett-Teller (BET) method.

^c^ Determined using the Horvath-Kawazoe (HK) method.

^d^ Calculated using the non-local density functional theory (DFT) method.

^e^ The samples suffixed with “-*p*” represent the sample after reaction at 300 °C under testing conditions with H_2_O.

**Supplementary Table 3 |** EXAFS fitting results for Pt/TiO_2_ and Pt/TiO_2_ + Y catalysts using Pt foil, PtO_2_ as references (*k* range = 3.0-12.0 Å^-1^ for Fourier transform).

| **Sample** | **Shell** | **CN^a^** | **R (Å)^b^** | **ΔE_0_ (eV)^c^** | **σ^2^ (10^-3^ Å^2^)^d^** | **R-factor^e^** |
| --- | --- | --- | --- | --- | --- | --- |
| Pt | Pt-Pt | 12.0 ± 0.6 | 2.76 ± 0.01 | 8.0 ± 0.5 | 4.35 ± 0.34 | 0.004 |
| Pt/TiO_2_ | Pt-Pt | 11.4 ± 1.5 | 2.76 ± 0.01 | 7.7 ± 1.1 | 4.05 ± 0.81 | 0.025 |
| Pt/TiO_2_ + Y | Pt-Pt | 10.7 ± 2.0 | 2.76 ± 0.01 | 6.7 ± 1.5 | 5.38 ± 1.24 | 0.054 |
| PtO_2_ | Pt-O  Pt-O-Pt  Pt-O | 6.0 ± 0.5  6.0 ± 0.5  12.0 ± 0.9 | 2.01 ± 0.01  3.08 ± 0.01  3.73 ± 0.03 | 8.7 ± 1.0  5.0 ± 3.0  14.7± 1.7 | 2.69 ± 0.94  3.13 ± 0.73  6.09 ± 3.14 | 0.007 |

^a^ Coordination number; ^b^ Bond length; ^c^ Energy shift; ^d^ Debye-Waller factor; ^e^ Goodness-of-fit index.

**Supplementary Table 4 |** Linear combination fitting results of Pt L_3_-edge XANES and Pt average oxidation states.

| **Samples** | **Ratio of PtO_2_** | **Ratio of Pt foil** | **Averaged valence state of Pt** |
| --- | --- | --- | --- |
| Pt/TiO_2_ | 0.949 ± 0.002 | 0.051 ± 0.002 | 0.21 ± 0.01 |
| Pt/TiO_2_ + Y | 0.879 ± 0.001 | 0.121 ± 0.001 | 0.48 ± 0.01 |

**Supplementary Table 5 |** DFT-calculated energy changes for NO reduction on the Pt (111) surface representing the Pt/TiO_2_ catalyst. All energies are relative to the structure i’ in **Supplementary Fig. 24**, gas phase H_2_ and NO.

| **Structures in reaction mechanism** | **Relative potential energy (eV)** |
| --- | --- |
| i’ | 0.00 |
| ii’ | 0.00 |
| iii’ | –0.54 |
| iv’ | –0.50 |
| v’ | –0.81 |
| vi’ | –1.01 |
| vii’ | –1.32 |
| viii’ | –0.93 |
| ix’ | –1.62 |
| x’ | –3.15 |

**Supplementary Table 6 |** DFT-calculated energy changes for NO reduction on the H_2_O/Pt (111) surface representing the Pt/TiO_2_ + Y catalyst. All energies are relative to the structure i in **Fig. 6** in the main manuscript, gas phase H_2_ and NO.

| **Structures in reaction mechanism** | **Relative potential energy (eV)** |
| --- | --- |
| i | 0.00 |
| ii | –0.96 |
| iii | –2.42 |
| iv | –2.27 |
| v | –2.49 |
| vi | –1.68 |
| vii | –2.70 |
| viii | –2.68 |
| ix | –2.91 |
| x | –5.22 |
| xi | –5.74 |
| xii | –5.42 |
| xiii | –6.50 |

**
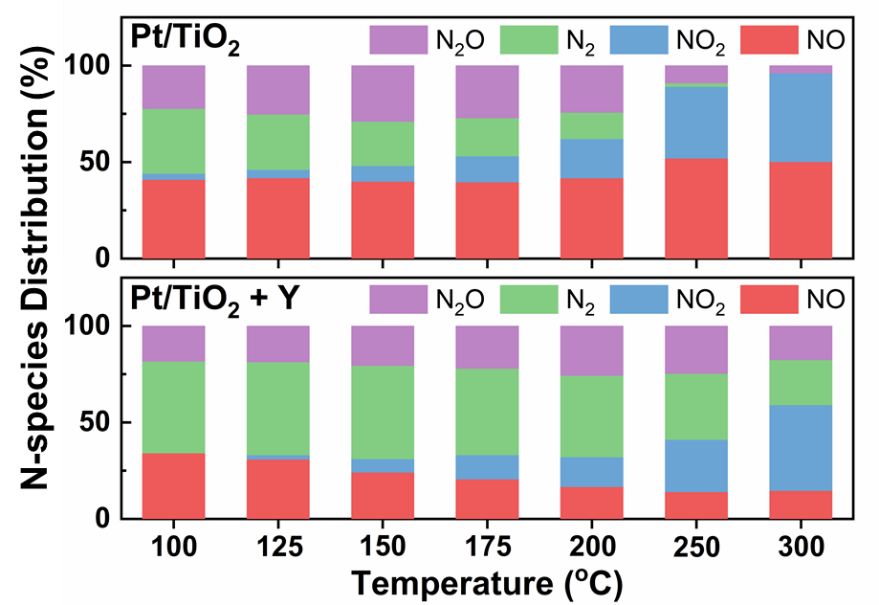
**

**Supplementary Fig. 1 | N-species distribution**. N-species distribution in N_2_O, N_2_, NO_2_, and NO during H_2_-SCR on Pt/TiO_2_ and Pt/TiO_2_ + Y catalysts. Reaction conditions: 26 mg of Pt/TiO_2_ catalyst or the mixture containing 26 mg of Pt/TiO_2_ catalyst and 26 mg of Y; 500 ppm NO, 1% H_2_, 10% O_2_, 5% CO_2_, and 5% H_2_O; WHSV = 461,540 mL·g_Pt/TiO2_^–1^·h^–1^.


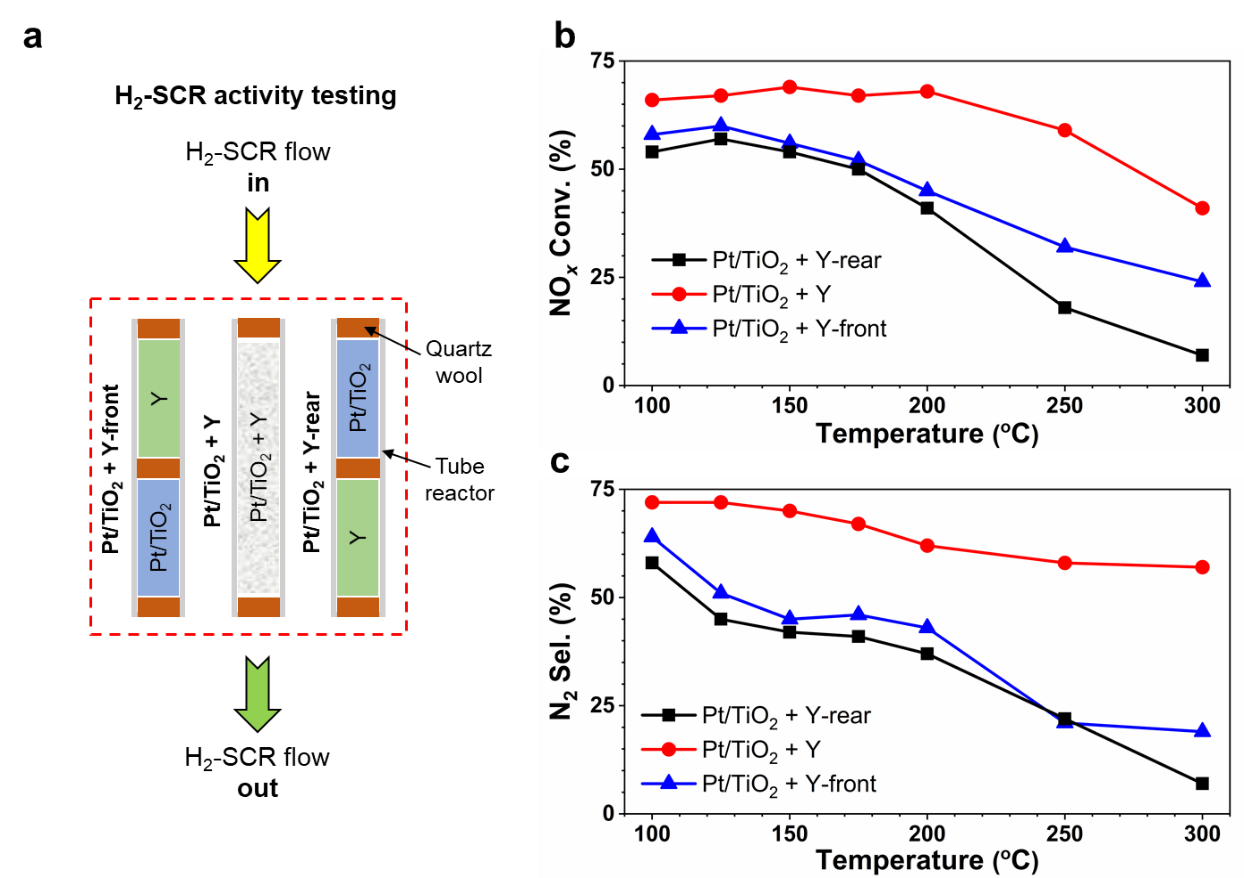


**Supplementary Fig. 2 | Effect of different physical mixing methods on H_2_-SCR performance**. (**a**) Scheme of H_2_-SCR activity testing on Pt/TiO_2_ + Y catalysts using different physical mixing methods. (**b**) NO*_x_* conversion and (**c**) N_2_ selectivity in H_2_-SCR reaction over Pt/TiO_2_ + Y catalysts using different mixing configurations. Reaction conditions: 26 mg of Pt/TiO_2_ catalyst and 26 mg of Y; 500 ppm NO, 1% H_2_, 10% O_2_, 5% CO_2_, and 5% H_2_O; WHSV = 461,540 mL·g_Pt/TiO2_^–1^·h^–1^.

**Notes:** In order to investigate the impact of different mixing methods for Pt/TiO_2_ and Y components, we conducted the H_2_-SCR activity testing on the mixtures with three different mixing configurations. **Supplementary Fig. 2a** illustrates the three mixing approaches in detail: (1) Y was packed in front of Pt/TiO_2_ and contacting the reaction flow first, earlier than Pt/TiO_2_ (Pt/TiO_2_ + Y-front); (2) Y was well mixed with Pt/TiO_2_ (Pt/TiO_2_ + Y) and contacting the reaction flow at the same time with Pt/TiO_2_; (3) Y was packed behind Pt/TiO_2_ and contacting the reaction flow in the rear, later than Pt/TiO_2_ (Pt/TiO_2_ + Y-rear). As depicted in **Supplementary Fig. 2b** and **Fig. 2c**, it was clearly observed that the H_2_-SCR activity and N_2_ selectivity decreased in the following order: Pt/TiO_2_ + Y >> Pt/TiO_2_ + Y-front > Pt/TiO_2_ + Y-rear. In clear contrast to the similar catalytic performance (*i.e.*, low NO*_x_* conversion and low N_2_ selectivity) on Pt/TiO_2_ + Y-front and Pt/TiO_2_ + Y-rear systems, much more excellent H_2_-SCR performance was observed on Pt/TiO_2_ + Y system prepared by the physical mixing of Pt/TiO_2_ and Y powders. These results suggest the critical role of establishing the close contact between Pt/TiO_2_ and Y zeolite in enhancing the overall H_2_-SCR performance.


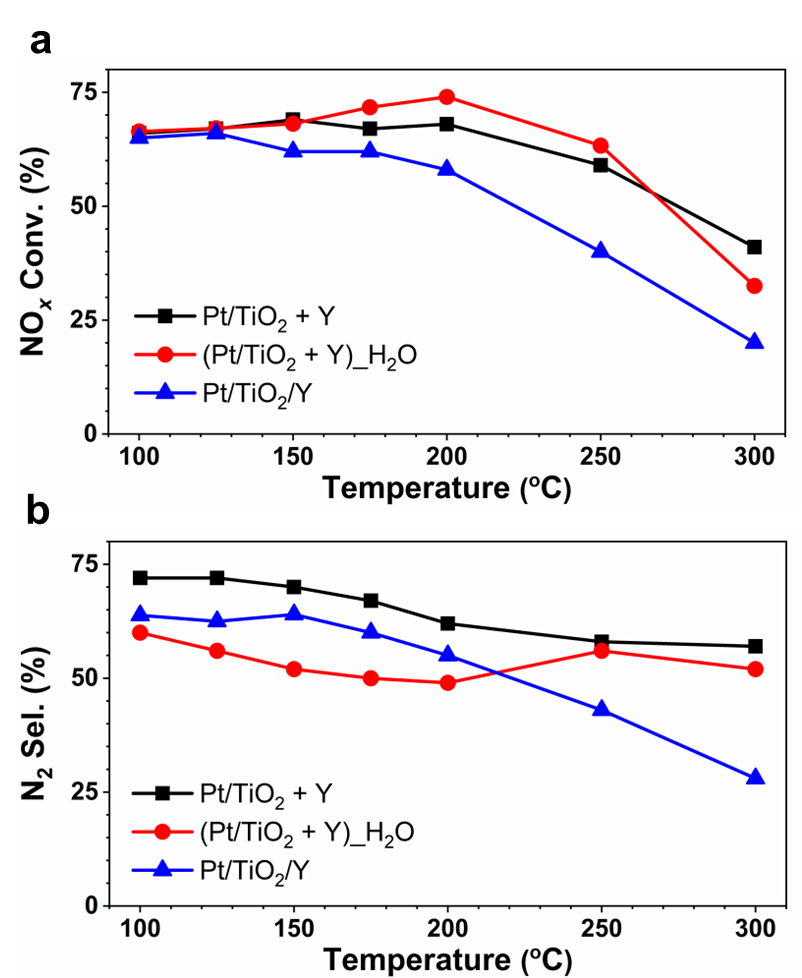


**Supplementary Fig. 3 | The effect of contact between Pt, TiO_2_, and Y in Pt-TiO_2_-Y system on the H_2_-SCR performance**. (**a**) NO*_x_* conversion and (**b**) N_2_ selectivity in H_2_-SCR reaction over Pt/TiO_2_ + Y, (Pt/TiO_2_ + Y)_H_2_O, and Pt/TiO_2_/Y catalysts. Reaction conditions: 52 mg of Pt/TiO_2_ + Y and (Pt/TiO_2_ + Y)_H_2_O, or 26 mg of Pt/TiO_2_/Y; 500 ppm NO, 1% H_2_, 10% O_2_, 5% CO_2_, and 5% H_2_O; WHSV = 461,540 mL·g_Pt/TiO2_^–1^·h^–1^.

**Notes**: To verify whether closer contact between Pt, TiO_2_, and Y could further improve H_2_-SCR performance, two additional Pt-TiO_2_-Y catalysts were prepared and tested for the H_2_-SCR reaction. The first catalyst, referred as (Pt/TiO_2_ + Y)_H_2_O, was fabricated by physically mixing Pt/TiO_2_ with Y in the presence of water, followed by drying at 300 °C for 2 h. The second catalyst, denoted as Pt/TiO_2_/Y, was obtained by impregnating colloidal Pt (2-6 nm) with 1 wt.% Pt onto a TiO_2_/Y support, followed by drying at 120 °C for 1 h and calcination in air at 550 °C for 2 h. The TiO_2_/Y support was prepared by precipitating 50 wt.% TiO_2_ from a Ti(OBu)_4_ solution in ethanol onto Y, followed by drying at 120 °C for 1 h and calcination in air at 550 °C for 2 h.


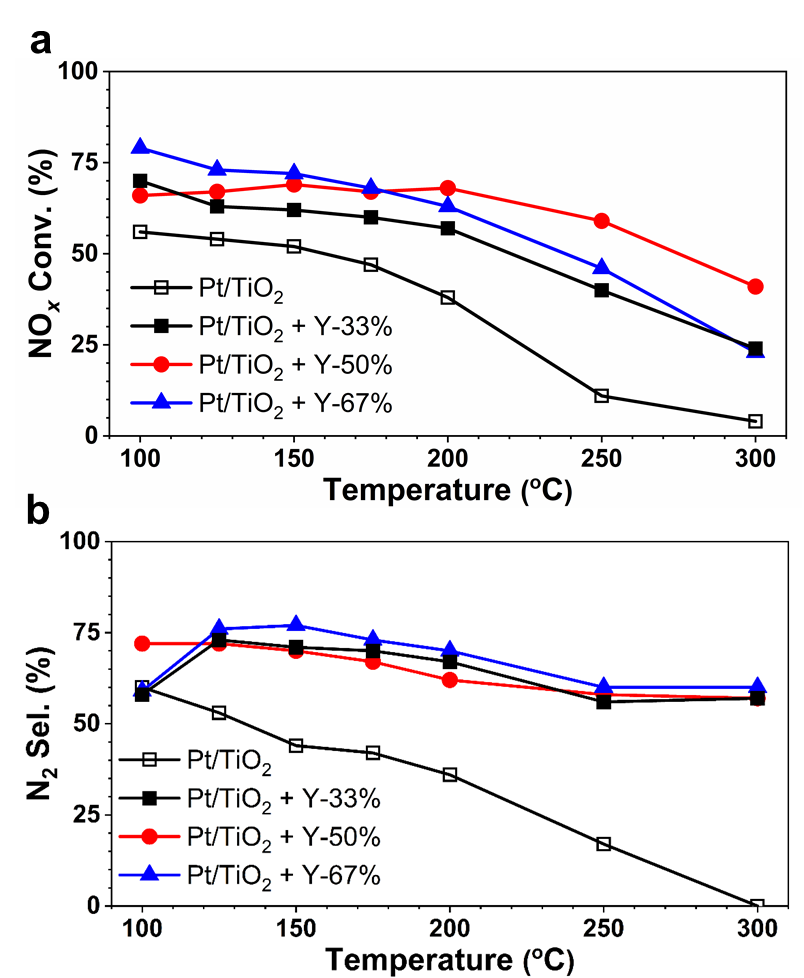


**Supplementary Fig. 4 | The effect of Y content in Pt/TiO_2_ + Y system on the H_2_-SCR performance**. (**a**) H_2_-SCR activity and (**b**) N_2_ selectivity on Pt/TiO_2_ + Y with different Y contents (0, 33, 50, 67 wt.%). Reaction conditions: 26 mg of Pt/TiO_2_ catalyst or the mixture containing 26 mg of Pt/TiO_2_ catalyst and 13 mg or 26 mg or 39 mg of Y; 500 ppm NO, 1% H_2_, 10% O_2_, 5% CO_2_, and 5% H_2_O; WHSV = 461,540 mL·g_Pt/TiO2_^–1^·h^–1^.

**Notes:** The effect of Y content in Pt/TiO_2_ + Y system on the H_2_-SCR performance was investigated in detail. As shown in **Supplementary Fig. 4a**, the addition of Y led to a significant increase in the NO*_x_* conversion on Pt/TiO_2_. The optimal NO*_x_* conversions across the entire temperature range were achieved when the Y content was set as 50 wt.%. Additionally, the inclusion of Y substantially enhanced the N_2_ selectivity on Pt/TiO_2_ (**Supplementary Fig. 4b**). For simplicity, the Pt/TiO_2_ + Y-50% mixture with Y content as 50 wt.% was denoted as Pt/TiO_2_ + Y throughout the manuscript.


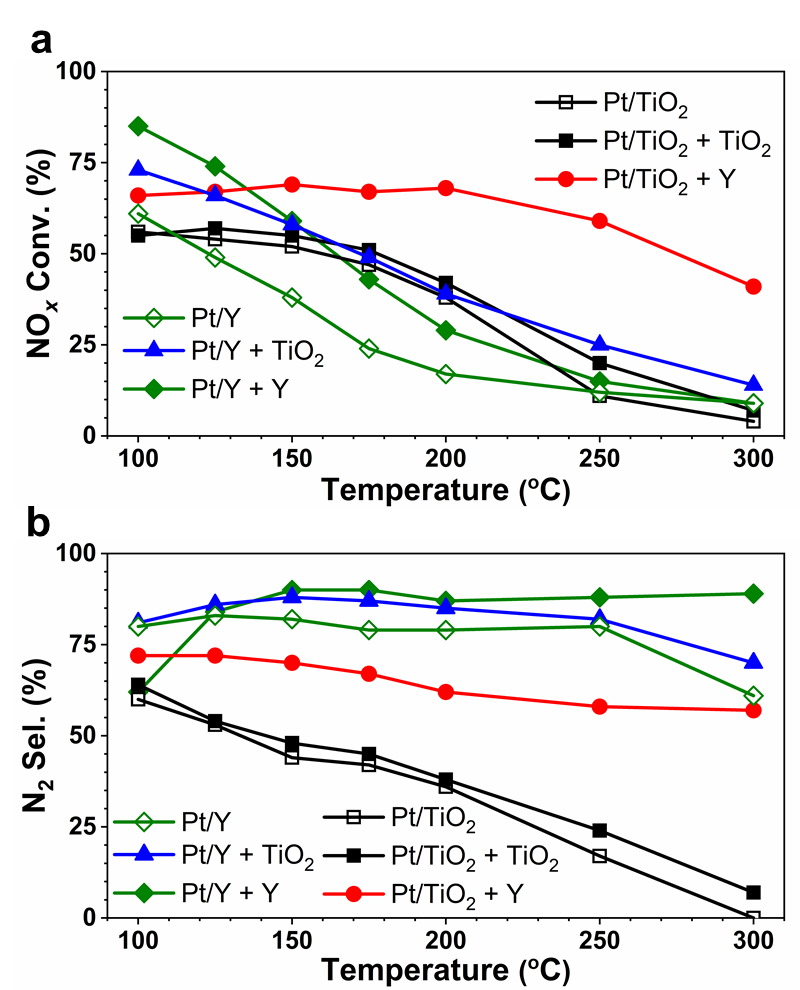


**Supplementary Fig. 5 | The impact of physical mixing of Pt/TiO_2_ with TiO_2_ or Y, and Pt/Y with TiO_2_ or Y on the H_2_-SCR performance**. (**a**) H_2_-SCR activity, (**b**) N_2_ selectivity on Pt/TiO_2_, Pt/TiO_2_ + TiO_2_, Pt/TiO_2_ + Y, Pt/Y, Pt/Y + TiO_2_, and Pt/Y + Y catalysts. Reaction conditions: 26 mg of Pt/TiO_2_ or Pt/Y catalyst, or the mixture composed of 26 mg of Pt-containing catalyst and 26 mg of TiO_2_ or Y; 500 ppm NO, 1% H_2_, 10% O_2_, 5% CO_2_ and 5% H_2_O; WHSV = 461,540 mL·g_Pt/oxide catalyst_^–1^·h^–1^.


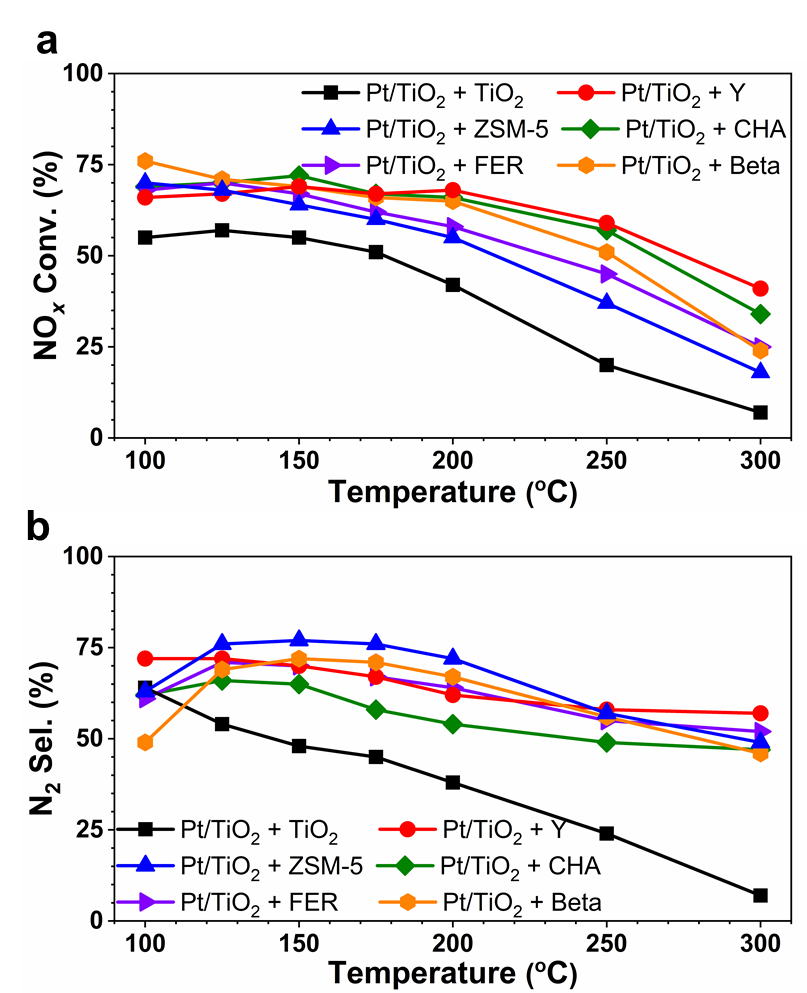


**Supplementary Fig. 6 | The effect of different types of zeolites in Pt/TiO_2_ + zeolite system on the H_2_-SCR performance**. (**a**) H_2_-SCR activity and (**b**) N_2_ selectivity on Pt/TiO_2_ + zeolite with different zeolite types including Y, ZSM-5, CHA, FER, and Beta. Reaction conditions: the mixture containing 26 mg of Pt/TiO_2_ catalyst and 26 mg of zeolite or TiO_2_; 500 ppm NO, 1% H_2_, 10% O_2_, 5% CO_2_, and 5% H_2_O; WHSV = 461,540 mL·g_Pt/TiO2_^–1^·h^–1^.

**Notes:** In addition to the Y zeolite, we further explored to see if the addition of other types of zeolite also showed benefits on improving the H_2_-SCR performance of Pt/TiO_2_ catalyst. As clearly depicted in **Supplementary Fig. 6a**, the addition of different zeolites all showed great benefits on improving the H_2_-SCR activity of Pt/TiO_2_ catalyst. The physical mixtures with different zeolites exhibited the following order of decreased H_2_-SCR activity: Pt/TiO_2_ + Y > Pt/TiO_2_ + CHA > Pt/TiO_2_ + Beta > Pt/TiO_2_ + FER > Pt/TiO_2_ + ZSM-5 >> Pt/TiO_2_. Meanwhile, the N_2_ selectivity decreased by the following order: Pt/TiO_2_ + ZSM-5 > Pt/TiO_2_ + Beta > Pt/TiO_2_ + Y > Pt/TiO_2_ + FER > Pt/TiO_2_ + CHA >> Pt/TiO_2_ (**Supplementary Fig. 6b**). Overall, considering both the H_2_-SCR activity and N_2_ selectivity, the addition of Y zeolite demonstrated the most significant promotional effect on the overall H_2_-SCR performance. Consequently, the Pt/TiO_2_ + Y formulation was chosen as the representative for the detailed characterizations and reaction mechanism study.


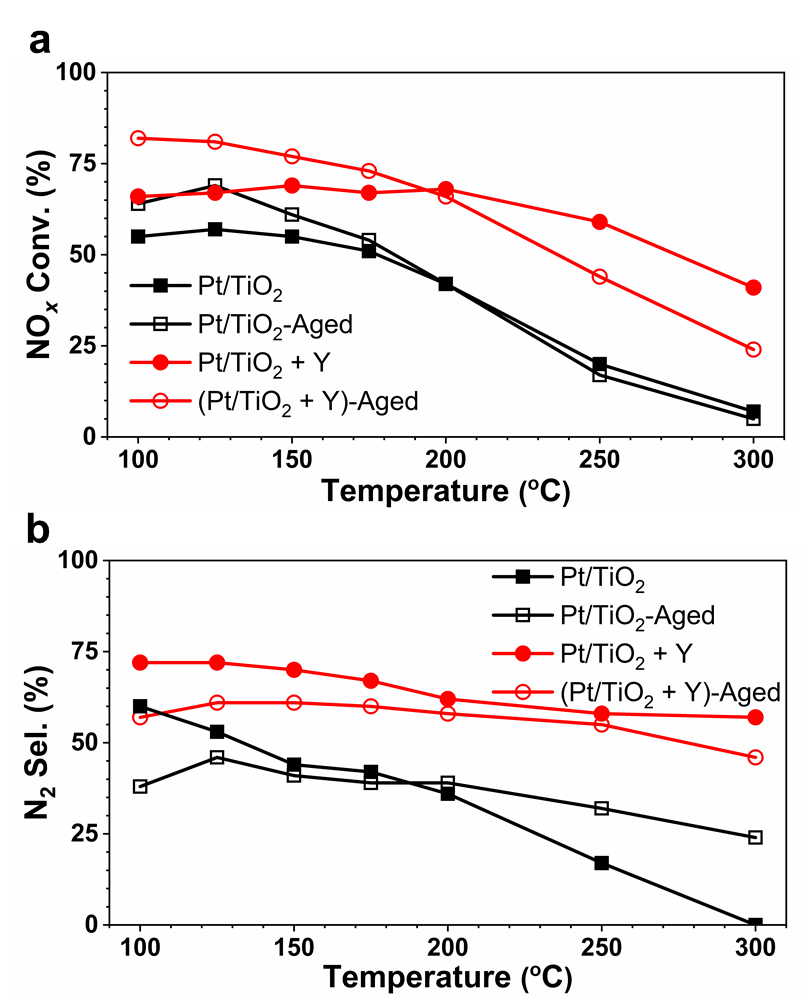


**Supplementary Fig. 7 | The effect of hydrothermal aging on the H_2_-SCR performance**. (**a**) H_2_-SCR activity and (**b**) N_2_ selectivity on Pt/TiO_2_ and Pt/TiO_2_ + Y catalysts before and after hydrothermal aging. Reaction conditions: 26 mg of Pt/TiO_2_ catalyst and 26 mg of Y; 500 ppm NO, 1% H_2_, 10% O_2_, 5% CO_2_, and 5% H_2_O; WHSV = 461,540 mL·g_Pt/TiO2_^–1^·h^–1^. Hydrothermal aging conditions: 10% H_2_O and 10% O_2_, at 650 ^o^C for 50 h.

**Notes:** In industrial applications, the catalytic performance of catalysts after long-term operation is of great importance. To simulate the state of catalysts used for H_2_-ICE exhaust purification after prolonged operation, we conducted hydrothermal aging on Pt/TiO_2_ and Pt/TiO_2_ + Y catalysts at 650 °C for 50 h in the flow containing 10% H_2_O and 10% O_2_. Interestingly, as presented in **Supplementary Fig. 7a**, in the low-temperature range (< 200 ^o^C), the H_2_-SCR activity of both Pt/TiO_2_ and Pt/TiO_2_ + Y catalysts significantly improved after hydrothermal aging. This improvement could be due to the enhanced H_2_ activation facilitated by the larger Pt particles generated from Pt sintering on TiO_2_ during aging. However, at relatively higher temperatures (> 200 ^o^C), hydrothermal aging had no significant effect on Pt/TiO_2_, while it led to the deactivation of Pt/TiO_2_ + Y catalyst to a certain extent. Notably, as shown in **Supplementary Fig. 7b**, the N_2_ selectivity on both catalysts decreased after hydrothermal aging, except for the case on Pt/TiO_2_ catalyst at high temperatures (> 200 ^o^C). Regardless of whether it was before or after hydrothermal aging, the inclusion of Y in Pt/TiO_2_ + Y system exhibited significant benefits in enhancing both the H_2_-SCR activity and N_2_ selectivity simultaneously.


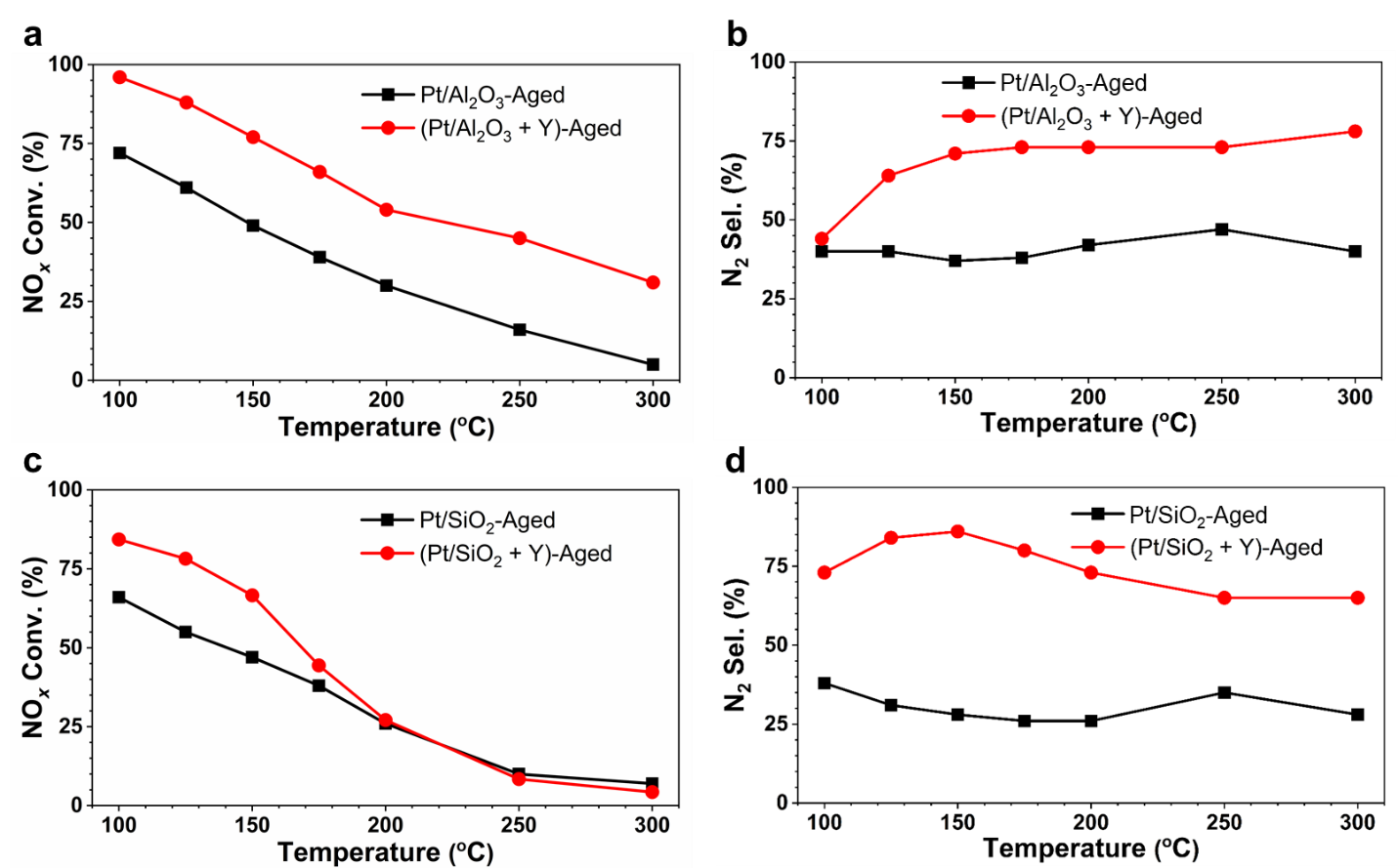


**Supplementary Fig. 8 | The concept generalization on Pt/Al_2_O_3_ and Pt/SiO_2_ catalysts**. (**a**, **c**) H_2_-SCR activity and (**b**, **d**) N_2_ selectivity on (**a**, **b**) aged Pt/Al_2_O_3_ and Pt/Al_2_O_3_ + Y catalysts, and (**c**, **d**) aged Pt/SiO_2_ and Pt/SiO_2_ + Y catalysts. Reaction conditions: 26 mg of Pt/oxide catalyst, or a mixture containing 26 mg of Pt/oxide and 26 mg of Y; 500 ppm NO, 1% H_2_, 10% O_2_, 5% CO_2_, and 5% H_2_O; WHSV = 461,540 mL·g_Pt/oxide_^–1^·h^–1^. Hydrothermal aging conditions: 10% H_2_O and 10% O_2_, at 650 ^o^C for 50 h.

**Notes:** To determine if the beneficial effect of adding Y zeolite could be extended to other Pt/oxide catalysts, beyond Pt/TiO_2_, we also conducted H_2_-SCR testing on aged Pt/Al_2_O_3_ and Pt/SiO_2_ catalysts with and without Y addition. **Supplementary Fig. 8a** and **b** illustrate the testing results on the aged Pt/Al_2_O_3_ and Pt/Al_2_O_3_ + Y catalysts, showing that the aged Pt/Al_2_O_3_ + Y catalyst exhibited significantly higher H_2_-SCR activity and N_2_ selectivity across the entire reaction temperature range comparing to the aged Pt/Al_2_O_3_ catalyst. Furthermore, **Supplementary Fig. 8c** and **d** demonstrate that the introduction of Y also showed a significant promotional effect on the aged Pt/SiO_2_ catalyst, especially for the low-temperature NO*_x_* conversion and the N_2_ selectivity within the whole temperature range. Clearly, the incorporation of Y zeolite with Pt/oxide catalysts is a universally effective strategy for improving the catalytic performance of Pt-based catalysts in the environmentally important H_2_-SCR reaction. Considering the successful enhancement of the overall H_2_-SCR performance on Pt/TiO_2_ catalyst through the addition of different zeolites as discussed earlier, it is believed that the simple physical mixing of Pt/oxide catalysts with zeolites represents a general and efficient approach to improve the H_2_-SCR performance.





**Supplementary Fig. 9 | Crystal structure.** XRD patterns of Pt/TiO_2_, Pt/TiO_2_-*p*, Pt/TiO_2_ + Y, (Pt/TiO_2_ + Y)-*p*, and Y samples. The samples suffixed with “-*p*” represent the sample after reaction at 300 °C under testing conditions with H_2_O.

**Notes:** As illustrated in **Supplementary Fig. 9**, Pt/TiO_2_ demonstrated a crystal structure of anatase TiO_2_ (JCPDS # 00-021-1272), while Y exhibited a standard FAU zeolite structure. The physical mixing of Pt/TiO_2_ and Y did not alter the crystal structure of either component. Consequently, the Pt/TiO_2_ + Y sample displayed a mixed crystal structure containing both anatase TiO_2_ and zeolite Y. The absence of detectable Pt species in both Pt/TiO_2_ and Pt/TiO_2_ + Y catalysts could be attributed to the low loading of 1 wt.% Pt. After reaction at 300 ^o^C under testing conditions with H_2_O, there are no apparent changes in the crystal structures for both Pt/TiO_2_-*p* and (Pt/TiO_2_ + Y)-*p* catalysts.


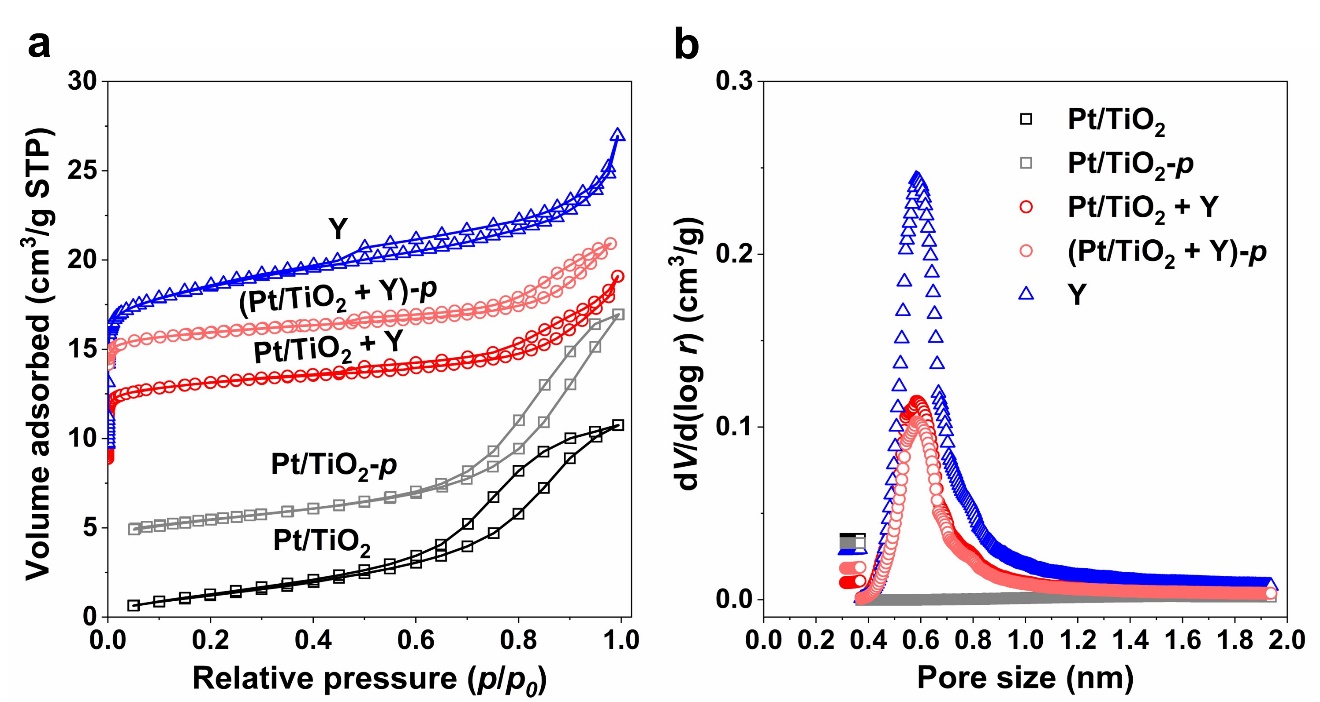


**Supplementary Fig. 10 | Porosity property.** (a) N_2_ adsorption-desorption isotherms, (b) pore size distributions for Pt/TiO_2_, Pt/TiO_2_-*p*, Pt/TiO_2_ + Y, (Pt/TiO_2_ + Y)-*p*, and Y samples. The samples suffixed with “-*p*” represent the sample after reaction at 300 °C under testing conditions with H_2_O.


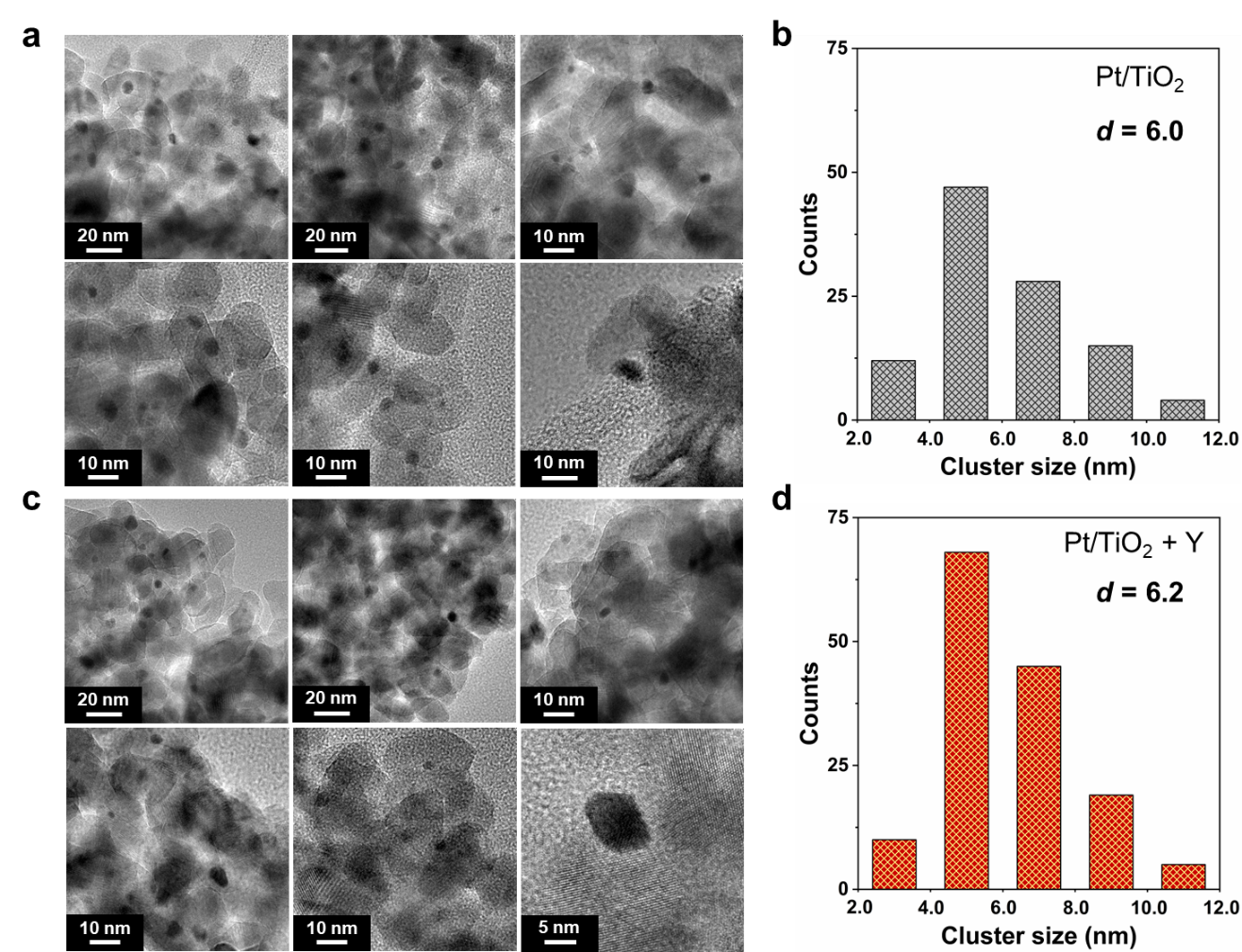


**Supplementary Fig. 11 | Catalyst morphology and Pt particle size.** (a, c) TEM images and (b, d) Pt particle size distribution for (a, b) Pt/TiO_2_ and (c, b) Pt/TiO_2_ + Y catalysts.


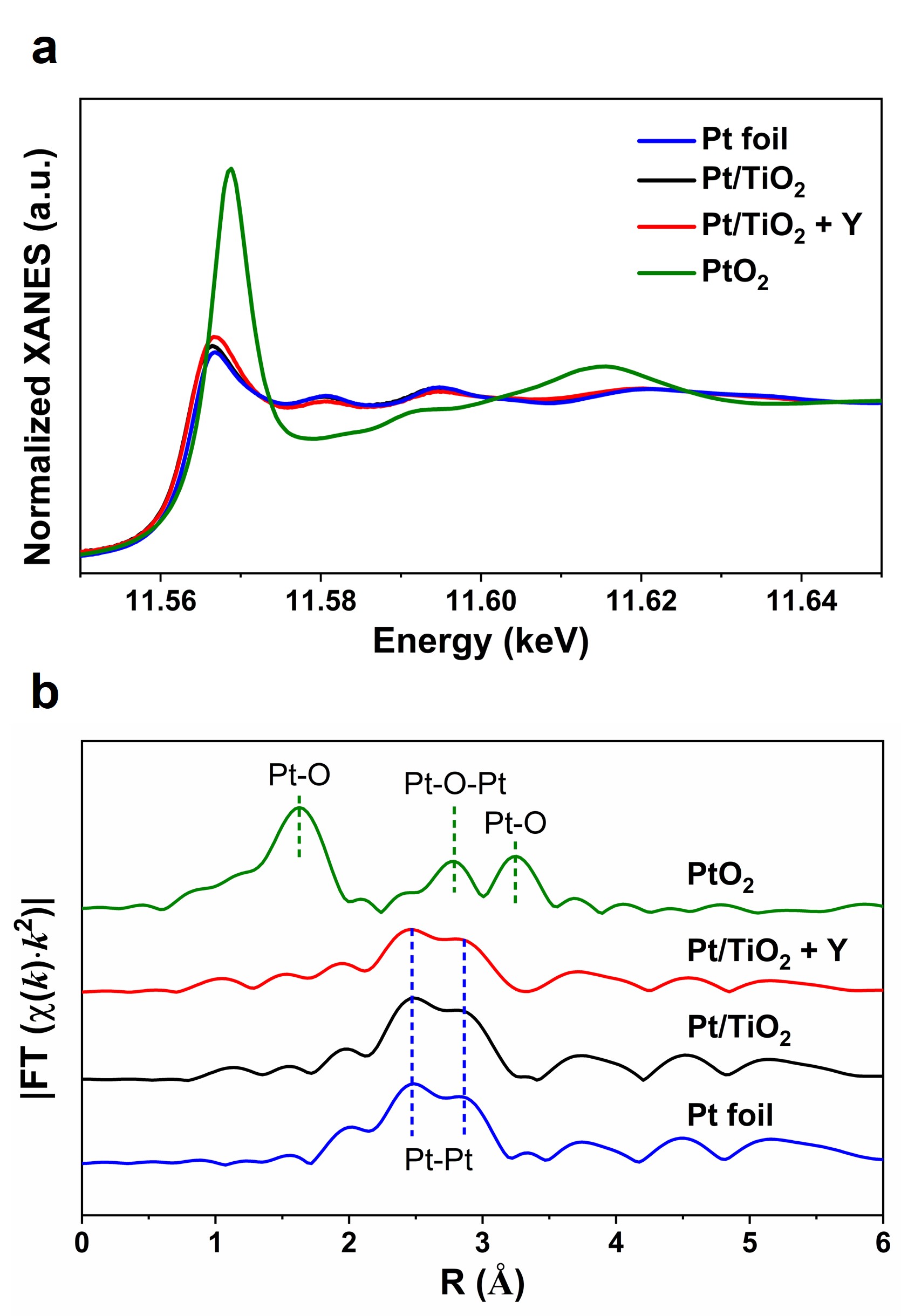


**Supplementary Fig. 12 |** (a) Normalized XANES, and (b) Fourier transformed *k*^2^-weighted EXAFS oscillations in R space for Pt L_3_-edge in Pt/TiO_2_ and Pt/TiO_2_ + Y catalysts, Pt foil and PtO_2_ references.


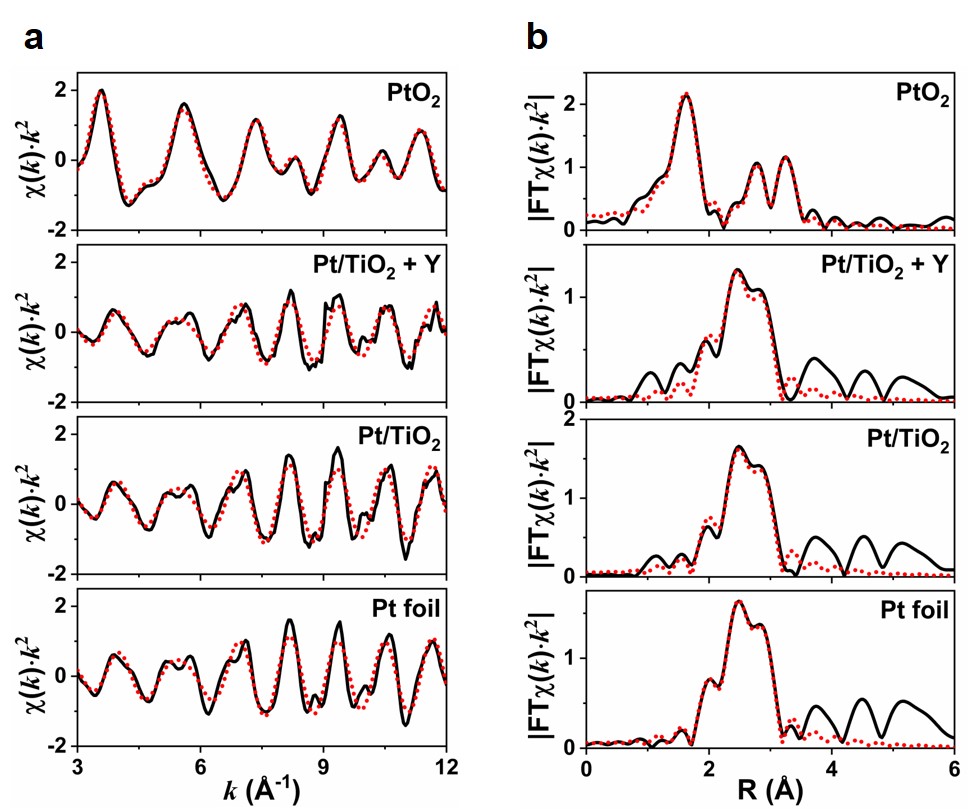


**Supplementary Fig. 13 |** (a) *k*^2^-weighted EXAFS oscillations and (b) Fourier transformed *k*^2^-weighted EXAFS oscillations in R space for Pt L_3_-edge in Pt/TiO_2_ and Pt/TiO_2_ + Y catalysts, Pt foil and PtO_2_ references (solid: experimental data; dotted: fitted data).


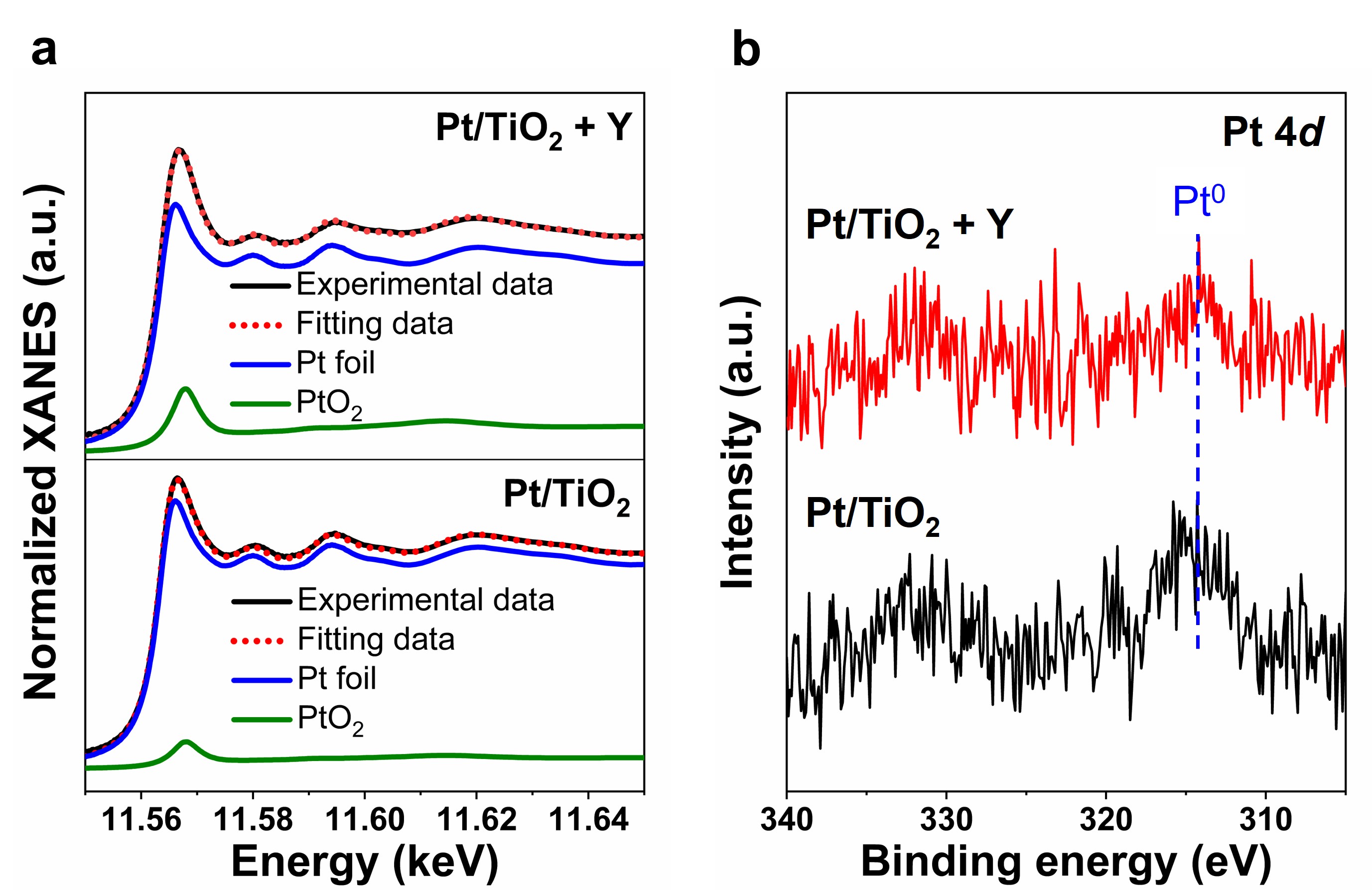


**Supplementary Fig. 14 | Pt oxidation state**. (a) The linear combination fitting results of Pt L_3_-edge XANES, and (b) Pt 4*d* XPS for Pt/TiO_2_ and Pt/TiO_2_ + Y catalysts. The averaged valence states of Pt are presented in **Supplementary Table 4**.


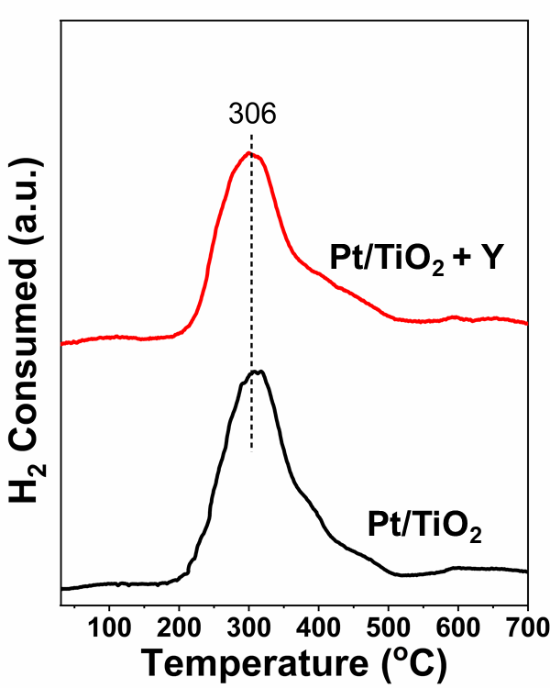


**Supplementary Fig. 15 | Catalyst reducibility.** H_2_-TPR profiles for Pt/TiO_2_ and Pt/TiO_2_ + Y catalysts. The H_2_ consumption signal of Pt/TiO_2_ + Y system was normalized by the amount of Pt/TiO_2_ within the mixture.


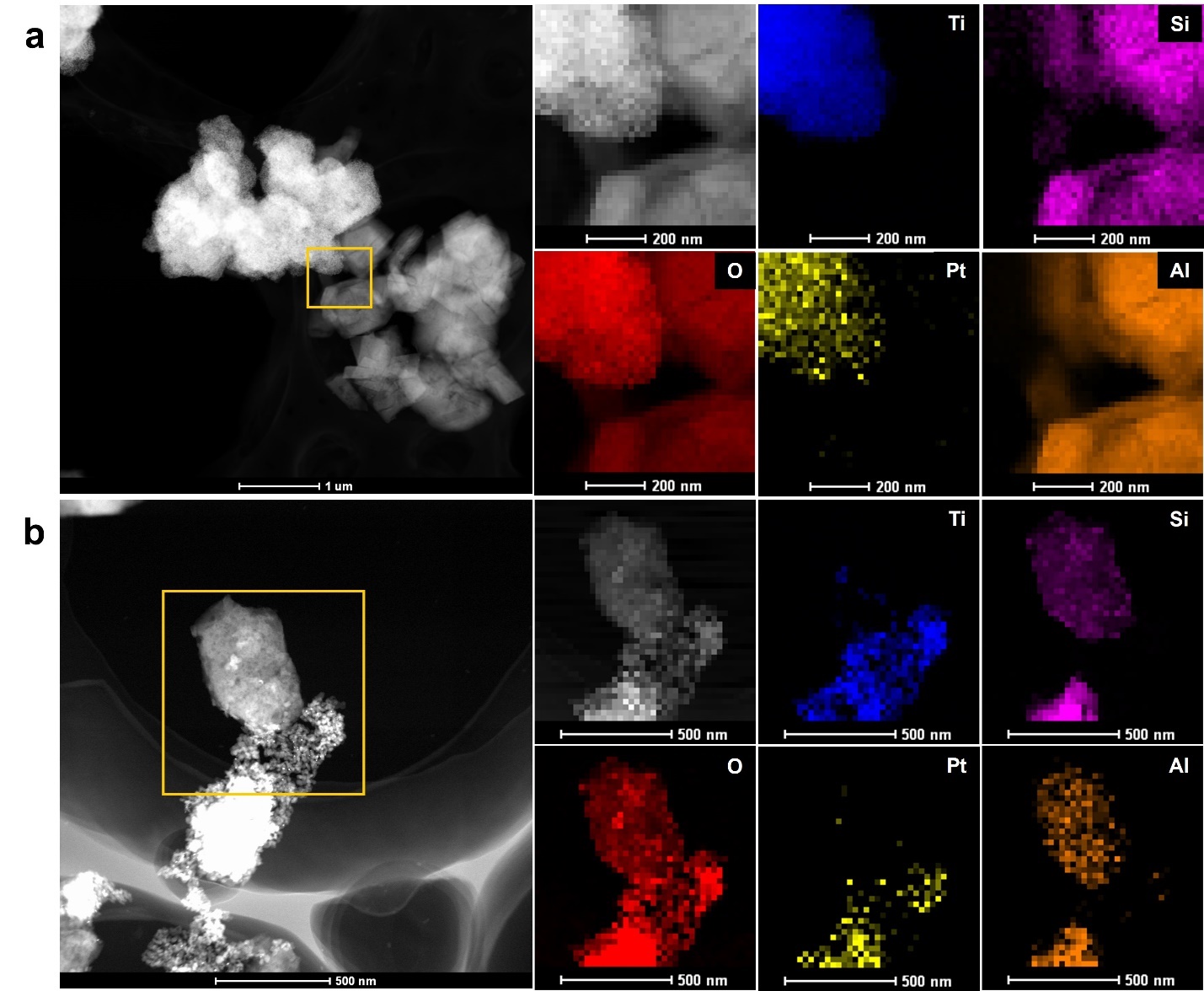


**Supplementary Fig. 16 | Structural characterization of Pt/TiO_2_ + Y.** EDS mapping images for (a) Pt/TiO_2_ + Y and (b) (Pt/TiO_2_ + Y)-*p* samples. The sample suffixed with “-*p*” represents the sample after reaction at 300 °C under testing conditions with H_2_O.


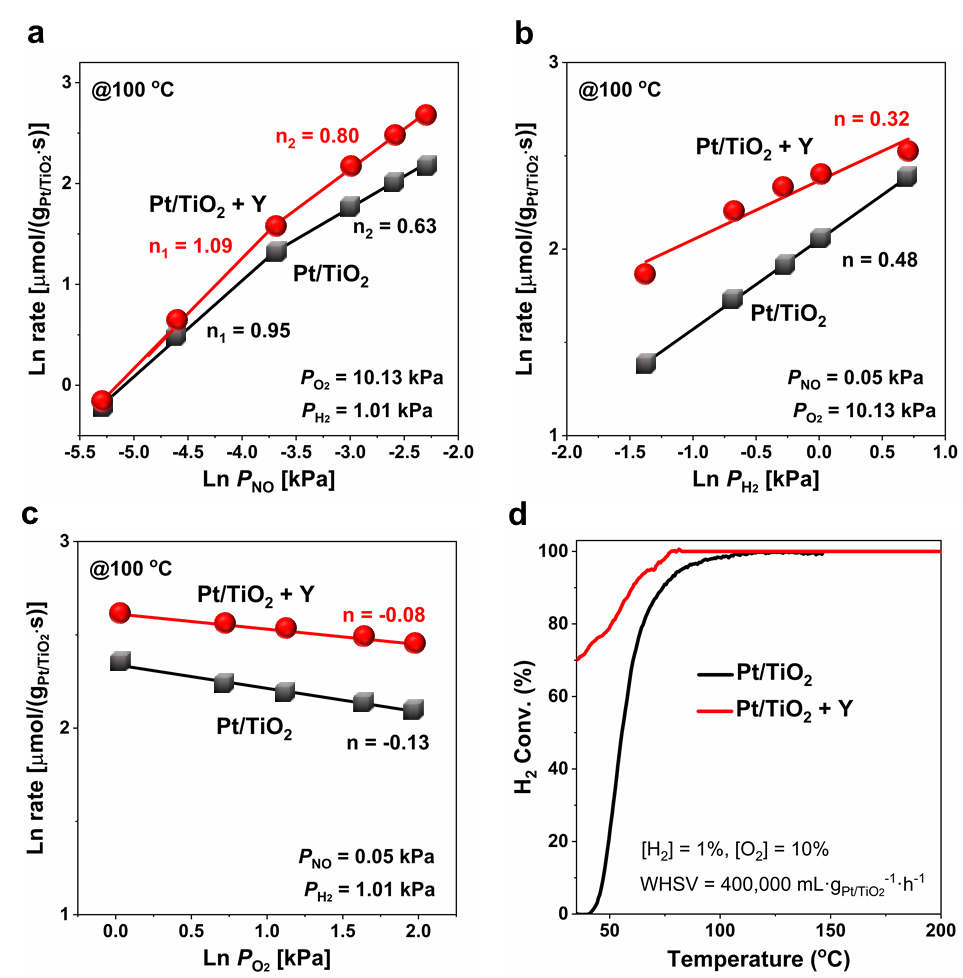


**Supplementary Fig. 17 | Kinetics study for H_2_-SCR reaction and H_2_ oxidation performance**. (a) NO, (b) H_2_, and (c) O_2_ reaction orders in the H_2_-SCR of NO; and (d) H_2_ oxidation activity on Pt/TiO_2_ and Pt/TiO_2_ + Y catalysts. Kinetics study was performed at 100 ^o^C under WHSV of 2,400,000 mL·g_Pt/TiO2_^–1^·h^–1^.





**Supplementary Fig. 18 | H_2_ oxidation performance**. H_2_ oxidation activity on Pt/TiO_2_, Pt/TiO_2_ + Y-50%, and Pt/TiO_2_ + Y-67% catalysts.


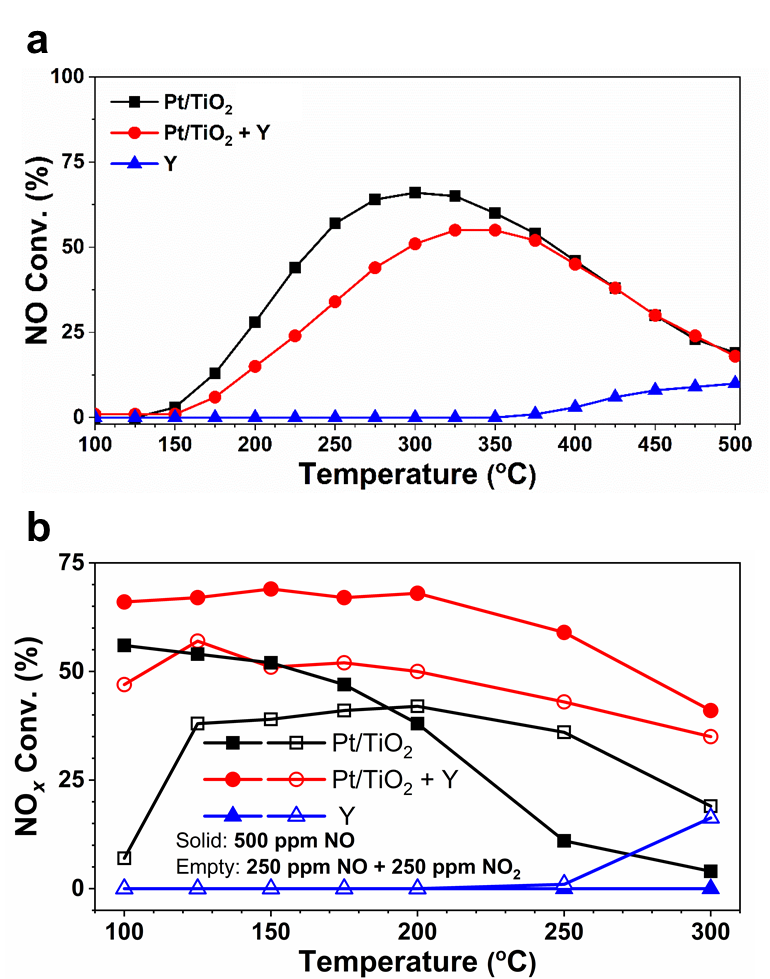


**Supplementary Fig. 19 | NO oxidation performance and effect of NO_2_ on the H_2_-SCR activity**. (a) NO oxidation activity on Pt/TiO_2_, Pt/TiO_2_ + Y, and Y samples; (b) Effect of NO_2_ addition into the H_2_-SCR flow on the NO*_x_* reduction activity of Pt/TiO_2_, Pt/TiO_2_ + Y, and Y samples. NO oxidation conditions: 30 mg of Pt/TiO_2_ or Y, or a mixture containing 30 mg of Pt/TiO_2_ and 30 mg of Y; 500 ppm NO, and 10% O_2_; WHSV = 400,000 mL·g_Pt/TiO2_^–1^·h^–1^. H_2_-SCR reaction conditions with NO_2_: 26 mg of Pt/TiO_2_ or Y, or a mixture containing 26 mg of Pt/TiO_2_ and 26 mg of Y; 250 ppm NO, 250 ppm NO_2_, 1% H_2_, 10% O_2_, 5% CO_2_, and 5% H_2_O; WHSV = 461,540 mL·g_Pt/oxide_^–1^·h^–1^.

**Notes**: As demonstrated in **Fig. 1c** in the main text, the physical mixing with Y effectively inhibited the NO selective oxidation to NO_2_ on Pt/TiO_2_ catalyst during the H_2_-SCR reaction. This observation was further supported by the separate NO oxidation testing, in which Pt/TiO_2_ + Y system indeed displayed noticeably lower NO oxidation activity comparing to Pt/TiO_2_ (**Supplementary Fig. 19a**). Since NO_2_ is usually present in the reaction atmosphere, the effect of NO_2_ on H_2_-SCR activity was investigated by introducing NO_2_ to replace half of the NO (**Supplementary Fig. 19b**). Consistent with the previous observations in literature showing the negative impact of NO_2_ on H_2_-SCR activity,^1,2^ our results also proved that the presence of NO_2_ drastically decreased the low-temperature NO*_x_* conversion on both Pt/TiO_2_ and Pt/TiO_2_ + Y. Therefore, it can be concluded that the promotion of H_2_-SCR activity on Pt/TiO_2_ + Y was not due to the enhanced NO oxidation (which was indeed favorable in other catalytic NO*_x_* reduction process such as by NH_3_).


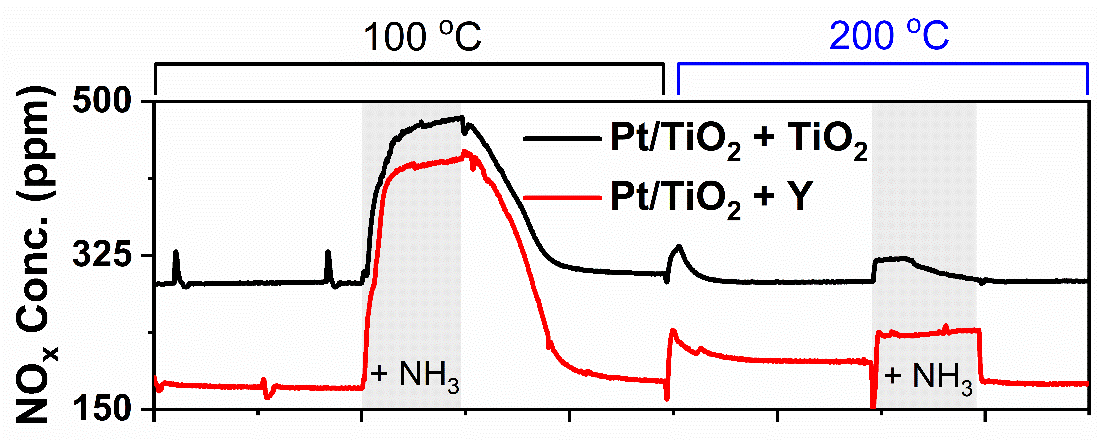

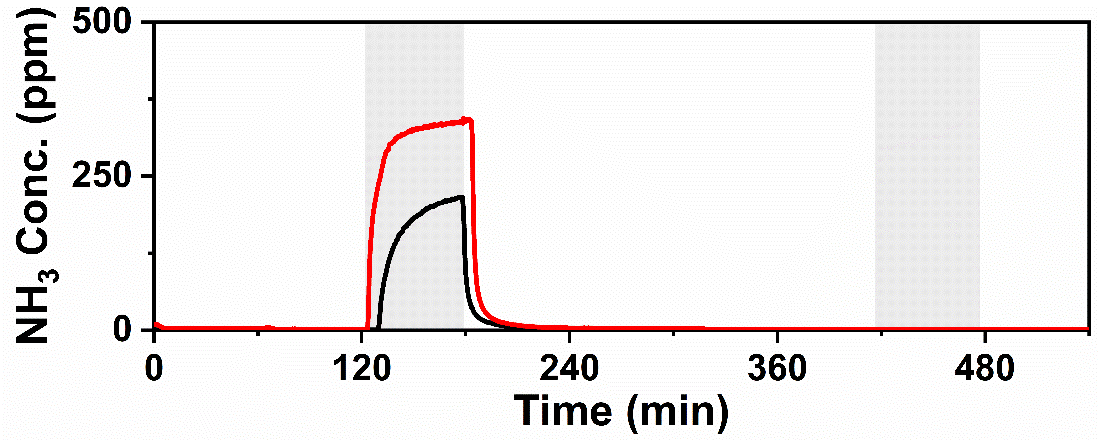


**Supplementary Fig. 20 | Effect of NH_3_ addition on the H_2_-SCR activity on Pt/TiO_2_ and Pt/TiO_2_ + Y catalysts at 100 and 200 ^o^C**. Reaction conditions: 10 mg of Pt/TiO_2_, or a mixture containing 10 mg of Pt/TiO_2_ and 10 mg of Y; 500 ppm NO, 1% H_2_, 500 ppm NH_3_ (if used), 10% O_2_, and 5% H_2_O; WHSV = 1,200,000 mL·g_Pt/TiO2_^–1^·h^–1^.

**Notes**: To investigate the effect of NH*_x_* species (possibly formed through the *in situ* reduction of NO*_x_* by H_2_) on H_2_-SCR performance by potentially facilitating the NH_3_-SCR reaction, the time-resolved NO*_x_* concentration during the H_2_-SCR reaction over Pt/TiO_2_ and Pt/TiO_2_ + Y with NH_3_ addition at 100 and 200 °C was collected (**Supplementary Fig. 20**). Different from the positive effect of NH_3_ addition on H_2_-SCR activity observed on the reported Pt catalysts such as Pt-MnO*_x_* and Pt/ZSM-35,^3-5^ the introduction of NH_3_ led to increased NO*_x_* concentration (*i.e.*, lowered H_2_-SCR activity) over both Pt/TiO_2_ and Pt/TiO_2_ + Y in this study. In particular, the NO*_x_* concentration in the reaction flow over Pt/TiO_2_ + Y system exhibited a more significant rise comparing to that over Pt/TiO_2_, especially at 100 ^o^C. These results evidently suggest that the promotion effect from physically mixing Pt/TiO_2_ with Y was not attributed to the facilitation of *in situ* NH_3_-SCR reaction.


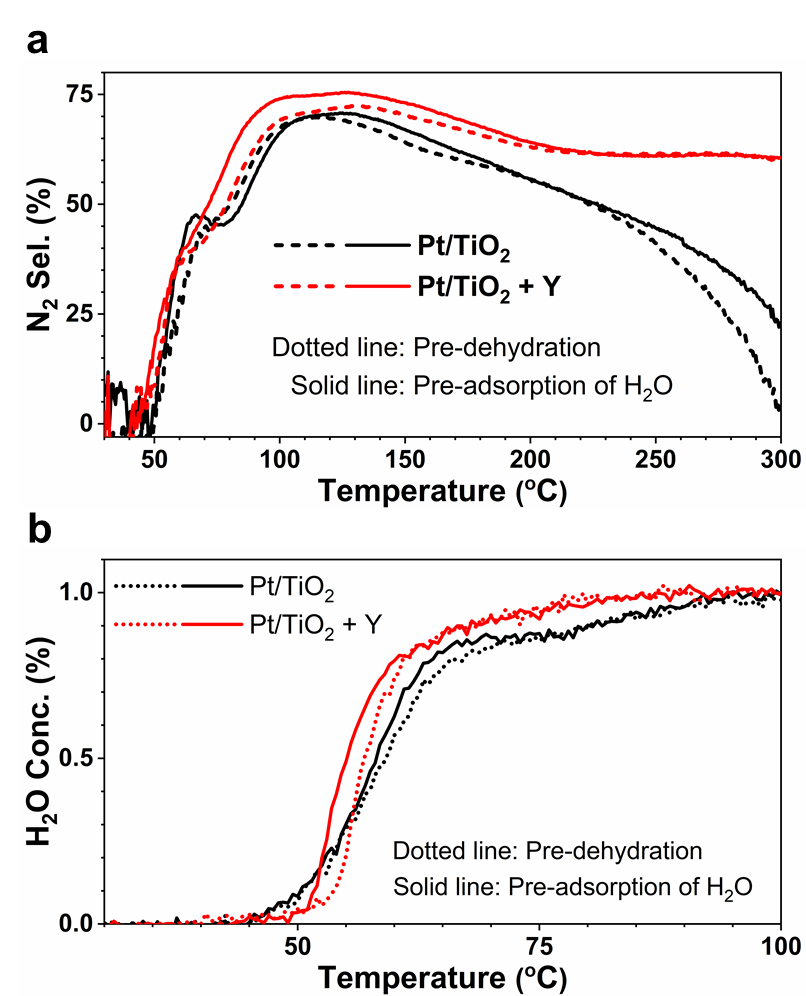


**Supplementary Fig. 21 | Effect of H_2_O on the H_2_-SCR performance.** (**a**) N_2_ selectivity and (**b**) gas phase H_2_O formation during the H_2_-SCR reaction over Pt/TiO_2_ and Pt/TiO_2_ + Y catalysts with pre-dehydration at 300 ^o^C or pre-adsorption of H_2_O at 30 ^o^C. Reaction conditions: 26 mg of Pt/TiO_2_ catalyst, or a mixture containing 26 mg of Pt/TiO_2_ and 26 mg of Y; transient state light-off testing; 500 ppm NO, 1% H_2_, and 10% O_2_; WHSV = 461,540 mL·g_Pt/TiO2_^–1^·h^–1^.


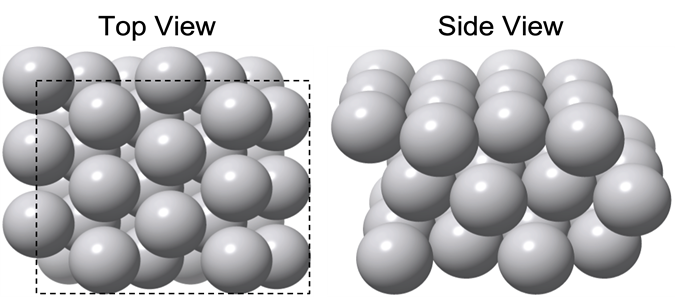


**Supplementary Fig.** **22 | Top and side views of the DFT-optimized Pt (111) facet.** The periodic supercell in the x and y directions is indicated by the black dashed line.

**Notes:** As the Pt (111) facet has the highest thermodynamic stability and is likely to be the most abundant surface exposed on Pt nanoparticles, it was chosen as the model system for Pt/TiO_2_ catalyst and modeled by a $2\sqrt{3}\times3$ supercell with three atomic layers and 36 Pt atoms, as shown in **Supplementary Fig. 22**. A vacuum gap of ~20 Å was employed to avoid the artificial interaction between periodic slabs. In this slab model, the bottom layer was fixed while the top two layers and adsorbates were allowed to relax until the forces and energies converged.


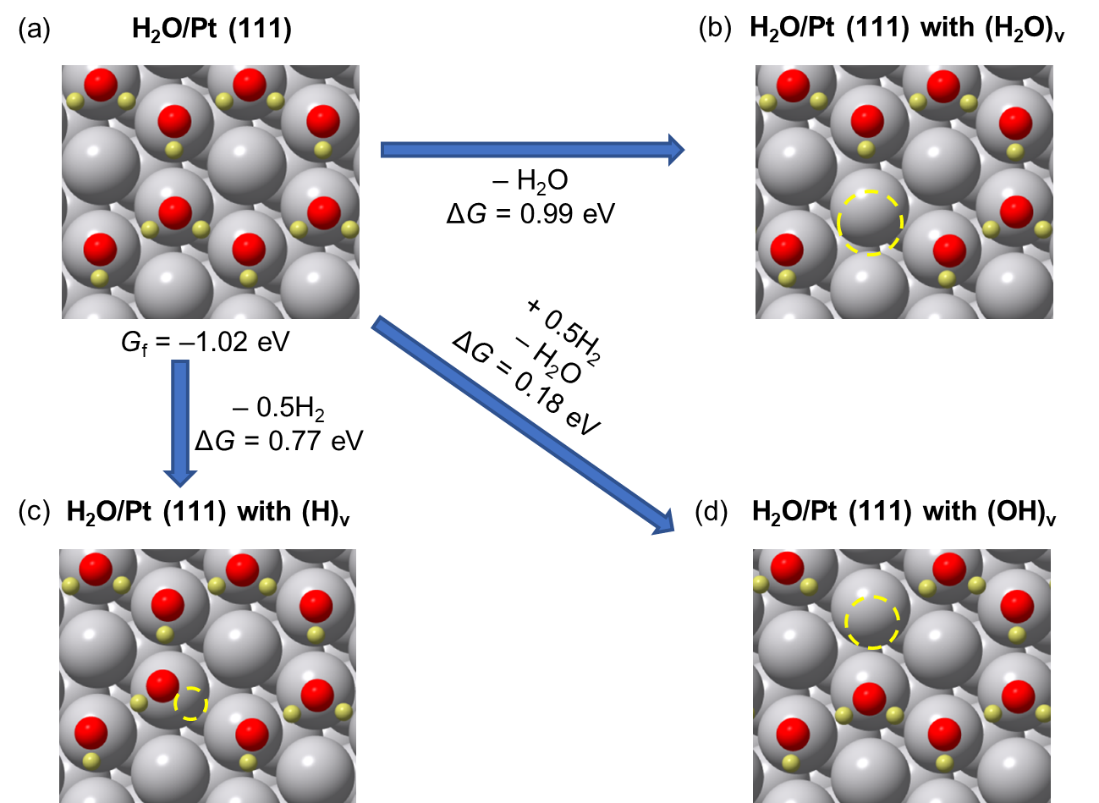


**Supplementary Fig. 23 | Stability of the H_2_O/Pt (111) structure.** (H)_v_, (OH)_v_, and (H_2_O)_v_ represent H, OH, and H_2_O vacancies, respectively, in the hydrogen bonding network of the H_2_O/Pt (111). The yellow dashed circles represent the positions of the corresponding vacancies. It was thermodynamically unfavorable to create a vacancy of H, OH or H_2_O in the hydrogen bonding network of the H_2_O/Pt (111) because of free energy uphill, which together with the high free formation energy of the H_2_O/Pt (111) in (a) suggested its high stability.

**Notes:** On the Pt/TiO_2_ + Y system, the zeolite Y could create a H_2_O-rich local environment around Pt sites (see **Fig. 3e** in the main text and relevant discussion), fostering the formation of H_2_O layers on the surface of Pt nanoparticles. Under the specific aqueous micro-environment, the most stable H_2_O structure on the Pt (111) surface formed a honeycomb ($\sqrt{3}\times\sqrt{3}$)R30 pattern with 2/3 ML coverage, which has been proven both experimentally and theoretically.^11,12^ It is noteworthy that the adsorbed *NO could easily react with H from *H_2_O in the water layer, creating a more stable hydrogen bonding network with half water dissociated (labeled as H_2_O/Pt (111), as shown in **Supplementary Fig. 23a**).^13^ The high stability of the H_2_O/Pt (111) was further confirmed by the computational results in **Supplementary Fig. 23**. Therefore, the H_2_O/Pt (111) surface was chosen as the model structure representing the Pt/TiO_2_ + Y catalyst system.


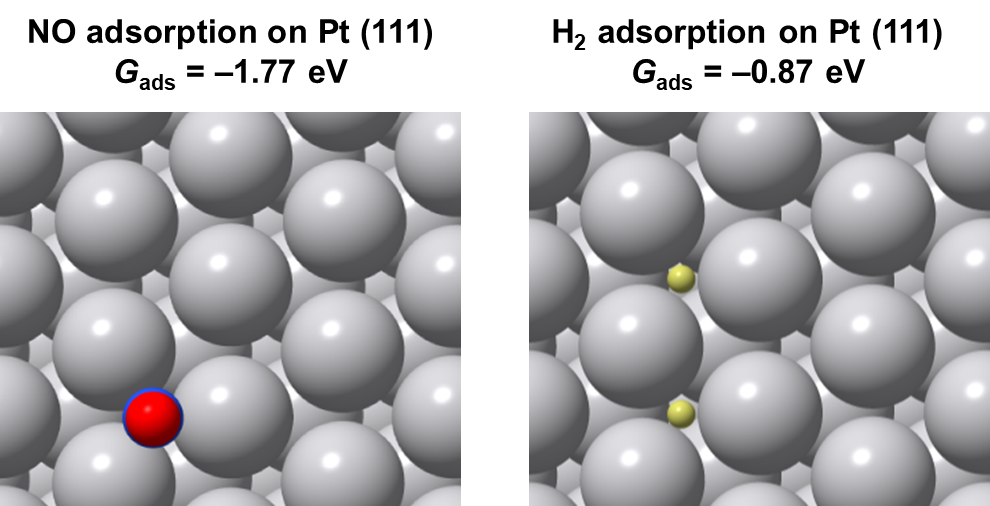


**Supplementary Fig. 24 | NO and H_2_ adsorption on Pt (111) surface.**


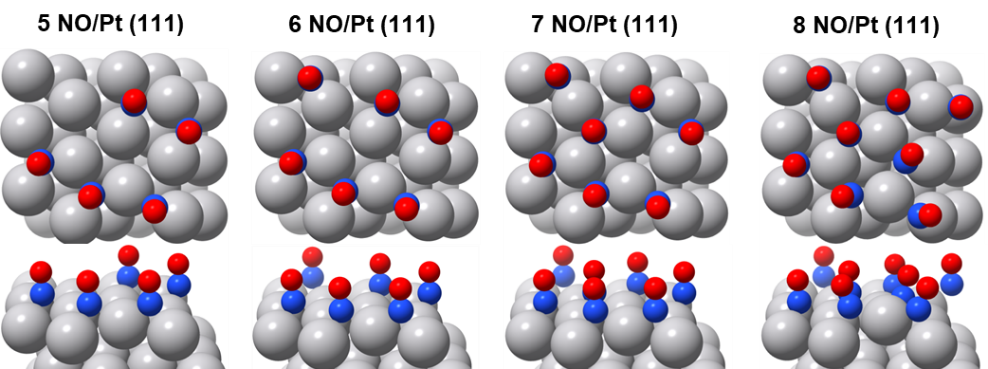


**Supplementary Fig. 25 | Optimized configurations of NO adsorption at varying coverage on a 2**$\sqrt{\boldsymbol{3}}$ **✕ 3 supercell of Pt (111).** 5 NO/Pt (111), 6 NO/Pt (111), 7 NO/Pt (111), and 8 NO/Pt (111) are equivalent to the *NO coverages of 5/12, 1/2, 7/12, and 3/4 ML on the Pt (111) surface, respectively. Color code: Pt (silver), O (red), and N (blue).


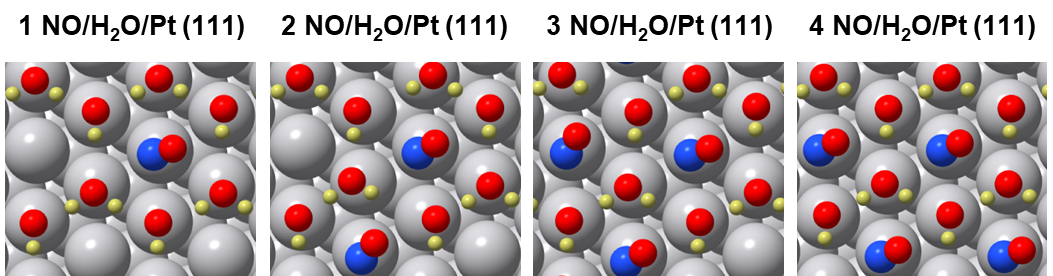


**Supplementary Fig. 26 | Optimized configurations of NO adsorption at varying coverage on the H_2_O/Pt (111).** 1 NO/H_2_O/Pt (111), 2 NO/H_2_O/Pt (111), 3 NO/H_2_O/Pt (111), and 4 NO/H_2_O/Pt (111) are equivalent to the *NO coverages of 1/12, 1/6, 1/4, and 1/3 ML on the H_2_O/Pt (111) surface, respectively. Color code: Pt (silver), O (red), N (blue), and H (yellow).


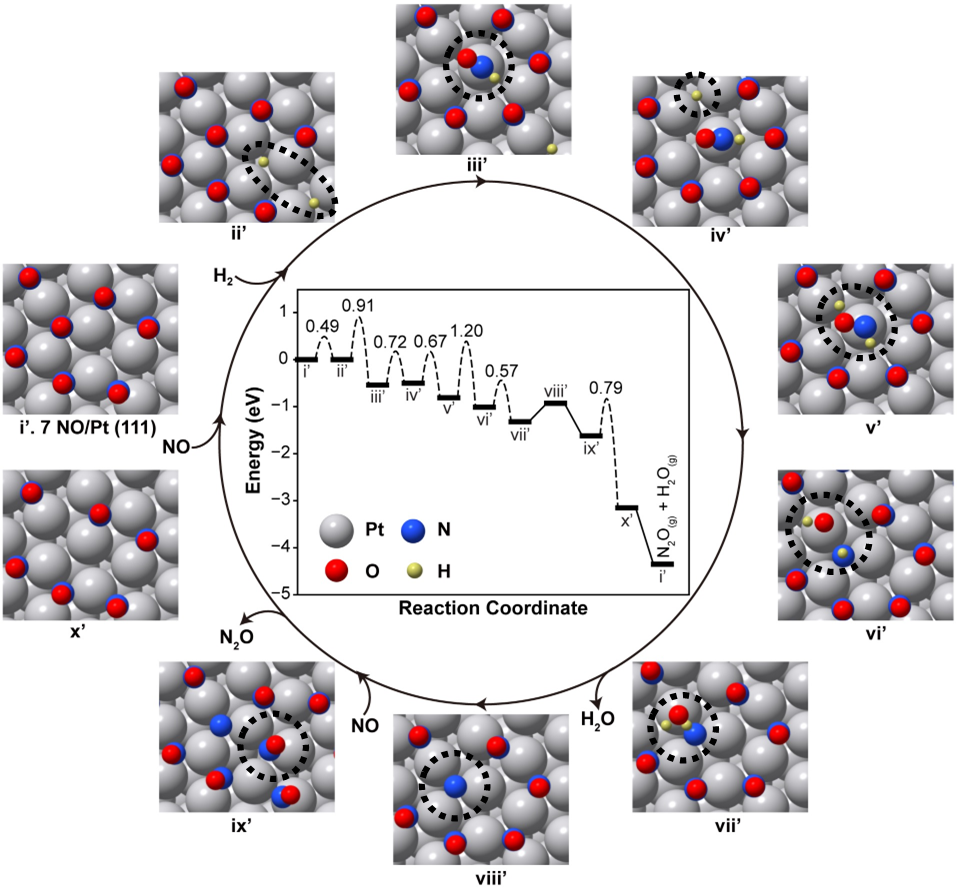


**Supplementary Fig. 27 | Potential energy diagrams and configurations for the H_2_-SCR cycle on the Pt/TiO_2_ catalyst.** The reaction was proposed to proceed on the 7 NO/Pt (111) surface representing the structure of Pt/TiO_2_ catalyst under reaction conditions. The reaction energies and activation energies are indicated in eV in the diagram. Color code: Pt (silver), O (red), N (blue), and H (yellow). Corresponding energies of all intermediates are given in **Supplementary Table 5**.

**Notes:** Due to the high thermodynamic stability under experimental operation conditions (**Figure 5a** in the main text), the 7 NO/Pt (111) structure was assigned as the starting point for studying the H_2_-SCR cycle on Pt/TiO_2_ catalyst. As shown in **Supplementary Fig. 27** and **Supplementary Table 5**, the reaction was initiated with the H_2_ adsorption onto the 7 NO/Pt (111) surface, which generated two atomic *H with an activation barrier of 0.49 eV (image i’ to ii’). Then, one of the *H atoms attacked one of *NO adsorbates and generated *HNO species on the Pt top site with an activation energy of 0.91 eV (image ii’ to iii’). Afterwards, the residual *H diffused readily across the surface and further reduced the *HNO species to *HNOH with an overall barrier of 0.72 eV (image iii’ to v’). As the further reduction of *HNOH species was unfavorable, it preferred to dissociate into *NH and *OH species with a significant activation barrier of 1.20 eV (image v’ to vi’). Subsequently, the *OH species extracted the H from the nearby *NH species with a small activation energy of 0.57 eV, resulting in the formation of a physisorbed *H_2_O and an *N species (image vi’ to vii’). Following the H_2_O desorption, a gas phase NO could readily occupy an empty hollow site on the Pt surface and subsequently react with the *N species, involving a moderate activation barrier of 0.79 eV (image viii’ to x’). This step generated and released a N_2_O to the gas atmosphere (image ix’ to x’). Interestingly, the residual 6 *NO on the Pt surface were found inert and severed as the ‘spectators’ during the NO reduction reaction (image x’). To complete the reaction cycle, another gas phase NO could incorporate into the 6NO/Pt and renew the catalyst surface (image x’ to i’). It was worth noting that the surface elementary steps involving the spectator *NO were less favorable than their competing steps within the proposed H_2_-SCR mechanism, suggesting that the strong Pt-NO bonding fundamentally poisoned the Pt (111) surface by blocking the Pt sites to activate H_2_. Furthermore, owing to the unfavorable H_2_ dissociation (**Fig. 5b** in the main manuscript), the *H species were in shortage during the catalytic cycle and thus hindered the further reduction of N-containing species to N_2_. This resulted in the high selectivity towards N_2_O, in line with the low N_2_ selectivity observed in experiments (**Fig. 1b** in the main manuscript). Overall, the dissociation of *HNOH species was the rate-determining step (RDS) for the whole NO reduction cycle on the Pt (111) surface representing Pt/TiO_2_ catalyst with an activation energy of 1.20 eV.

On the H_2_O/Pt (111) surface representing the Pt/TiO_2_ + Y catalyst, as shown in **Fig. 6** in the main manuscript and **Supplementary Table 6**, the H_2_-SCR reaction started with the facile adsorption of H_2_ and NO (image i to iii). The interstitial *H from gas phase H_2_ dissociation, with the lowest H binding energy (**Supplementary Fig. 28**), reacted readily with the *NO and generated a *HNO, involving an activation barrier of 0.58 eV (image iii to iv). Afterwards, the residual *H atom attacked an *OH in the hydrogen bonding network with an apparent activation energy of 0.47 eV, yielding a *H_2_O (image iv to v). Following the H_2_O desorption, a vacancy was created within the hydrogen bonding network, where the gas phase H_2_ could easily dissociate into two *H (image v to vii). Then, the *HNO went through the further reduction by *H to form *HNOH with an activation barrier of only 0.40 eV (image vii to viii). In contrast to the situation on Pt (111) surface, the *HNOH species on the H_2_O/Pt (111) surface readily dissociated into *NH and *OH (*E*_a_ = 0.24 eV from image viii to ix), restoring the hydrogen bonding network. Such low activation barrier could be attributed to the presence of hydrogen bonding network which stabilized the transition state of the *HNOH dissociation. Once the *NH was formed, the gas phase NO could couple with the *NH to generate *HNNO, involving a substantial exothermicity of 2.31 eV (image ix to x). Interestingly, rather than releasing a N_2_O (*E*_a_ = 1.23 eV from image x to ii), the *HNNO stayed on the surface until an OH vacancy was created in the hydrogen bonding network following the H_2_O formation (*E*_a_ = 0.40 eV from image x to xi) and desorption (Δ*E* = 0.32 eV from image xi to xii). The *HNNO species was activated by the vacancy and dissociated into a gas phase N_2_, an interstitial *H, and an O in the hydrogen bonding network with an activation barrier of 0.54 eV (image xii to xiii). Finally, the H_2_O/Pt (111) surface was restored after the *H diffusion from the interstitial site to the hydrogen bonding network. Therefore, the RDS of NO reduction on the H_2_O/Pt (111) surface included both the creation of an OH vacancy in the hydrogen bonding network and N_2_ formation with an overall activation energy of 0.86 eV (from image xi to xiii). Such activation energy for the RDS in the H_2_-SCR of NO on the H_2_O/Pt (111) surface representing the Pt/TiO_2_ + Y system (*E*_a_ = 0.86 eV) was confirmed much lower than that on the Pt (111) surface representing the Pt/TiO_2_ catalyst (*E*_a_ = 1.20 eV).


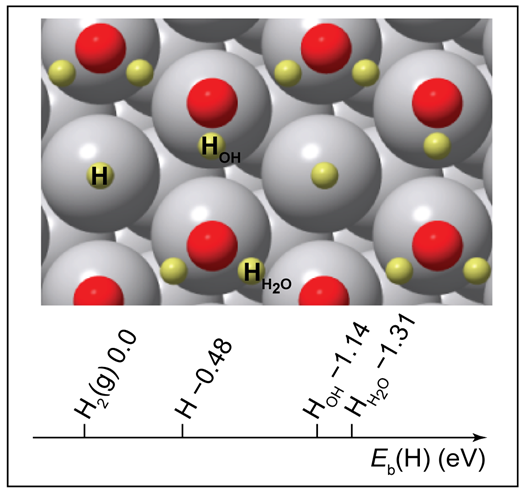


**Supplementary Fig. 28 | Binding energy of H species on the H_2_O/Pt (111) surface**. The *H species at the interstitial site from gas phase H_2_ dissociation showed the lowest binding energy (–0.48 eV), indicating that such *H species should have directly involved in the NO reduction process. Color code: Pt (silver), O (red), and H (yellow).

**Supplementary References**

1 Wu, P., Li, L., Yu, Q., Wu, G. & Guan, N. Study on Pt/Al-MCM-41 for NO selective reduction by hydrogen. *Catalysis Today* **158**, 228-234 (2010).

2 Zhang, X. *et al.* An investigation on N_2_O formation route over Pt/HY in H_2_-SCR. *Chemical Engineering Journal* **252**, 288-297 (2014).

3 Park, D. C. *et al.* Widening the operating window of Pt/ZSM-5 catalysts for efficient NO*_x_* removal in H_2_-SCR: Insights from thermal aging. *Catalysis Today* **425** (2024).

4 Kalamaras, C. M., Olympiou, G. G., Pârvulescu, V. I., Cojocaru, B. & Efstathiou, A. M. Selective catalytic reduction of NO by H_2_/C_3_H_6_ over Pt/Ce_1-_*_x_*Zr*_x_*O_2-_*_δ_*: The synergy effect studied by transient techniques. *Applied Catalysis B: Environmental* **206**, 308-318 (2017).

5 Liu, Z. *et al.* Selective catalytic reduction of NO*_x_* with H_2_ over WO_3_ promoted Pt/TiO_2_ catalyst. *Applied Catalysis B: Environmental* **188**, 189-197 (2016).

6 Borchers, M., Lott, P. & Deutschmann, O. Selective Catalytic Reduction with Hydrogen for Exhaust gas after-treatment of Hydrogen Combustion Engines. *Topics in Catalysis* **66**, 973-984 (2022).

7 Patel, V. K. & Sharma, S. Effect of oxide supports on palladium based catalysts for NO reduction by H_2_-SCR. *Catalysis Today* **375**, 591-600 (2021).

8 Zhang, Y., Zeng, H., Jia, B., Wang, Z. & Liu, Z. Selective catalytic reduction of NO by H_2_ over Pd/TiO_2_ catalyst. *Chinese Journal of Catalysis* **40**, 849-855 (2019).

9 Hu, Z., Yong, X., Li, D. & Yang, R. T. Synergism between palladium and nickel on Pd-Ni/TiO_2_ for H_2_-SCR: A transient DRIFTS study. *Journal of Catalysis* **381**, 204-214 (2020).

10 Xie, S. *et al.* Silica modulated palladium catalyst with superior activity for the selective catalytic reduction of nitrogen oxides with hydrogen. *Applied Catalysis B: Environmental* **327** (2023).

11 Tripković, V., Skúlason, E., Siahrostami, S., Nørskov, J. K. & Rossmeisl, J. The oxygen reduction reaction mechanism on Pt(111) from density functional theory calculations. *Electrochimica Acta* **55**, 7975-7981 (2010).

12 Ogasawara, H. *et al.* Structure and bonding of water on Pt(111). *Phys Rev Lett* **89**, 276102 (2002).

13 Hansen, H. A., Viswanathan, V. & Nørskov, J. K. Unifying Kinetic and Thermodynamic Analysis of 2 e– and 4 e– Reduction of Oxygen on Metal Surfaces. *The Journal of Physical Chemistry C* **118**, 6706-6718 (2014).
