# Supplementary material for: Zeolite-promoted platinum catalyst for efficient reduction of nitrogen oxides with hydrogen
Source: Nat Commun. 2024 Sep 12;15:7988. doi: 10.1038/s41467-024-52382-7 (PMC11405393; doi:10.1038/s41467-024-52382-7)
Supplement: Supplementary file 4 — Source Data [file 41467_2024_52382_MOESM4_ESM.zip › Source Data/Source data-Main Manuscript.pptx]

## Slide 1
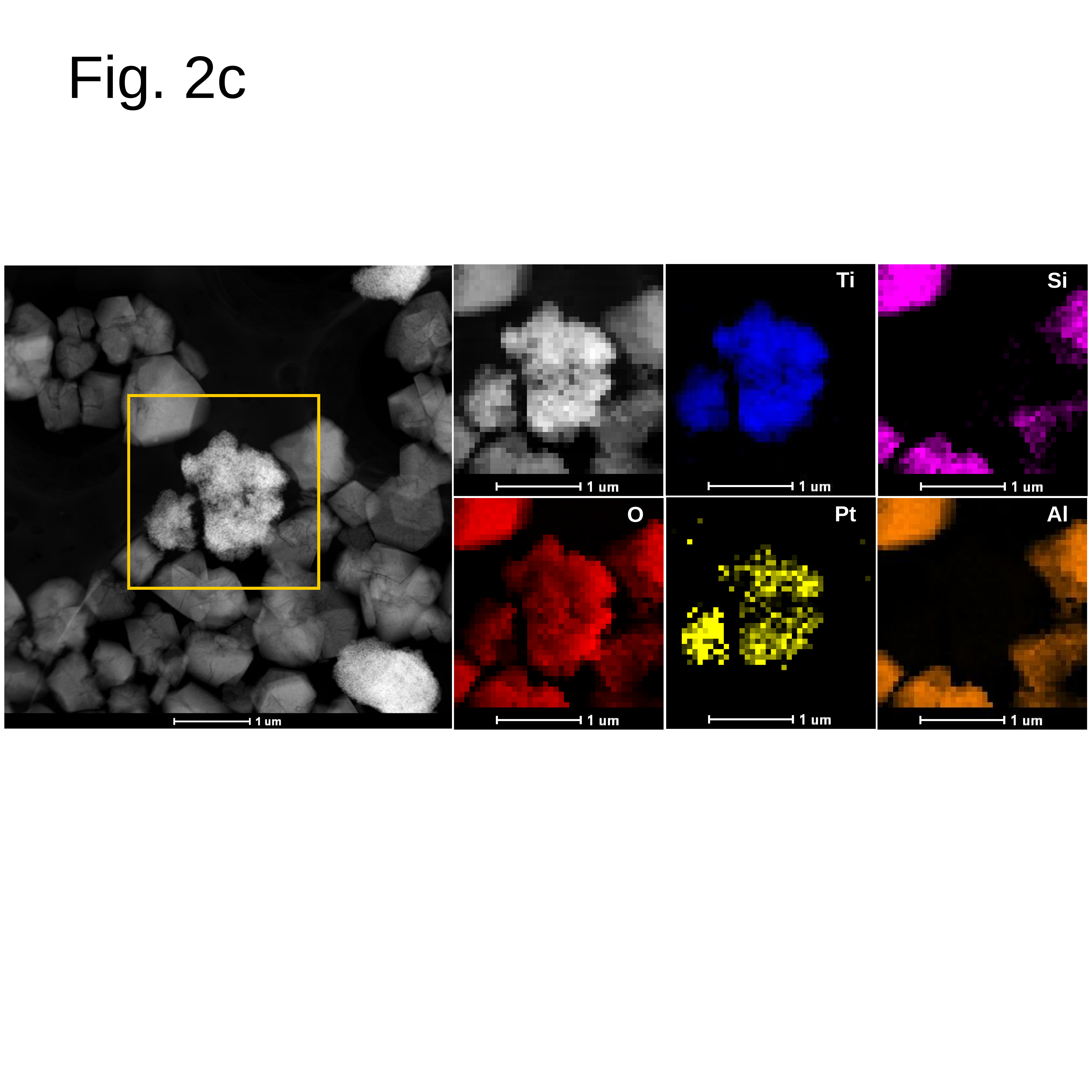

Fig. 2c
Ti
Si
O
Pt
Al

## Slide 2
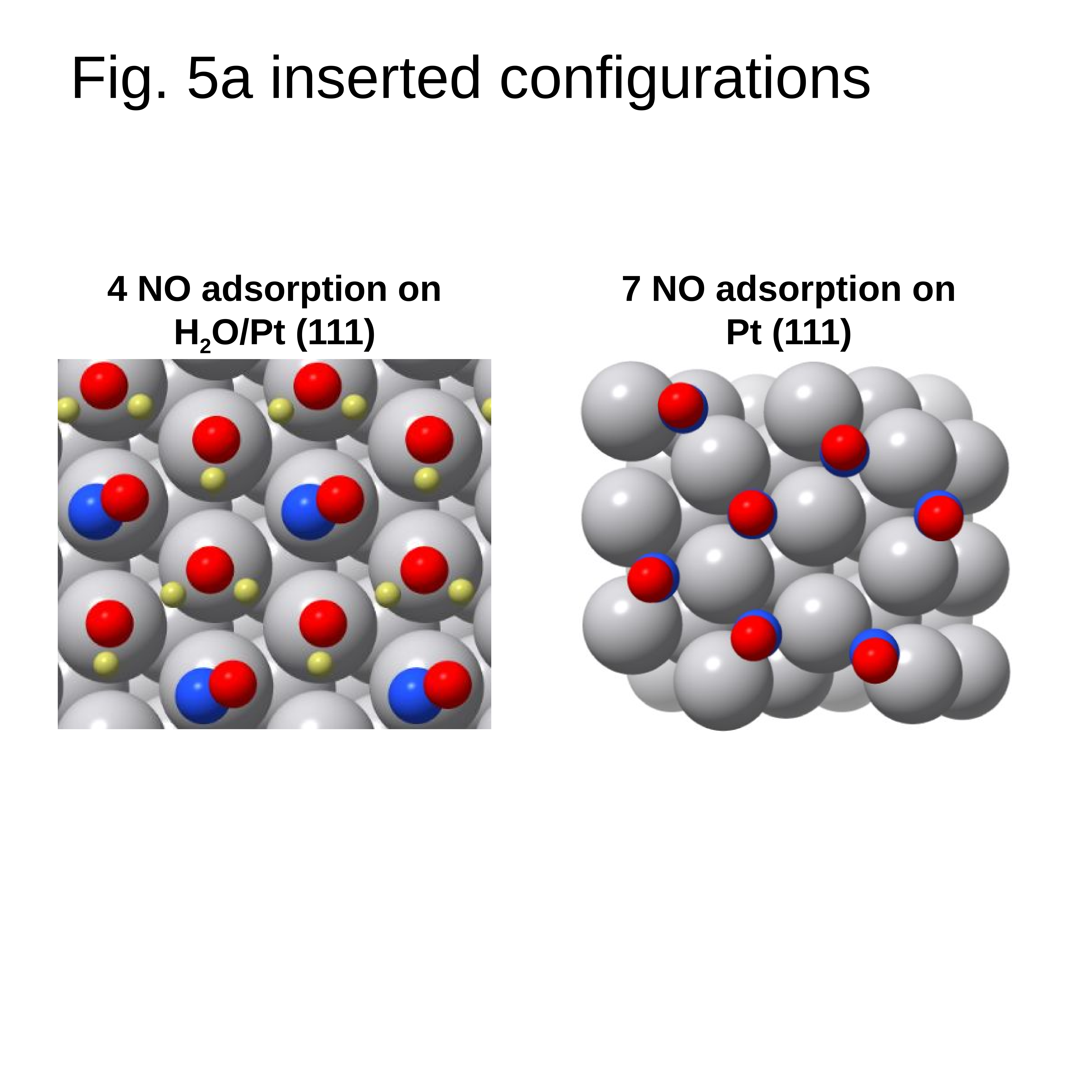

Fig. 5a inserted configurations
4 NO adsorption on H2O/Pt (111)
7 NO adsorption on Pt (111)

## Slide 3
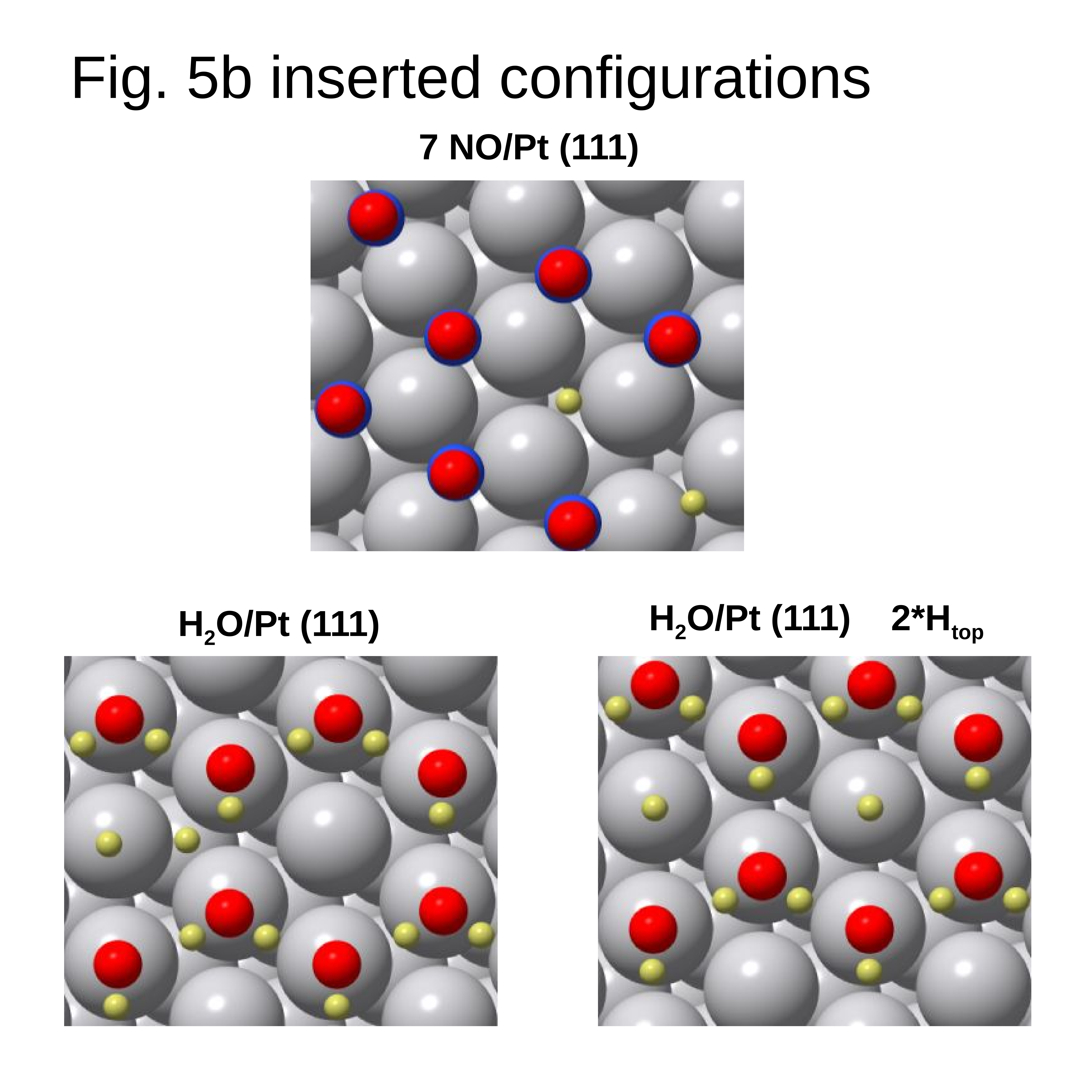

Fig. 5b inserted configurations
7 NO/Pt (111)
H2O/Pt (111) 2*Htop
H2O/Pt (111)

## Slide 4
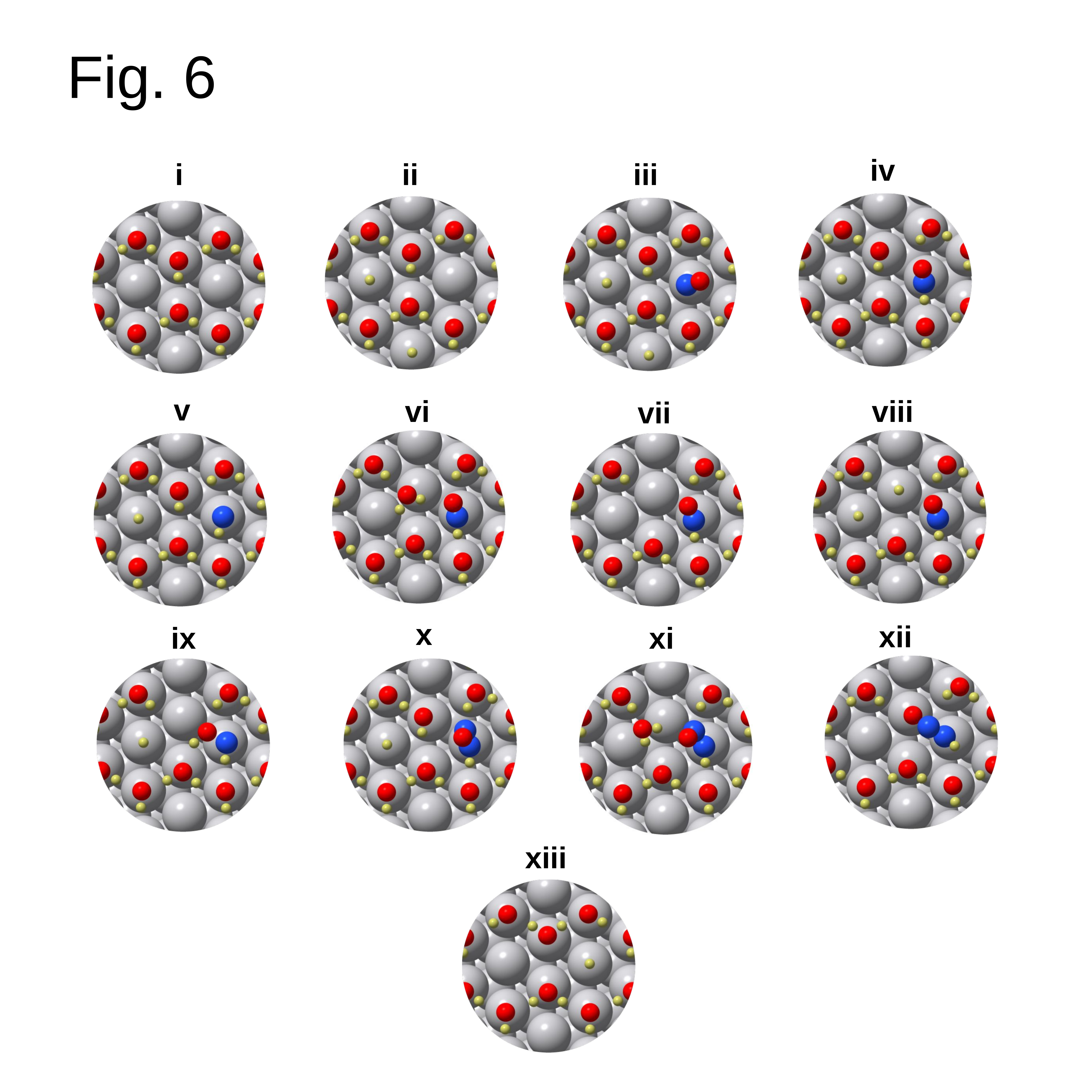

Fig. 6
iv
i
ii
iii
v
vi
viii
vii
x
xii
ix
xi
xiii
