# Supplementary material for: Zeolite-promoted platinum catalyst for efficient reduction of nitrogen oxides with hydrogen
Source: Nat Commun. 2024 Sep 12;15:7988. doi: 10.1038/s41467-024-52382-7 (PMC11405393; doi:10.1038/s41467-024-52382-7)
Supplement: Supplementary file 4 — Source Data [file 41467_2024_52382_MOESM4_ESM.zip › Source Data/Source data-Supplementary Information.pptx]

## Slide 1
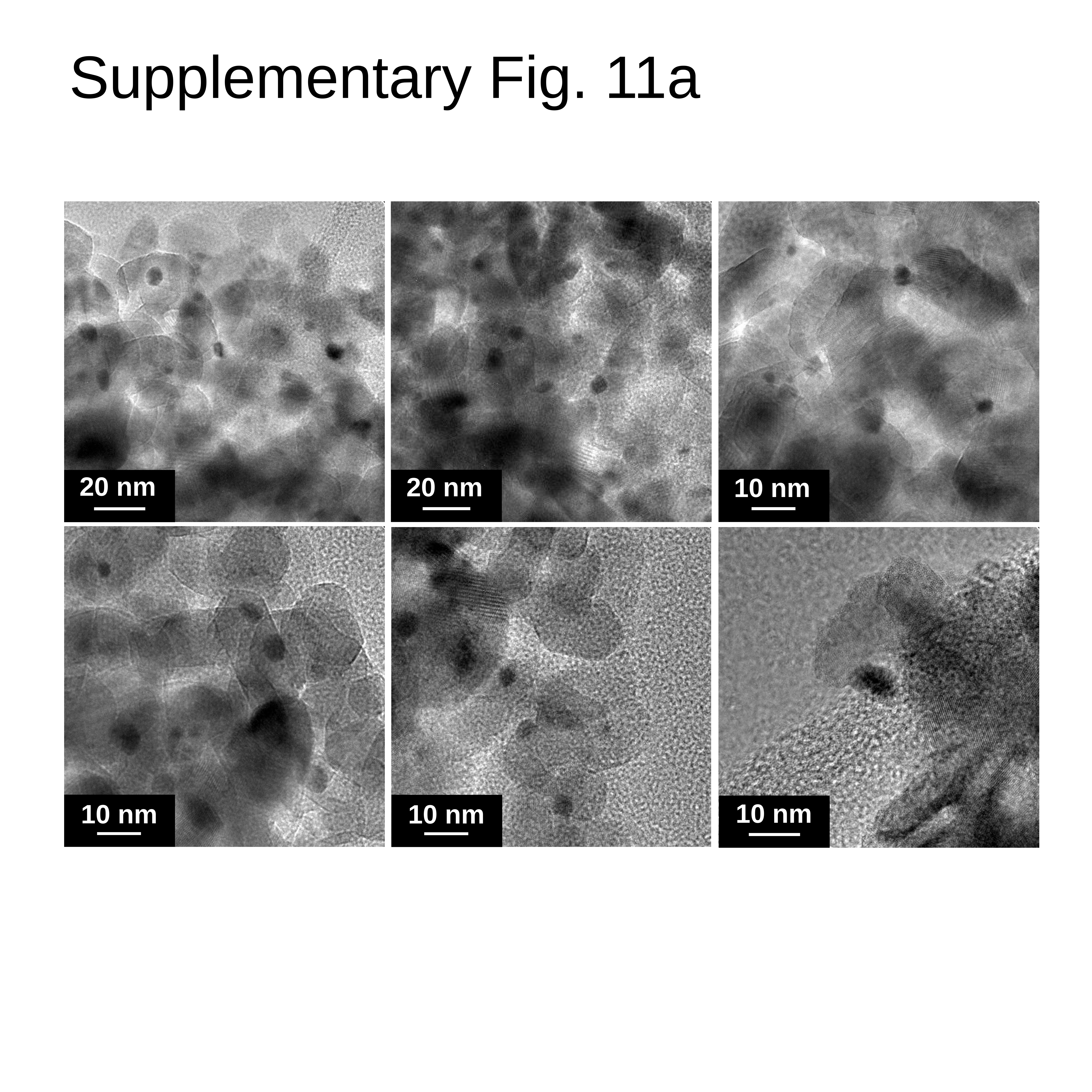

Supplementary Fig. 11a
20 nm
20 nm
10 nm
10 nm
10 nm
10 nm

## Slide 2
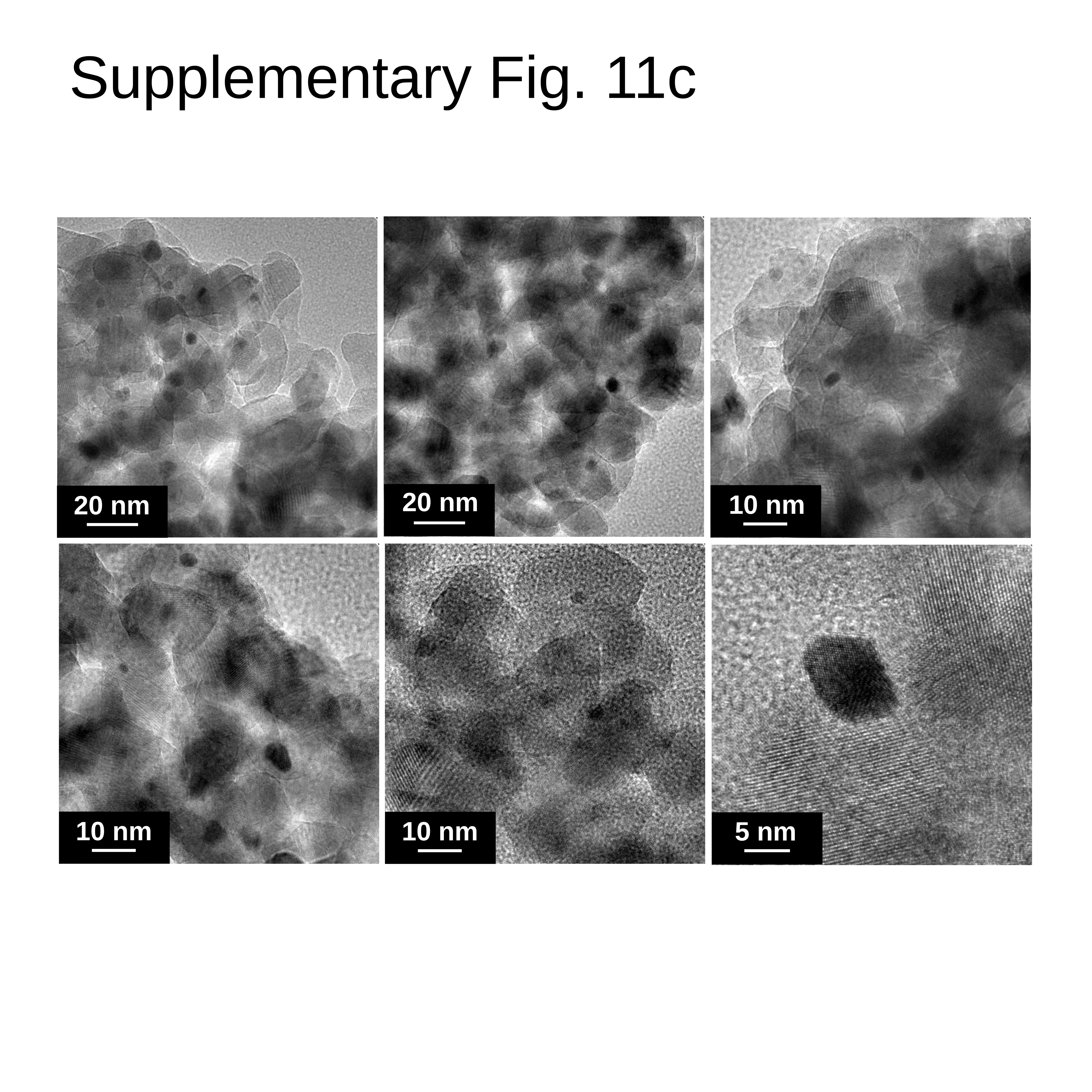

Supplementary Fig. 11c
20 nm
20 nm
10 nm
10 nm
10 nm
5 nm

## Slide 3
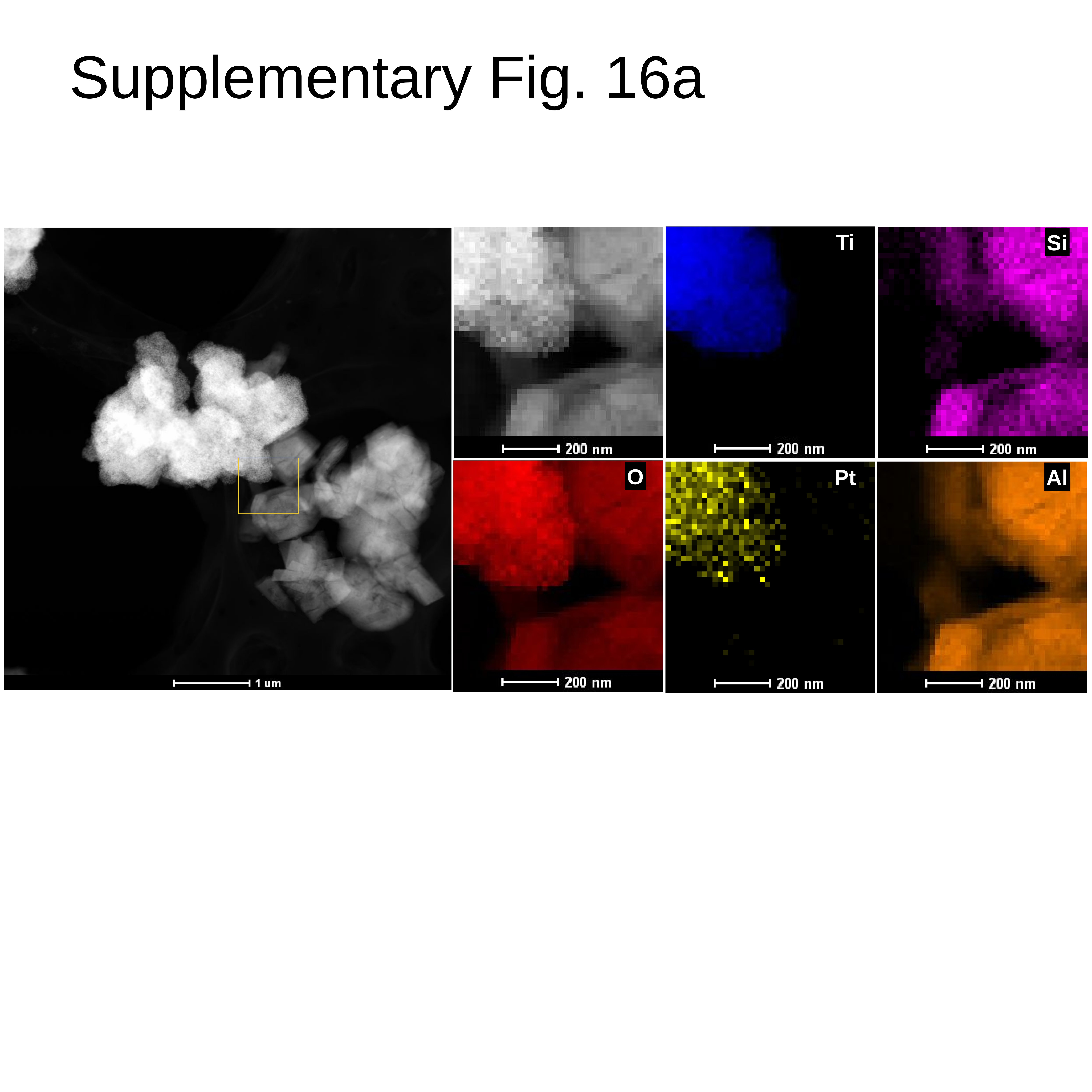

Supplementary Fig. 16a
Ti
Si
O
Pt
Al

## Slide 4
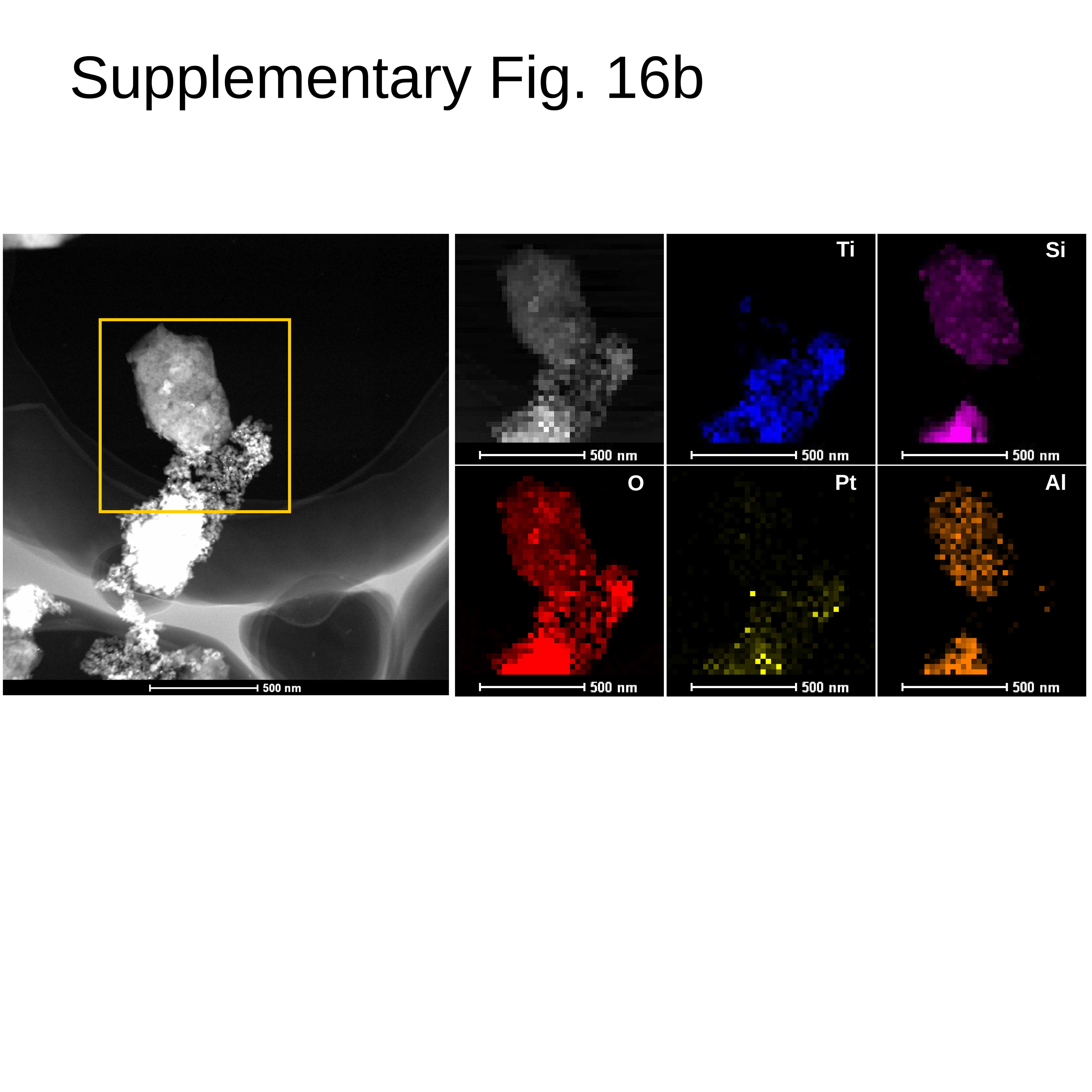

Supplementary Fig. 16b
Ti
Si
Pt
Al
O

## Slide 5
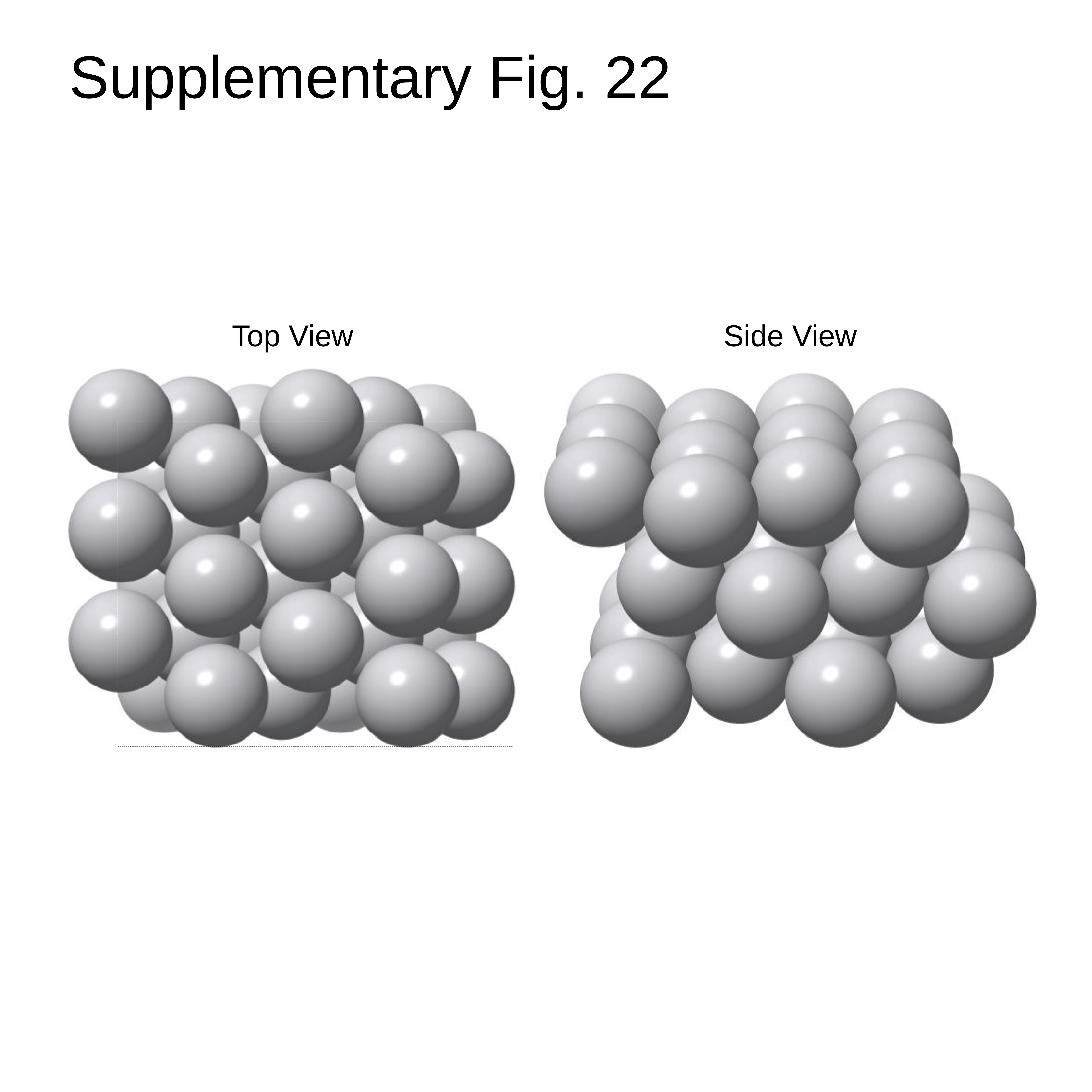

Supplementary Fig. 22
Top View
Side View

## Slide 6
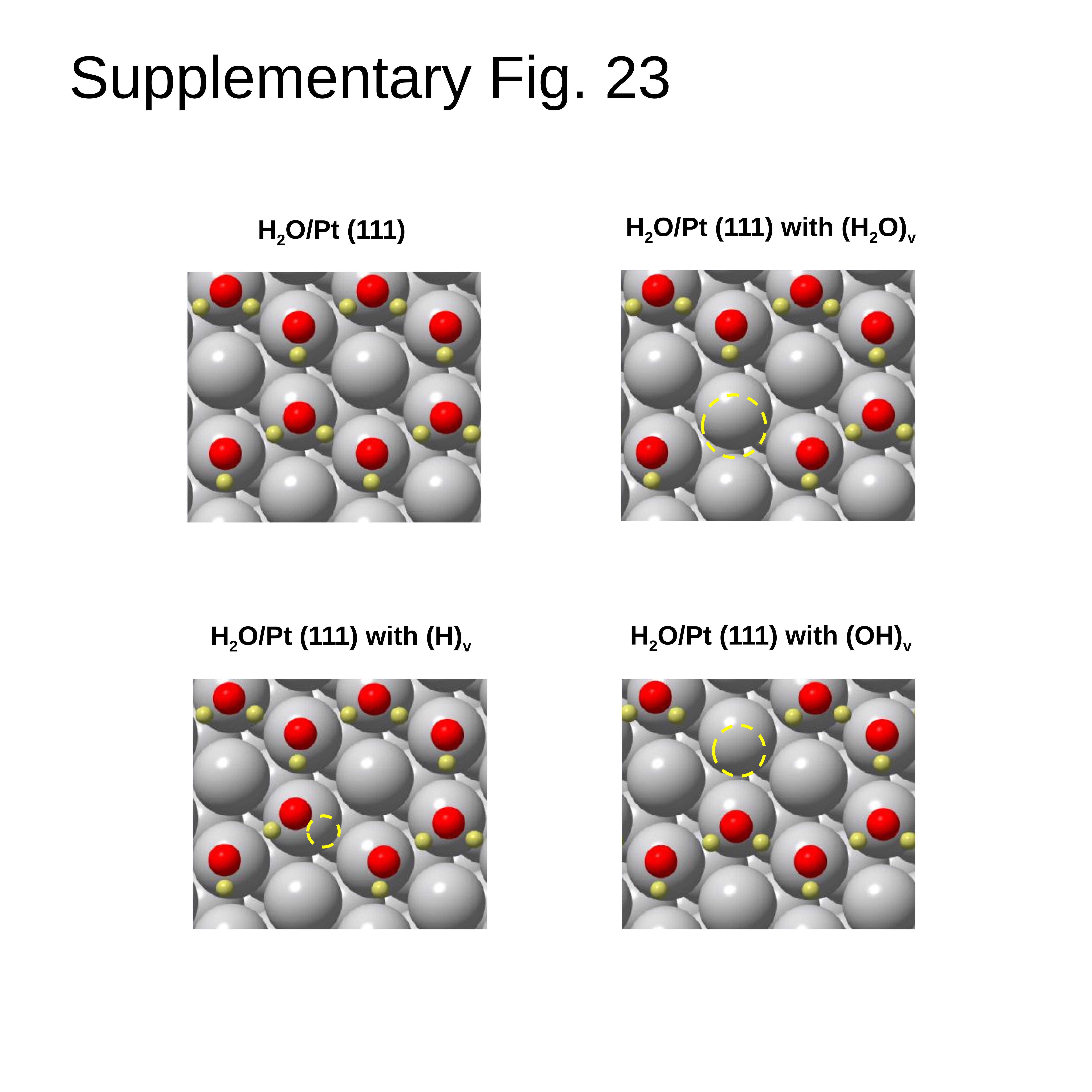

Supplementary Fig. 23
H2O/Pt (111) with (H2O)v
H2O/Pt (111)
H2O/Pt (111) with (OH)v
H2O/Pt (111) with (H)v

## Slide 7
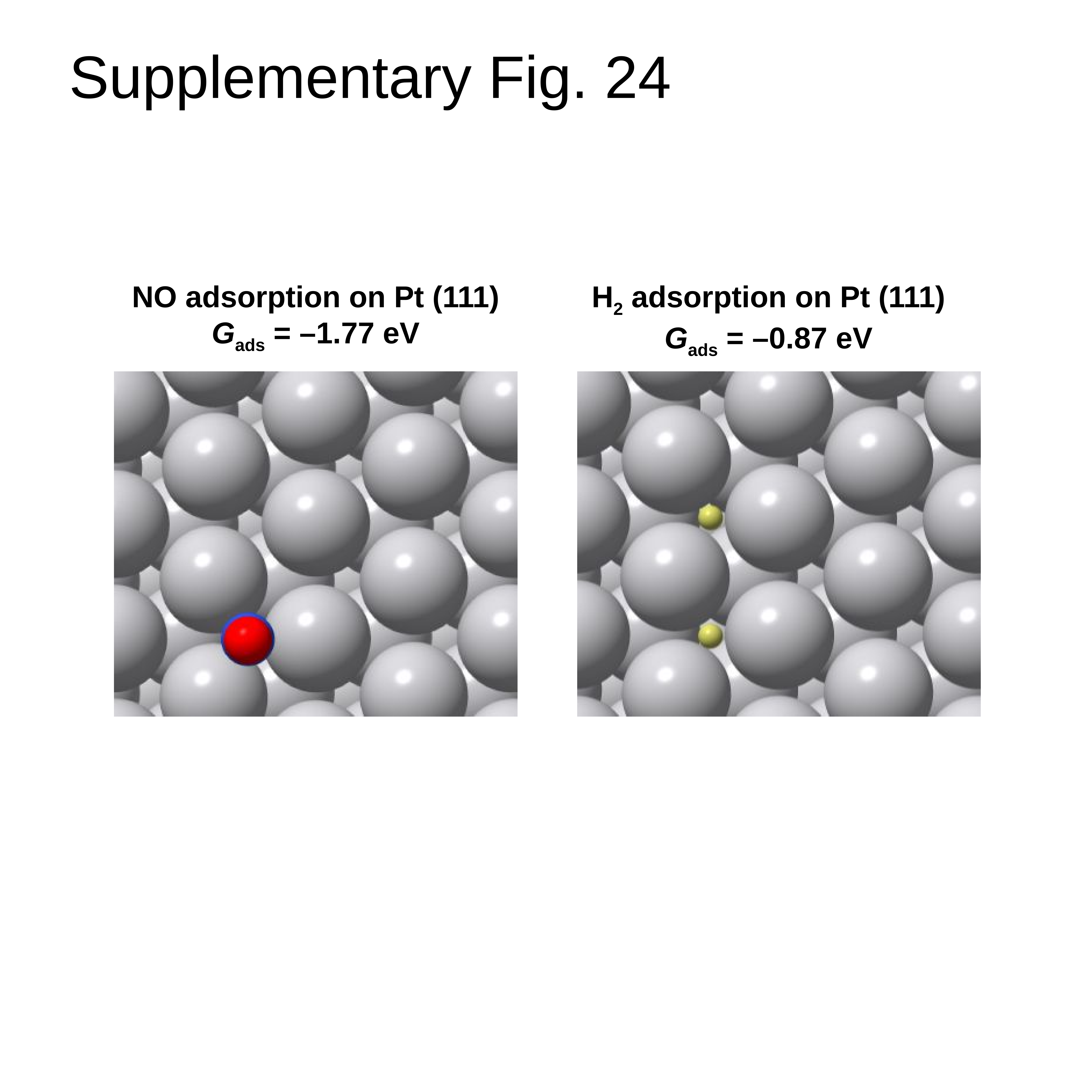

Supplementary Fig. 24
NO adsorption on Pt (111)
Gads = –1.77 eV
H2 adsorption on Pt (111)
Gads = –0.87 eV

## Slide 8
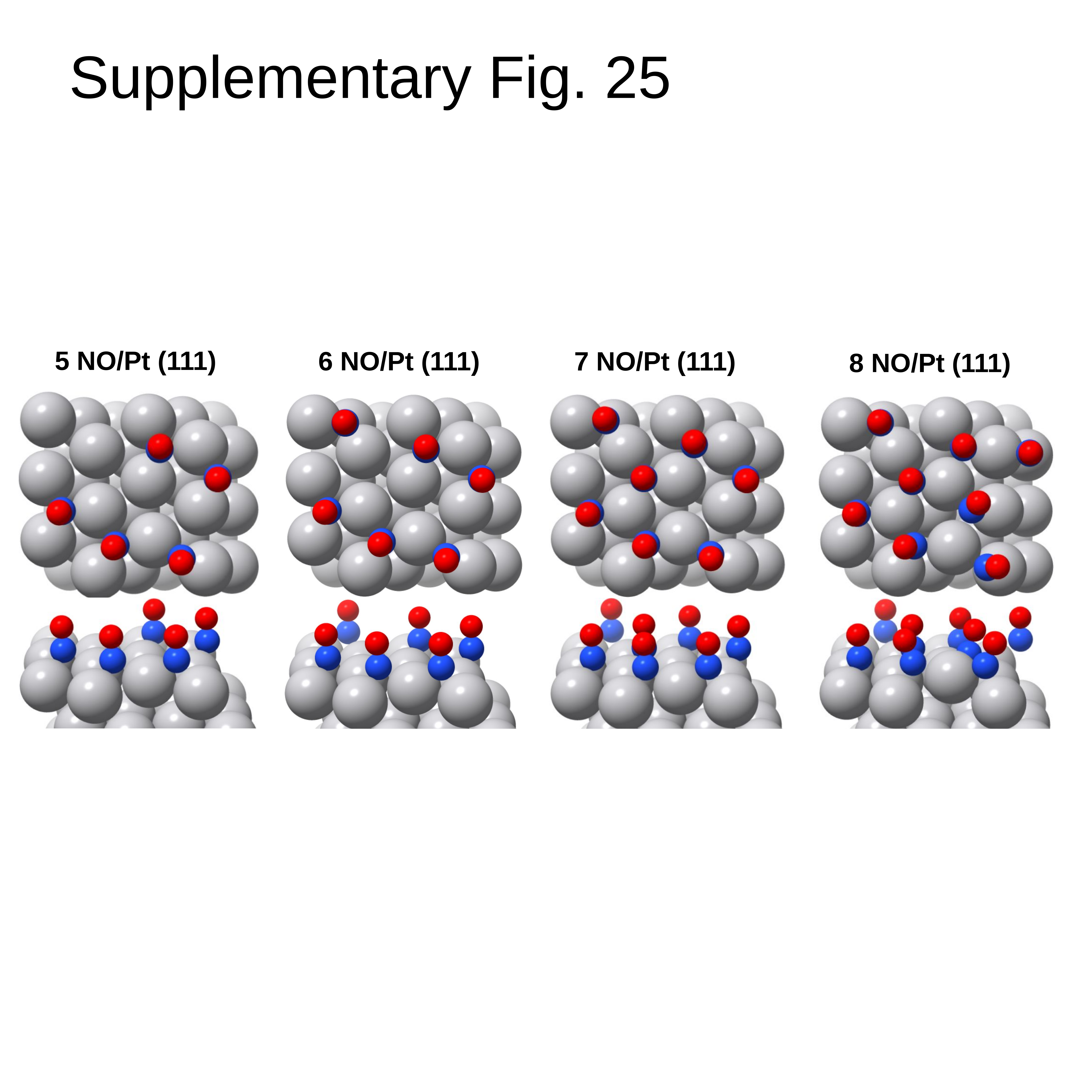

Supplementary Fig. 25
5 NO/Pt (111)
6 NO/Pt (111)
7 NO/Pt (111)
8 NO/Pt (111)

## Slide 9
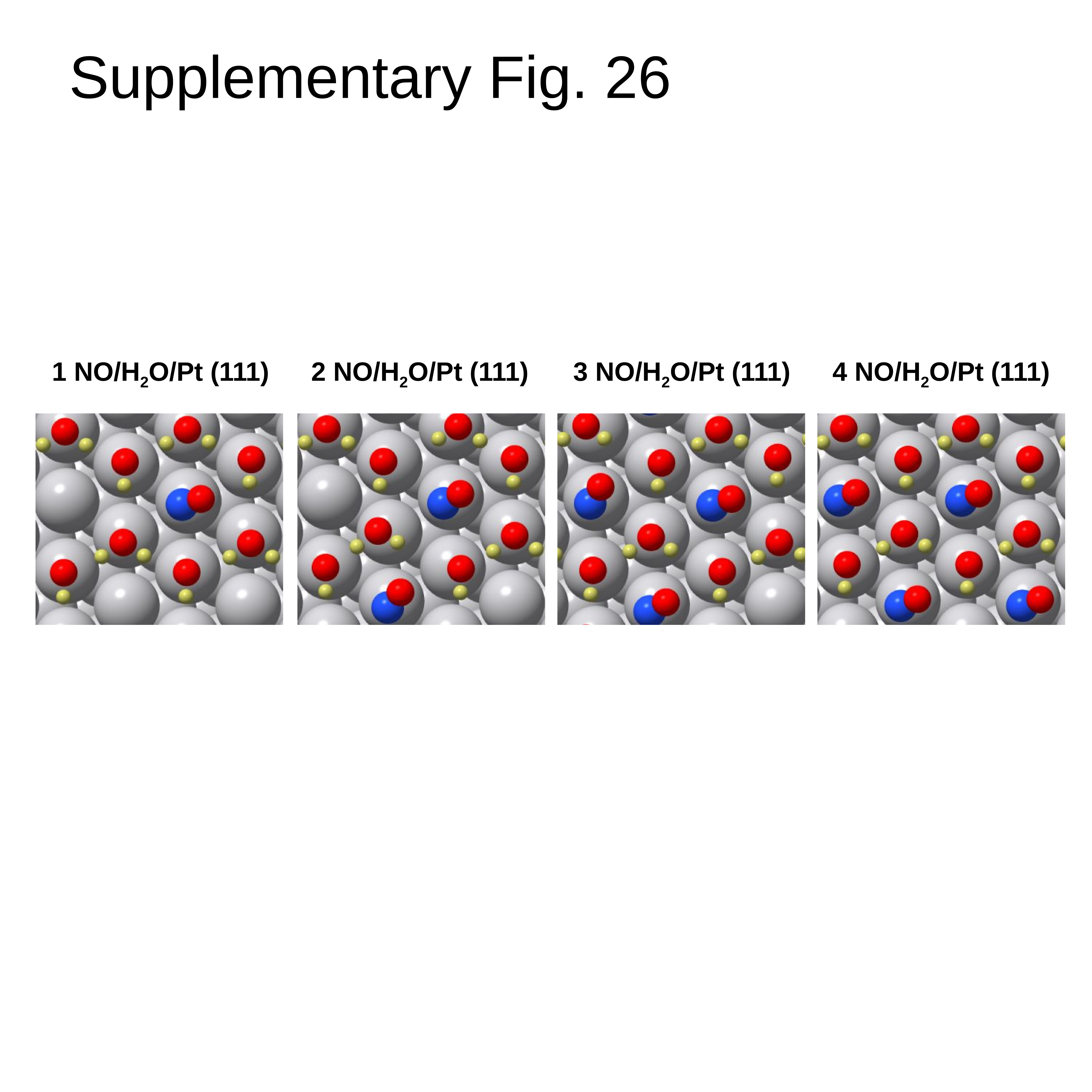

Supplementary Fig. 26
1 NO/H2O/Pt (111)
2 NO/H2O/Pt (111)
3 NO/H2O/Pt (111)
4 NO/H2O/Pt (111)

## Slide 10
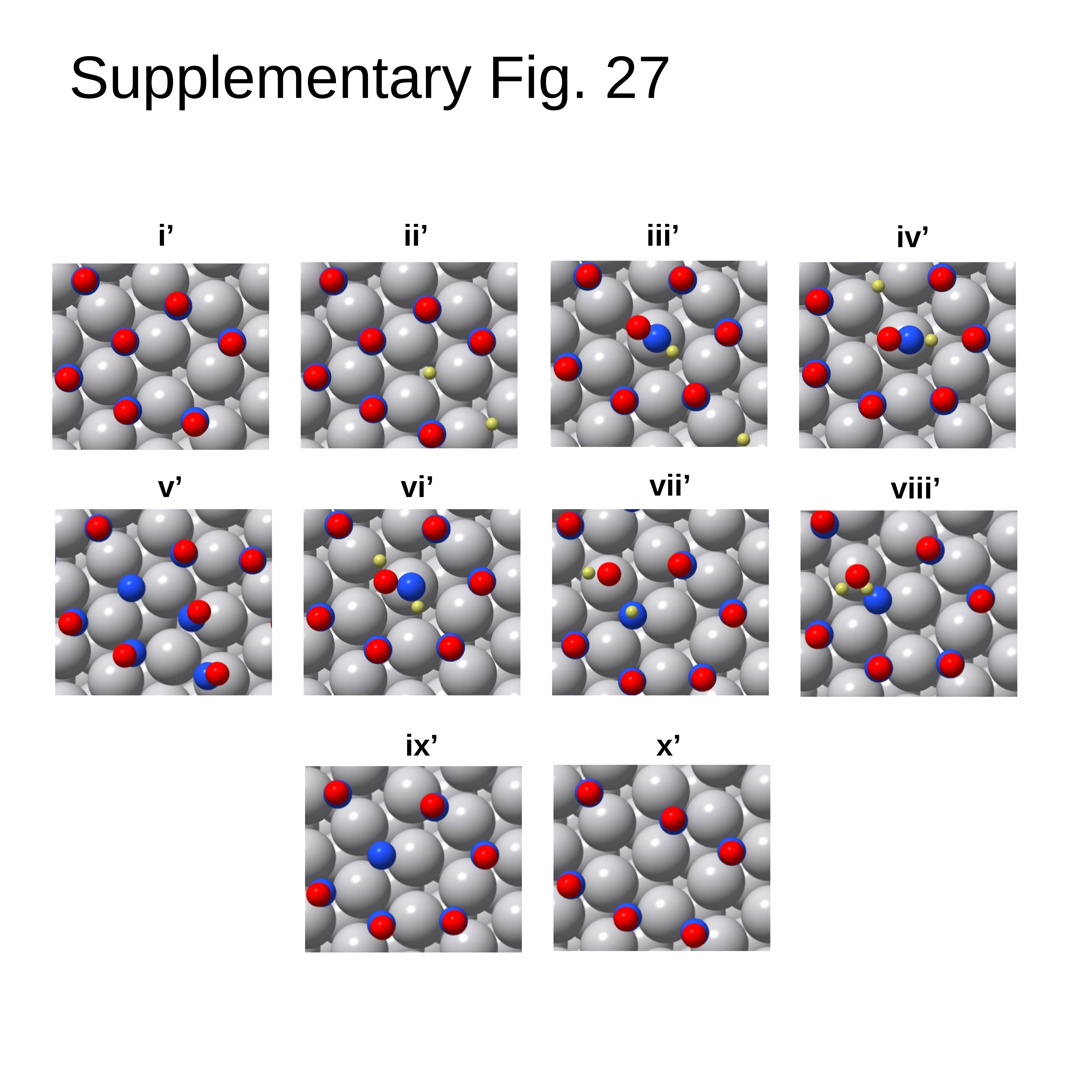

Supplementary Fig. 27
i’
ii’
iii’
iv’
vii’
v’
vi’
viii’
ix’
x’

## Slide 11
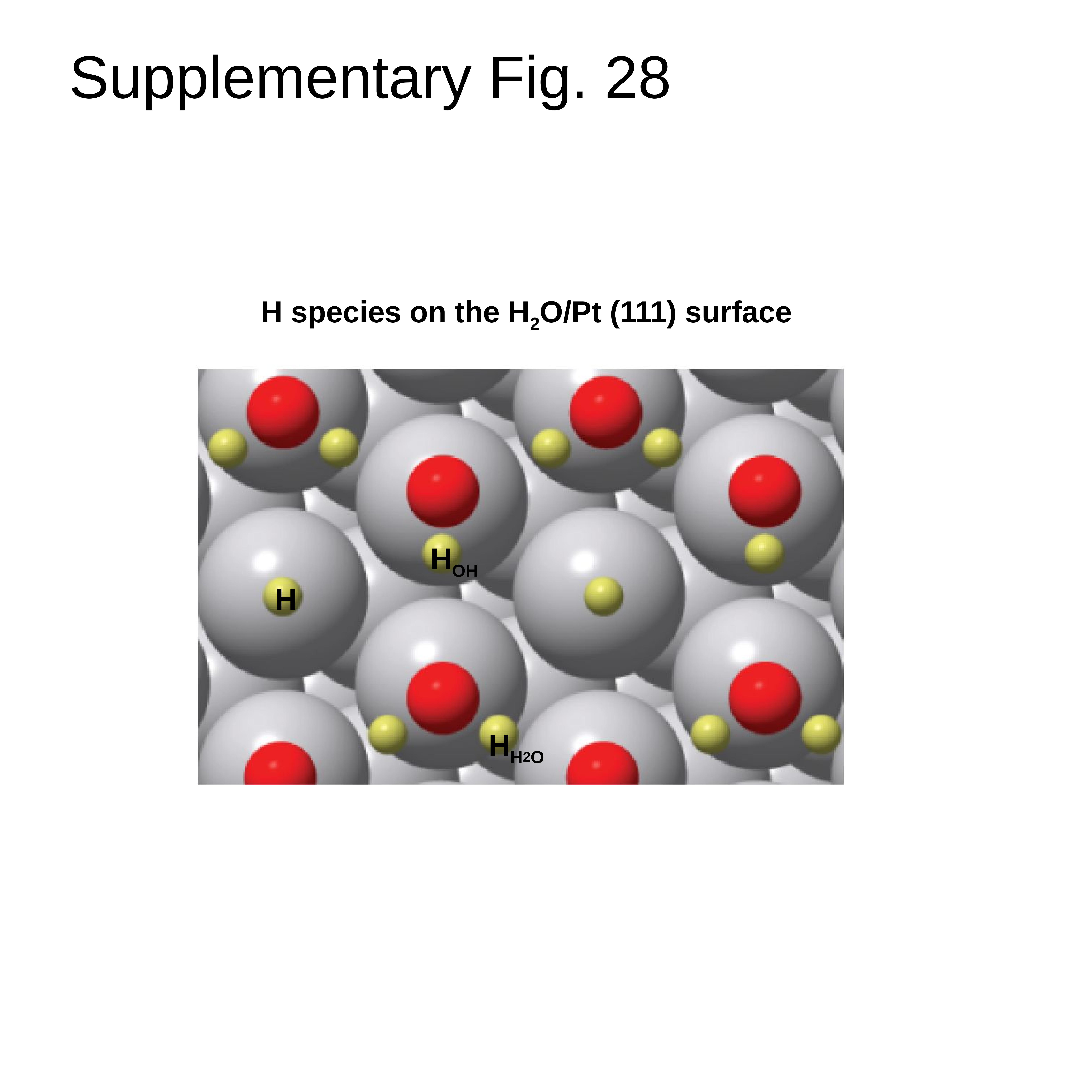

Supplementary Fig. 28
H species on the H2O/Pt (111) surface
HOH
H
HH2O
